# Supplementary material for: Understanding the onset of hot streaks across artistic, cultural, and scientific careers
Source: Nat Commun. 2021 Sep 13;12:5392. doi: 10.1038/s41467-021-25477-8 (PMC8438033; doi:10.1038/s41467-021-25477-8)
Supplement: Supplementary file 1 — Supplementary information [file 41467_2021_25477_MOESM1_ESM.pdf]

Supplementary information for understanding the onset of hot streaks across artistic, cultural, and scientific careers

Lu Liu,[1,2,3,4] Nima Dehmamy,[1,2,3] Jillian Chown,[1,3]

C. Lee Giles,[4,5] and Dashun Wang[1,2,3,6,*]

[1]*Center for Science of Science and Innovation, Northwestern University, Evanston, IL, USA.*

[2]*Northwestern Institute on Complex Systems, Northwestern University, Evanston, IL, USA.*

[3]*Kellogg School of Management, Northwestern University, Evanston, IL, USA*

[4]*College of Information Sciences and Technology, Pennsylvania State University, University Park, PA, USA.*

[5]*Department of Computer Science and Engineering, Pennsylvania State University, University Park, PA, USA.*

[6]*McCormick School of Engineering, Northwestern University, Evanston, IL, USA.*

[*] Corresponding author: dashun.wang@northwestern.edu

CONTENTS

Supplementary Note 1 Data and method 5
Supplementary Note 1.1 Artists 5
Supplementary Note 1.1.1 Data 5
Supplementary Note 1.1.2 Method 6
Supplementary Note 1.2 Directors 9
Supplementary Note 1.2.1 Data 9
Supplementary Note 1.2.2 Method 10
Supplementary Note 1.3 Scientists 11
Supplementary Note 1.3.1 Data 11
Supplementary Note 1.3.2 Method 11
Supplementary Note 1.4 Limitations 12
Supplementary Note 1.5 Impact distribution for auction prices and citations . . 13
Supplementary Note 1.6 Interpretation of exploration and exploitation in different domains 14
Supplementary Note 2 Related work 14
Supplementary Note 2.1 Art style analysis 14
Supplementary Note 2.2 Exploration and exploitation 16
Supplementary Note 3 Robustness check 21
Supplementary Note 3.1 Different timing of hot streaks 21
Supplementary Note 3.2 Different levels of impact 21
Supplementary Note 3.3 Different disciplines 21
Supplementary Note 3.4 Individual fixed effect 22
Supplementary Note 3.5 Regression analysis 22
Supplementary Note 3.6 Scientists with two hot streaks 23
Supplementary Note 3.7 Scientists with hot streaks at the beginning of their career 23
Supplementary Note 3.8 Alternative community detection method 24

Supplementary Note 3.9 Different time window 24
Supplementary Note 3.10 Alternative diversity index 24
Supplementary Note 3.11 The number of styles/topics 25
Supplementary Note 3.12 The faction of works on the most popular style/topic . 25
Supplementary Note 3.13 Switching topics 26
Supplementary Note 3.14 Episodes of exploration and exploitation 26
Supplementary Note 3.15 Different numbers of centroids 26
Supplementary Note 3.16 Papers without references 26
Supplementary Note 3.17 Different style measurements 27
Supplementary Note 4 Scientific teams 27
Supplementary Note 4.1 Regression analysis on team size 27
Supplementary Note 4.2 Different disciplines 28
Supplementary Note 4.3 Team composition 29
Supplementary Note 4.4 Self citations 29
Supplementary Note 4.5 Controlling for effects of collaborations 30
Supplementary Note 5 Characteristics of exploited topics during hot streak . . . 30
Supplementary Note 5.1 Exploration, exploitation and normal phase 30
Supplementary Note 5.2 Recency 31
Supplementary Note 5.3 Popularity in a career 31
Supplementary Note 5.4 Impact 32
Supplementary Note 5.5 Popularity in embedding space 32
Supplementary Note 5.6 Predicting topic exploited 32
Supplementary Note 6 Testing alternative hypothesis 33
Supplementary Note 6.1 Multiple publications 33
Supplementary Note 6.2 New research direction 33
Supplementary Note 6.3 Collaboration with high-impact scientists 34
Supplementary Note 6.4 Changing institutions 34
Supplementary Note 6.5 Research support 34

Supplementary Note 6.6 Future hot topic 35
Supplementary Note 6.7 Exploiting previously successful work 35
Supplementary Note 6.8 An autoregressive process 36
References 38

Supplementary Note 1. DATA AND METHOD

In this paper, we constructed large-scale datasets for creative products from a wide range of disparate sources, including images of artworks for artists, film plots and casts for directors, and publication and citation information for scientists, along with their impact measures of auction prices, IMDB ratings and paper citations, respectively [1]. We developed computational tools from deep learning and network science and learned high-dimensional representations for these creative products, allowing us to trace an individual's career trajectory on the underlying creative space around the beginning of a hot streak. In this section, we describe in detail how we construct the data and how we learn the representation for the three domains.

Supplementary Note 1.1. Artists

Supplementary Note 1.1.1. Data

We collect for artists large-scale image datasets tracing art styles, and auction datasets tracing the career impact, to quantitatively understand artistic success through the work one produced. We first gather over 800K images of visual arts from two databases, Art500K [2, 3] and Artnet (www.artnet.com). Art500K contains large-scale images of artworks collected from several online sources and museum collections such as Rijks Museum and Wikiart, covering artworks for over 600 years from medieval to contemporary art. The data contain information regarding disambiguated artist names, title, year of production for each artwork. In addition, Art500K offers art style labels for over 160K images, allowing us to train the art style embedding in the classification task. Artnet is an art market website with over 340K disambiguated artist profiles. The free version of the website records for each artist a list of gallery collections and auction records in the market, along with the image, title, and production year. If an artwork was produced over a span of several years, we use the last year as its production year, corresponding to the year in which the work was finalized. Together, we curated for artists in Art500K and Artnet their image profiles with the list of images and year of production. To quantify the impact dynamics and career hot streaks, we further collect impact profiles of 3,480 artists who have at least 15 artworks and 10 years of career length by combining two art market databases, Artprice (www.artprice.com) and

Findartinfo (www.findartinfo.com). Building on prior work [1, 4], we curated for each artist the list of auction price and year of production for the work one produced, allowing us to measure the timing of hot streak for each individual with the auction price.

We compare each of the 3,480 artists who have impact information to the image profiles curated from Art500K and Artnet. Two artists are considered to be the same if they satisfy the following criteria: 1) Identical names. The last name and first initial are the same. If full names are available for both artists, they must be identical. 2) Active during the same period. The career span of the image profile, measured by the production year of the first and last artwork, is within 10 years of the career span of the impact profile. By applying this entity linkage procedure, we end up with 2,016 artists with impact sequence and at least 10 images for our analysis. Note that images from the Art500K mainly include artworks from museums that may not appear in the auction market. Previous studies show that the years during which artists produced artworks with high market value are aligned with the production years of high-value artworks considered by museum curators [4, 5], suggesting that the timing when an artwork was produced is useful to identify its relative impact within a career.

Supplementary Note 1.1.2. Method

We train an art style classifier with deep neural networks using images with ground-truth labels in Art500K [6]. If an image have multiple labels, we randomly pick one as its unique label. Art500K provides 230 unique art styles, with imbalanced sample size among them (Fig. 1). To improve the performance of the classifier, we ignore styles with too few images, and reduced the number of classes to art styles with over 1000 images (top 29). For classes with more than 5000 samples, we randomly selected 5000 images for each class. For classes with samples fewer than 5000, we increase their sample size to 5000 by applying image augmentation methods with rotation (±30), scale (1-1.2) and random crop (0.8-1). Together, we gathered a balanced dataset of 145K images for the classification task. We normalize each image by their mean and standard deviation. We randomly sample 80% of images as the training set and the rest 20% as the test set.

We build on a popular convolutional neural network architecture in art style analysis, VGG16 [7]. Prior studies show that a transfer learning approach in which a model is fine-tuned outperforms the model trained from scratch, as its filters are more interpretive and better capture lines and shapes learned from the object recognition tasks [6], prompting us to adopt the transfer learning method in this paper. Specifically, we remove the dense layers in the pretrained VGG16, added two hidden dense layers with ReLU activation (dimension = 4096 and 512, respectively) and a final classification layer of 29 nodes with softmax activation (Fig. 1A). The first dense layer learns the non-linear combinations of outputs from convolutional layers. The second dense layer further reduces the dimension for the output of the previous layer and learns the high-level content for each image to better identify different art styles. In the experiment, we fix the parameters of the convolutional layers and only fine-tune the weights in the three newly added layers. We train the model with Adam optimizer and batch of size 128, and selected the model with the best performance. The highest accuracy is 0.54 on the test set, which is significantly higher than the random accuracy for our balanced set (0.03). A recent study on art style classification [6] utilized 77K images with art style labels from Wikiart, and trained their model with unbalanced samples from 20 style classes. Their random accuracy is 0.17 and their best accuracy is 0.637, suggesting that the performance of our model is comparable to the state-of-art in terms of the accuracy improvement compared to random guess.

As art style is encoded in both low-level features like brush strokes and high-level features like content and themes [8], we combine both the low- and high-level embedding to represent the art style of each image. We first use the 512-dimensional dense layer to capture the high-level feature of each image. We extract the output from the 512-dimensional dense layer for all images in our dataset, and then apply principle component analysis (PCA) to reduce the 512-dimensional vector to a 100-dimensional vector. We further generate low-level features from the first and third convolutional layers in VGG16 ($d = 224 \times 224 \times 64$, $d = 112 \times 112 \times 128$, respectively). Different from the traditional Gram matrix kernel [2, 8] that flattens the output of convolutional layers to generate a huge vector, here we reduce the dimension of the two tensors by calculating the mean value for the second and third dimension, and generate a 224-dimensional vector and a 112-dimensional vector respectively. As the absolute value of the two vectors may not be comparable to compute the distance in the

embedding space, we further normalize the two vectors by their sample mean and standard deviation, and then concatenate the 224-dimensional vector from the first convolutional layer and the 112-dimensional vector from the third convolutional layer. To make sure that the low- and high-level features are of equal importance, we further reduce the low-level feature to 100-dimensional vector by applying PCA on the 336-dimensional vector. In the end, we normalize the low and high level embedding by their sample mean and standard deviation, and combine the two 100-dimensional vectors together to get a 200-dimensional representation for each image. We visualize the kernel density projected onto two dimensions with PCA for images from three art styles (Fig. 7). We then apply the k-means clustering to the 200-dimensional embeddings, and assigned style label for each image as the cluster it belongs to. In the main text we report results based on k-means clustering with $n = 30$ centroids. We show in Fig. 34 that our results are robust to different number of centroids.

To better understand the meaning of each layer, and to test if the selected layers can indeed capture low-level brushes strokes and high-level content of an image, we offer several case studies on famous paintings to obtain more insights from the fine-tuned model. We analyze two positive samples, Number 1 by Jackson Pollock (Fig. 3) and Mona Lisa by Leonardo da Vinci (Fig. 4), and two negative samples, Kiss by Gustav Klimt (Fig. 5) and Café terrace by Vincent van Gogh (Fig. 6). Our model can successfully predict the abstraction expressionism for Number 1 and high renaissance for Mona Lisa, while it misclassifies Kiss, one of the most famous paintings in Art Nouveau, and Café terrace, a famous post-impressionism painting, to Surrealism. We visualize a random activation from the first and third convolutional layer for each image. The first convolutional layer learns to detect lines, dots and colors from raw pixels with 3×3 filters, and then passes these local patterns to deeper layers, which captures the brush strokes within a broader region of each image. For example, the dripping and splattering in Jackson Pollock's Number 1 are well captured by these low-level layers, and the uniform distribution of each pattern also reflect the balanced nature of his work. Compared to the local patterns coded in low-level layers, the activation of the convolutional layer in the last block and the last max-pooling layer show higher-level features such as the landscape, part of a body, and the roof of a house, which suggests that high-level features capture more about the content or object in an image. Finally, we visualize the saliency map for the predicted label, which highlights the important pixels that

the model utilized to make the decision. The two negative samples share common features in the saliency map, where the yellow regions in the background and the clothes for Kiss, and the yellow cafe house for Café terrace are highlighted, which illustrates why the two images may be classified to the same style.

Supplementary Note 1.2. Directors

Supplementary Note 1.2.1. Data

Our second setting traces the profiles of film directors from The Internet Movie Datavase (IMDB) (www.imdb.com/interfaces). The IMDB database contains one of the largest collections of film records for over 100 years worldwide, along with detailed information for each film such as title, release time, user rating and more. In addition, the webpage for each film includes sections for full cast and crew list, plot information, and genre tags, allowing us to learn the embedding of films. The IMDB database also provides a disambiguated profile for each director with a unique ID assigned to the list of work they produced. We focus on directors who have at least 10 years in career length and 15 films in IMDB. We use the IMDB rating to approximate the impact of each film and measure the timing of hot streak within a career with the rating sequence [1, 9]. Directors have wide-range responsibilities for a film, from pre-production to approving the final edit. They are also responsible for working with and overseeing scriptwriters as they work on the script as well as selecting and training the cast. Given the director's role in shaping these important elements of the film, we use data on the plot and casting of each film to analyze the careers of film directors. Specifically, we gather plot information from the storyline, plot summary and plot synopsis section from the IMDB webpages. If a film has multiple paragraphs from these sections, we merge them together to construct a single plot record for each film. We also gather for each film the full list of actors with their unique IDs. Given IDs in the IMDB database, we can identify if a film belongs to the hot streak period or not. In total, we collect 79K films by 4,377 directors for our analysis.

Supplementary Note 1.2.2. Method

Prior studies show that the storyline and casting information play an important role in the success of a film [10–14], prompting us to construct for each film the word embedding from plot summaries and node embedding from co-casting network. We first preprocess the plot text to learn the word embedding. We tokenize each plot into words and transferred all words to the lowercase. We remove punctuation and English stop words, stem each word, and only keep stemmed tokens with at least 3 characters. We train the word embedding with skip-gram model in our plot corpus from scratch, which captures the meaning of a word from its surrounding text in the plot corpus. We learn the 100-dimensional representation for each word with window size of 5 for 50 iterations, and calculate the plot embedding vector as the average value for all word vectors in each plot.

We further incorporate the casting information by constructing a co-casting network for the work produced by directors in our dataset. As IMDB ordered the cast by their importance, here we focus on top 10 featured actors. Two films are connected if they have common actors, with the link weight as the total number of actors they shared. We apply DeepWalk [15] to the co-casting network, a well-known representation algorithm for social networks which captures the local structures of nodes from random walks. We set the walk length as 10, the number of walks as 80, window size as 5, and learn the 100-dimensional node embedding. We further normalize the 100-dimensional plot and cast vector by sample mean and standard deviation, and concatenate the two vectors to get a 200-dimensional vector for each film. We visualize the kernel density for films from three different genre projected onto the two dimensional embedding space with PCA (Fig. 8). Similarly, we apply the k-means clustering to the 200-dimensional embeddings with their euclidean distance, and assign the style label for each film as the cluster it belongs to. In the main text we report results based on k-means clustering with $n = 30$ centroids. We show in Fig. 34 that our results are robust to different number of centroids.

To test if the learned embedding captures the film styles, we use the 200-dimensional vector as the input to predict the genre of each film. We formulate this task as a multi-label classification problem. Specifically, we build a fully-connected neural network with

two hidden layers (100d and 50d). We randomly split the data into training (80%) and test set (20%), and use the Adam optimizer to train the model with batch size of 128 for 20 epochs. Although we do not utilize any genre information to learn the film embeddings, they can successfully predict film genres with an accuracy of 0.948 (Fig. 9A). We repeat the classification task for word embedding and cast embedding separately (Fig. 9B-C), and find that they can also achieve a high accuracy (0.94 for both cases). And combing the two leads to even better performance.

Supplementary Note 1.3. Scientists

Supplementary Note 1.3.1. Data

In our third setting, we analyze publication records of scientists by combining Google Scholar (GS) profiles with Web of Science (WoS) [1]. GS allows individual scientists from diverse disciplines to create, maintain, and update their publication profiles. Assisted by its disambiguation algorithms, GS profiles provide a state-of-art disambiguation method to assemble publication list for individual scientists, offering unique opportunities to study scientific careers [16, 17]. We collect scientists' profiles and matched each publication record to the WoS database, which provides publication metadata and citation records for around 46 million journal papers since 1900. We curate for each scientist a list of papers with unique WoS identification numbers, publication date, and citation in 10 years C_{10} to approximate the impact of each paper, yielding 20,040 scientists with at least 15 papers and 20 years of career length for our analysis. We measure the timing of hot streak for these scientists using the logarithmic of C_{10} for the sequence of work they produced [1].

Supplementary Note 1.3.2. Method

We identify the topics of over 1 million papers published by these scientists using a novel network method introduced by Zeng and colleagues [18]. Here, we extract the reference list of each paper from the WoS with unique WoS ID, and focus on papers with at least one reference as recorded in the WoS. We construct a weighted co-citing network among papers produced by each scientist. Two papers are linked if they have common references, with the link weight as the total number of references they shared. We apply community

detection algorithm to the ego co-citing network for each scientist and assign the topic of a paper as the community it belongs to. In the main text, we report results using the community detection methods. To examine the robustness of our results, next we follow the same methodology for artists and directors to learn an embedding for papers with neural networks.

We construct a co-citing network among 1 million papers published by individuals in our dataset. Two papers are linked if they have common references, with the weight indicating the number of references they share. To speed up the network embedding algorithm, we first reduced the size of its adjacency matrix A with truncated SVD to 500 dimensions, where $A = U^T \Sigma V$. Fig. 10 shows the cumulative variance explained. There is no obvious saturation after a certain n, suggesting all components contain important information for the matrix. We then use the subject label in WoS to train a neural network and learn the representation of each paper. We focus on the top 34 subjects with more than 10,000 papers in the dataset, and ignore subjects with too few papers in this case. We randomly sample 10,000 papers for each subject if it contains more papers to make a balanced training set. We randomly split the data into training (80%) and test set (20%). We train a neural network with one hidden layer (64d). The accuracy is 0.515 for the test set (Fig. 12). We use the 64-dimensional vector from the hidden layer to represent the topic information of a paper. We visualize the kernel density for papers from three different disciplines in the embedding space (Fig. 13). We apply k-means clustering to the 64-dimensional embedding space with $n = 60$ centroids, and assign the topic of each paper as the community it belongs to. We repeat our analysis on $\langle H \rangle$ for topics measured from the embedding space, finding our conclusions remain the same (Fig. 14).

Supplementary Note 1.4. Limitations

Although our datasets capture among the largest collections of career profiles, there are limitations of the data that readers should keep in mind. For instance, the individuals in our datasets are "survivors" with long enough careers. This design is meant to be consistent with the previous study [1] so the patterns we observe here are comparable across the literature. Nevertheless, it also means that we cannot eliminate the potential survivorship bias, as some individuals and works may have been filtered out before the analysis. For example, famous

works may be overrepresented in museum exhibitions. This issue is mitigated somewhat by data collected from online art database Artnet, which contains art images from dealers such as galleries and auction platforms. By combining images from the two datasets it allows us to construct a more comprehensive profile for artistic careers, especially for the less famous works an individual produced. But still, potential biases may persist. For example, the coverage of one's entire career history may vary across individuals, and famous artists may have a better coverage overall than their less famous counterparts. Artworks by influential artists are more likely to appear in the art market or in the museum collection, whereas for less famous artists, the museums and art markets may only include their best work. Similarly, the coverage of artworks within each career might not be uniform, possibly biased toward the famous work one produced. This could lead to a potential downward bias for the exploration phase before the hot streak but a potential upward bias for the exploitation results during the hot streak.

Supplementary Note 1.5. Impact distribution for auction prices and citations

In this study we use the logarithmics of auction price and citations $\log C_{10}$ to approximate the impact of works for artists and scientists. The choice of logarithm is consistent with the hot streak model [1] that the impact of each individual was randomly draw from normal distributions. Prior literature [1, 16, 19, 20] offers empirical evidence that the raw auction price and raw C_{10} can be approximated by a log-normal distribution $P(x) = \frac{1}{x\sqrt{2\pi\sigma}} \exp\left[-\frac{(\log x-\mu)^2}{2\sigma^2}\right]$. As such, the distribution of logarithmic auction price and $\log C_{10}$ follows narrow distributions. Moreover, the IMDB rating for films directors also follows a narrow distribution ranging between 1 and 10. Taking the logarithm of the auction price and paper citation also allows us to study the impact across three domains in a consistent manner.

Supplementary Note 1.6. Interpretation of exploration and exploitation in different domains

The exploitation strategy in art reflects paintings that are similar in style. e.g. the artworks may contain similar objects, convey similar themes, or use similar skills/techniques. This corresponds to famous examples such as van Gogh's series of sunflowers, Picasso's blue period and its theme of poverty and despair, Warhol's repetition in the use of objects, and more. Exploitation in film directing reflects a pattern of making similar films in succession, which may share similar story lines and/or characters. Film sequels are typical examples: they share similar stories with largely overlapping characters and casts, and they typically belong to the same genre. By contrast, exploration corresponds to films with diverse styles as well as different stories, characters, and genres. Similarly, exploitation in science involves papers on similar research topics or using similar methodologies or techniques, whereas exploration engages experimentation on diverse ideas and areas of research.

Supplementary Note 2. RELATED WORK

Supplementary Note 2.1. Art style analysis

In this section, we briefly summarize related work on art image analysis from three directions: 1) statistical patterns of art styles, 2) deep neural networks (DNN) and style classification, and 3) neural style transfer.

Statistical patterns of art styles This line of research focuses on providing quantifiable information on art styles by looking at different visual properties of the images [21]. Researchers proposed various metrics from local patterns of images to identify art styles or artists, ranging from the fractal dimensions [22–25] to edge and shape statistics [26] to color usage [27–29]. Early studies mainly involve with small samples, consisting of several thousand images or less, and focus on case studies of certain art styles or artists [22, 24, 26]. More recent work started to cover images from different art movements thanks to the development of digital libraries on artworks such as Web Gallery of Art and Wikiart. For example, Sigaki *et al.* analyzed art history from images in Wikiart [30], and measured the entropy and complexity of local image patterns, and found identifiable trend of art style

evolution in the complexity–entropy plane. Although this line of research provides insights to quantitatively understand art styles, researchers usually need to design meaningful local features that can better identify different art styles, the process of which is largely accelerated by the DNN studies that can automatically learn image representations.

DNN and style classification One productive line of research uses DNN to learn a better representation of images with large-scale labeled images of visual arts. Wikiart dataset is a popular dataset for such tasks [6, 31–35]. In addition, more recent studies curated large-scale, structured datasets on images of visual art by combining different online sources, and increased the number of art images to over 500k [2, 3, 36]. These studies built upon popular DNN architectures such as VGGNet [7], ResNet [37] and AlexNet [38] in object recognition, and fine-tuned models pre-trained on ImageNet or trained these models from scratch [2, 6, 31, 33–35, 39]. Although DNN improves the accuracy of art style classification with high-level representations, researchers find that the joint embedding from both high-level vector and the output of convolutional filters better captures the art styles [2], and adding outputs from convolutional layers to the high-level embedding also outperforms the high-level embedding in style and artist classification [32, 33], prompting us to use the joint embedding for style representation in this paper.

Neural style transfer Another line of research examines the art style analysis using neural style transfer [40]. The seminal work by Gatys *et al.* [8] first proposed to use VGGNet to convert an natural image into the art style of a target painting by optimizing the content information of the original image coded in high-level filters and the art style of the target painting coded in low-level filters. Since then researchers have studied various methods to speed up the transfer process [41, 42] and apply this method to different scenarios (e.g. general-purpose image-to-image transfer) [43] and different types of inputs (e.g. videos) [44]. More recent studies also use generative adversarial networks (GANs) for style transfer [45], and extend the input from a single image to an embedding space of styles. GAN has also been used to generate creative artworks that deviate from the trained distributions [46].

Supplementary Note 2.2. Exploration and exploitation

The trade-off between exploration and exploitation — and its relationship to creativity and learning — has been discussed extensively across a broad set of disciplines, ranging from computer science [47–49], to psychology [50, 51], to neuroscience [52, 53], to computational social science [54–56], to strategic management and organization theory [57–61]. On the one hand, producing creative and high-impact works requires one to explore new and diverse ideas, especially given the combinatorial nature of innovation and technology. Yet, exploration often comes at the cost of productivity. Exploitation, on the other hand, can support a focused agenda which is essential for developing existing knowledge. The trade-off between exploration and exploitation represents an enduring dilemma for individual and organization learning, motivating a large body of literature to examine exploration and exploitation from both theoretical and empirical perspectives. In Table. S1, we offer a brief overview of three relevant lines of research inquiring individual behavior, organization learning, and idea formation. We next discuss these directions in more detail.

Individual behavior At the individual level, the 'essential tension' hypothesis by Thomas Kuhn [62] illustrates the choice between exploiting existing ideas and exploring new yet risky opportunities. The sociology of science offers several fundamental theoretical discussions [63, 64]. More recently, empirical anlaysis has been conducted to quantitatively understand the 'essential tension' hypothesis. For example, Foster *et al.* [55] analyzed millions of abstracts from MEDLINE, and identified topics from the clusters on the chemical network to trace the research strategy of biomedical researchers [65]. In addition, the PACS code in American Physical Society (APS) dataset has also been widely used to quantify exploration and exploitation for scientific careers [18, 54, 66].

Researchers have also studied various environmental, social and individual factors that may influence one's choice between exploration and exploitation [50]. Environmental factors include resource status of a local position [51, 67], cost and reward of exploration and exploitation [67, 68], available information on different options [69], and more. Discussions centered around how long individuals should stay in the exploitation/exploration phase and when to change their behaviors under different environmental settings. For example, the

probability of exploration increases when the resource is depleted, when the cost of exploration decreases, or when individuals are uncertain about the options. The social factors are widely discussed in social learning strategies and collective intelligence [70–74], ranging from task complexity [75], to past success and failure [73, 75] to network structures [76, 77]. Individuals can update their strategies like exploration, exploitation or copying others to increase their payoffs under different settings. Individual factors such as personalities [78], cognitive capacity [79], and aspiration level [80], also influence one's propensity to explore or exploit.

In the literature of strategic management and organization theory, scholars have examined exploration and exploitation behaviors of individuals and firms, particularly focusing on the effects this has on organizational outcomes. For example, Singh & Agrawal [81] found that when scientists begin working within a new organization, the organization increases their use of the new recruit's prior work and that the majority of the effect is due to the employee's own exploitation of their prior work. Groysberg & Lee [82] found that when star security analysts were hired to explore (i.e., to initiate new activities for the organization), they experienced a drop in performance; whereas star security analysts hired to engage in exploitation (i.e., to reinforce the organization's existing activities) experienced a boost in performance. Other research has looked at the antecedents of individuals' exploration and exploitation behaviors. For example, Lee & Meyer-Doyle [83] examined how financial incentives shaped the behavior of sales people and found that individuals engaged in more exploration when performance-based incentives were weakened but this increase was driven by the organization's strongest performers. Recent study on network oscillation for bankers [84] suggests that switching between exploration and exploitation has positive effects on the employee's network advantage.

Organization learning, design and adaptation At the macro level, another important line of research examines exploration and exploitation in the context of organization learning, organization design, and organizational adaptation [60]. This line of work builds on the canonical work by March [59], and suggests that both exploration and exploitation are critical for an organization's performance, but they are inherently in tension and that this tension must be actively managed [85]. This tension reflects trade-offs between short vs.

long-term performance and stability vs. adaptability [59, 86–89]. Debates in this literature center on several fundamental questions: Do exploration and exploitation exist as two ends of a continuum (and so cannot coexist at the same time) or are they orthogonal discrete choices? Can organizations find a balance between exploration and exploitation activities or should they specialize in one or the other in terms of organizational, temporal or domain separation. It also explores the antecedents to organizations' decisions to pursue exploration or exploitation [61, 90], examining environmental factors (e.g., exogenous shocks, competitive dynamics) as well as organizational factors (e.g., culture, resources, capabilities) that influence that choice. This literature also uses the notion of organizational ambidexterity to describe the ability to do both exploration and exploitation simultaneously [91]. Finally, this research examines the performance implications for organizations of adopting different approaches to balancing this enduring trade-off between exploration and exploitation [92]. This line of research is performed using multiple different methodologies including empirical studies using quantitative and qualitative data from organizations, theoretical models [93], and agent-based simulations [61, 94, 95].

The temporal separation between exploration and exploitation discussed in this line of research is related to our findings. The temporal separation indicates that organizations transit from exploration to exploitation from time to time and vice versa. The intuition behind temporal separation is that exploration may offer opportunities and promising directions for organizations to exploit later [60]. The idea of temporal separation is rooted in notion of punctuated equilibrium, which describes how organizations transform through cycles of stability and massive upheaval. But in practice, the transition between exploration and exploitation is considered to be difficult. For instance, if an organization is focused on one of the activities, that creates a path dependence for the organization and is a powerful source of inertia. Temporal separation is therefore thought to require an agile organization that can successfully transition between the two activities when needed. The literature also discussed the gradual and discontinuous shifts between exploration and exploitation [96–99], and found that a sudden transition may be harmful to the survival of an organization [98, 99].

Idea formation At a more micro level, the discussion of exploration and exploitation is particularly relevant to studies on idea formation and innovation process [100–102], which

models the mechanism of innovation as random walks on the network of ideas/landscape of solutions. In this setting, exploration and exploitation is usually defined as creating new path or reproducing existing ideas. For example, Iacopini et al [100] models the cognitive growth of knowledge in science for over 20 years and validate process with concept networks curated from WoS abstracts. Studies have shown that both existing knowledge and novel combinations are essential for producing high-impact scientific papers [103]. The discussion goes beyond science to innovation and technology as well. For example, Youn *et al.* [104] analyzed technology codes used by USPTO to quantify innovation strategy, finding a constant rate of exploration and exploitation in patent records.

Overall, our results contribute to these three lines of literature in several ways. First, by documenting the relationship between exploration, exploitation and career hot streaks, our results demonstrate broader relevance of the concepts of exploration and exploitation, extending beyond existing individual or organizational settings to the understanding of hot streaks and individual creative careers. At root, our results suggest the important role of both exploration and exploitation in individual careers. Curiously, across a wide range of creative domains, a major turning point for individual careers appears most closely linked with neither exploration nor exploitation behavior in isolation, but rather with the particular sequence of exploration followed by exploitation, which highlights our second contribution. Indeed, extant literature has documented the fundamental role of exploration and exploitation in creativity. Yet as creative behaviors, they have traditionally been considered either in isolation or in combination, but rarely in succession [55, 60]. This is especially the case for career-level analysis. Our results suggest a sequential view of creative strategies that balances experimentation and implementation may be particularly powerful for producing long-lasting contributions.

Year	Paper	Category	Topic
2020	Kang & Kim [105]	Organization	incremental and discontinuous transition between exploration and exploitation
2019	Aleta *et al.* [54]	Individual	topic evolution for scientific careers
2019	Shibayama *et al.* [106]	Individual	junior scientists' performance and their training strategy
2019	Zeng *et al.* [18]	Individual	topic evolution for scientific careers
2018	Iacopini *et al.* [100]	Idea	emergence of knowledge and innovation
2018	De Langhe *et al.* [107]	Individual	exploration, exploitation and scientific revolution
2018	Luger *et al.* [88]	Organization	dynamic balancing of exploration and exploitation
2017	Jia *et al.* [66]	Individual	topic evolution for scientific careers
2016	Loreto *et al.* [101]	Idea	emergence of knowledge and innovation
2016	Swift [98]	Organization	difficult leap between exploration and exploitation
2016	Piao & Zajac *et al.* [89]	Organization	repetitive versus incremental exploitation and impact on exploration
2016	Murdock *et al.*	Individual	the reading strategy of Charles Darwin
2016	Burt & Merluzzi [84]	Individual	oscillation of network structure between brokerage and closure
2015	Foster *et al.* [55]	Individual	essential tension in biomedical research
2015	Rzhetsky *et al.* [65]	Idea	the evolution of problem selection in in biomedical research
2015	Youn *et al.* [104]	Idea	combinatorial dynamic of exploration and exploitation in innovation
2015	Bateman & Hess [78]	Individual	scientists' personality and research strategy
2015	Krafft *et al.* [73]	Individual	collective intelligence and learning strategies
2015	Chin *et al.* [108]	Individual	age and search strategy
2015	De Langhe & Rubbens [109]	Idea	essential tension in science
2014	Holmqvist [86]	Organization	exploitation and exploration in inter-organizational learning
2014	Knudsen & Srikanth [95]	Organization	coordinated behaviors by multiple individuals
2014	Tria *et al.* [102]	Idea	dynamics of correlated novelties
2014	Spisak *et al.* [110]	Individual	age of leaders and search strategy
2014	Billinger *et al.* [75]	Individual	impact of task complexity on the trade-off
2014	Toyokawa *et al.* [72]	Individual	impact of social learning on the trade-off
2013	Uzzi *et al.* [103]	Idea	novelty and conventionality of knowledge and paper impact
2013	Berger-Tal *et al.* [56]	Individual	trade-off for project-levels and project-based strategies
2012	Hills *et al.* [79]	Individual	memory and individual searching strategies
2012	Posen & Levinthal [61]	Organization	exploitation and exploration in inter-organizational learning
2011	Molina *et al.* [111]	Organizations	goal of new product development and the strategy choice
2011	Singh & Agrawal [81]	Individual	inventors mobility and exploration/exploitation behaviors
2010	Fang *et al.* [112]	Organization	balance between exploration and exploitation
2010	Rakow & Newell [69]	Individual	available information and propensity to take risks
2009	Groysberg & Lee [82]	Individual	individual mobility and exploration/exploitation behaviors
2009	Weisberg & Muldoon [113]	Individual	search strategy on epistemic landscape
2008	Hau *et al.* [68]	Individual	the role of sample size on decision making
2008	Goldstone *et al.* [74]	Individual	collective behavior and different learning strategies
2007	Namara *et al.* [114]	Organization	trade-off in biotechnology firms
2007	Eliassen *et al.* [115]	Individual	lifetime expectancy and individual foraging strategies
2007	Sidhu *et al.* [116]	Organization	multidimensional search in supply, demand, and geographic space
2007	Lazer & Friedman [77]	Individual	network structure of exploration and exploitation
2007	Lee & Meyer-Doyle [83]	Individual	incentives and the behavior of sales people
2007	Parker *et al.* [80]	Individual	maximizers, satisficers, and preference to exploration
2006	Lavie & Rosenkopf [96]	Organization	balancing exploration and exploitation in alliance formation
2006	Siggelkow & Rivkin [94]	Organization	performance effects of balance between exploration and exploitation
2005	Jansen *et al.* [90]	Organization	ambidexterity of exploration and exploitation
2005	Auh & Menguc [92]	Organization	multiplicative interactions between exploration and exploitation
2004	Rothaermel & Deeds [97]	Organization	exploration and exploitation in alliance formation
2004	He & Wong [117]	Organization	ambidexterity of exploration and exploitation
2002	Burgelman [118]	Organization	long-term adaptive capability of a firm's strategy
2002	Zollo & Winter [99]	Organization	exploration, exploitation and knowledge evolution cycle
2001	Rosenkopf & Nerkar [58]	Organization	patenting activity and the external environment
2001	Sørensen *et al.* [87]	Organization	organizational aging and innovation process
1994	Henderson & Cockburn [57]	Organization	R&D behaviors and research productivity in pharmaceutical firms
1993	Levinthal & March [119]	Organization	exploration and exploitation in organizational learning
1991	March [59]	Organization	exploration and exploitation in organizational learning
1979	Kuhn [62]	Individual	essential tension in science
1976	Charnov [67]	Individual	optimal foraging behaviors
1975	Bourdieu [63]	Individual	scientific organizing framework and capital accumulation
1962	Polanyi [64]	Individual	scientific discoveries as puzzle solving

Supplymentary Table 1: List of key references on exploration and exploitation

Supplementary Note 3. ROBUSTNESS CHECK

Supplementary Note 3.1. Different timing of hot streaks

Does the observed relationship between exploration, exploitation and hot streak depend on when hot streak occurs within a career? To test this, we split artists, directors and scientists into early and late hot streak, and compare the distribution $P(H)$ for work produced before and during a hot streak for individuals with different timing of hot streak. We find that $P(H)$ during hot streak is significantly smaller than before for both cases, suggesting that our findings are robust to different timing of hot streaks (Fig. 15)

Supplementary Note 3.2. Different levels of impact

Does the observed exploration-exploitation transition apply to individuals with different level of impact? To test this, we identify for each individual the highest impact work within a career [1, 16], and group individuals by their highest impact into high- and low-level. We compare the distribution $P(H)$ for work produced before and during hot streak, finding that $P(H)$ during hot streak is significantly smaller than before for individuals with different level of impact across three domains (Fig. 16). We also calculate $\langle H \rangle$ for work produced before and during hot streak, and compare to the entropy distribution $P(\langle H \rangle)$ of the null model, finding again the same conclusion (Fig. 17).

Supplementary Note 3.3. Different disciplines

To test if our results apply to scientists from different disciplines, we identify for each scientist her discipline with subject categories provided by WoS, and group the subjects into six general disciplines: Physical Science, Biology, Medicine, Environmental Science, Chemistry and Engineering [120]. For each scientist, we count the number of papers published in each of the subject, and consider the one with the most publications as her home discipline. We repeat the analysis on $\langle H \rangle$ for scientists from each of the six disciplines, and compare to the distribution $P(\langle H \rangle)$ for 1000 realizations of the null model, finding our results remains the same (Fig. 18A-L) We also measure $P(H)$ for papers before and during hot streak in real careers, and reach the same conclusion (Fig. 18M–R).

Supplementary Note 3.4. Individual fixed effect

Individuals may have different baseline exploration rates. Do individuals with overall low career entropy show similar exploration-exploitation dynamics? To test this, we identify the typical level of exploration in each career, defined by the percentage of the unique number of styles/topics n_i over all works one produced (N), denoted as n_i/N. We group individuals into high and low exploration rates, and compare the distribution $P(H)$ for works produced before and after during hot streak. We find that the exploration-exploitation transition occurs for individuals with different levels of entropy across the three domains (Fig. 19). We also calculate $\langle H \rangle$ in real careers and compare to the entropy distribution $P(\langle H \rangle)$ of the null model, and find that our results remain the same across three domains (Fig. 20). We further compare the level of entropy change by measuring the distribution $P(H)$ of real careers over that of the null model, denoted as $R(H) = P(H)/P_r(H)$ for works produced before and during hot streak (Fig. 21). We find that individuals tend to deviate from their typical strategy around the beginning of a hot streak: individuals who tend to exploit on average become more exploratory before a hot streak begins (Fig. 21A–C), whereas individuals who tend to explore become particularly focused during hot streak (Fig. 21D–F). This is also validated by the difference between $\langle H \rangle$ and that of the null model (Fig. 20). For example, the difference between $\langle H \rangle$ and null model before hot streak for low-diversity artists is more pronounced than that of the high-diversity artists. Similarly, the difference between $\langle H \rangle$ and null model during hot streak for high-entropy directors is more pronounced than that of the low-entropy directors.

Supplementary Note 3.5. Regression analysis

In this section, we systematically calculate the correlation between diversity and the beginning of a hot streak by controlling individual-specific characters using OLS regression. We first study the entropy change when individuals started to explore before a hot streak begins, compared to their earlier career of the same length, after controlling for impact, career stage, and other individual characteristics:

$$\begin{aligned} H = a_0 + a_1 \times Stage_{HS} + a_2 \times N_i + a_3 \times career\ stage + a_4 \times t_i/N \\ + a_5 \times career\ age + a_6 \times \langle impact \rangle + a_7 \times \log N \end{aligned} \tag{1}$$

where $Stage_{HS} = 1$ if the work is produced before a hot streak, and 0 in earlier careers; N_i is the unique number of styles and topics within a career; N is the overall productivity; *career stage* is the relative timing of the work overall all works produced in a career; *career age* is the years since the first work, $\langle impact \rangle$ captures the average impact of all works. We further compare the regression coefficient a_1 of real careers to that of a null model, where we randomly selected a work as the beginning of a hot streak. We then repeat the analysis for diversity change after a hot streak begins, and assign $Stage_{HS} = 1$ if the work is produced during a hot streak, and 0 before it happens. Compared to the null model where a_1 is rather flat, we find that a_1 for real careers across three domains show obvious decreasing trend from before to after a hot streak begins (Fig. 22). When individuals start to explore before a hot streak, a_1 is larger than the null model would expect, and a_1 for directors and scientists are systematically larger than 0, indicating higher diversity before a hot streak begins. While after the beginning of a hot streak, a_1 becomes systematically smaller than null model, indicating a drop of diversity during exploitation. Overall, our conclusions remain the same after controlling for individual specific properties.

Supplementary Note 3.6. Scientists with two hot streaks

Do individuals with two hot streaks experience the exploration-exploitation transition in both cases? Given that the careers with multiple hot streaks are uncommon, here we only focus on scientists who have two hot streaks. We calculate $P(H)$ for papers around each hot streak (Fig. 23), finding $P(H)$ before a hot streak is systematically larger than $P(H)$ during hot streak for both hot streaks.

Supplementary Note 3.7. Scientists with hot streaks at the beginning of their career

By definition, the exploration-exploitation transition can only be measured for individuals who have produced a number of works before their hot streak begins. What about the individuals whose hot streak occurs at the beginning of their careers? To test this, we focus on scientific careers with hot streak at the beginning and compare their entropy dynamics to that of the null model (Fig. 24a), or their cohorts who do not have hot streaks at the

beginning (Fig. 24b). We find that in both cases, although we could not observe the behavior before their first record, individuals with hot streaks at the beginning have systematically smaller entropy, consistent with exploitation behavior during hot streak.

Supplementary Note 3.8. Alternative community detection method

In the main text we report the topics detected using the same community detection methods in Zeng *et al.* [18]. To test if our results are robust to different community detection algorithms, here we use Infomap [121] to detect the community structure and repeat our analysis of $P(\langle H \rangle)$, finding our conclusions remain the same (Fig. 25).

Supplementary Note 3.9. Different time window

In the main text we measure topic entropy for works produced during the hot streak and the prior period of the same length before. To test if our results are roust to entropy measured by different time window, we calculate the entropy for works produced within 5 years before and after the onset of hot streak (Fig. 26), and the same number of works produced before and after the onset of hot streak (Fig. 27), finding that our conclusions remains the same.

Supplementary Note 3.10. Alternative diversity index

To test if our results are robust to other diversity measures, here we use Simpson diversity ($D = 1 - \Sigma_i p_i^2$, where p_i is the probability of topic i), and normalize it by the maximum value. We repeat the measurement for $P(\langle Simpson \rangle)$, finding again that $\langle Simpson \rangle$ for works before a hot streak is systematically larger than the null model across the three domains (Fig. 28A–C) Similarity, $\langle Simpson \rangle$ for works after a hot streak begins is again systematically smaller than expected (Fig. 28D–F). Together, the uncovered exploration and exploitation transition remains the same for different diversity measures.

Supplementary Note 3.11. The number of styles/topics

To test the robustness of our results for the number of topics/styles m, and to compare m given different productivity levels, we calculate the number of styles/topics normalized the number of papers m/n for time periods before and during a hot streak, by controlling the productivity n over time. We compare the average $\langle m/n \rangle$ before and during hot streaks measured in real careers to the distribution of $P(\langle m/n \rangle)$ for 1000 realizations of the randomized careers across three domains (Fig. 29A-F). We further compare the distribution $P(m/n)$ before and after the hot streak begins for real and randomized careers (Fig. 29J–L). Together, Fig. 29 suggest that individuals tend to work on more topics before a hot streak and fewer topics after a hot streak begins, consistent with the shift from exploration to exploitation strategy.

We further test the robustness of our results by controlling for individuals with similar m in each domain. Specifically, we focus on individuals with m around the median value (6 to 8 for artists, 2 to 4 for directors, and 4 to 6 for scientists (Fig. 30)), and compare the average entropy measured in these careers to the distribution of 1000 realizations of the randomized careers. (Fig. 31A–F). We also directly compare the distribution of entropy before and after the hot streak begins for real and randomized careers (Fig. 31G-L). All these results show that individuals tend to work on more diverse topics before and become more focused after a hot streak begins, suggesting that the shift from exploration to exploitation strategy still hold after controlling for the number of topics m.

Supplementary Note 3.12. The faction of works on the most popular style/topic

We test the robustness of our results by measuring the fraction of works on the most popular styles/topics (Fig. 32), defined as the topic that represents the most works one produces. We find that individuals produce fewer works on the most studied topic before a hot streak, which is consistent with an exploration strategy, but they become more focused on the most popular topic during their hot streak, which is consistent with an exploitation strategy.

Supplementary Note 3.13. Switching topics

We measure the probability that an individual switches style or topic between consecutive works for periods before and during a hot streak (Fig. 33). We find that across all three domains, individuals are more likely to switch topics before hot streaks and less likely to do so during hot streaks, which are consistent with our overall conclusions.

Supplementary Note 3.14. Episodes of exploration and exploitation

To systematically understand the correlation between exploration, exploitation and the onset of career hot streaks, we define for each individual episodes of exploration and exploitation within a career by calculating the style or topic entropy in a sliding window of two years for artists and scientists, and five films for directors. We calculate the probability to initiate a hot streak at the end of an exploration episode ($P_{\downarrow}$), at the beginning of an exploitation episode ($P_{\uparrow}$), at the transition from exploration to exploitation ($P_{\downarrow\uparrow}$), and at the transition from exploitation to exploitation ($P_{\uparrow\downarrow}$), We further compare their relative change to the baseline probability P_r, defined as the average probability for the beginning of an episode to coincide with a hot streak among all episodes in a domain (0.040 for artists, 0.042 for directors, and 0.073 for scientists, respectively). In the main text (Fig. 3S-U), we report the relative change in the probability ($P = P_{\downarrow,\uparrow,\downarrow\uparrow,\uparrow\downarrow}/P_r - 1$).

Supplementary Note 3.15. Different numbers of centroids

We test in this section whether our results are robust to different number of clusters for artists and directors. We retrain the k-means clustering with 20 and 40 centroids and repeat the analysis of $P(\langle H \rangle)$, finding the results are robust with different numbers of centroids (Fig. 34).

Supplementary Note 3.16. Papers without references

We find that some of the papers do not have references in WoS, which lack sufficient information for us to identify their topics from the co-citing network. We ignore papers without references in scientific careers in the main text. To test whether our results are

robust if we include those papers, we include the impact of papers without references when we measure hot streak, use the same community detection methods to identify topics, and assign papers without references to a new topic. We then calculate the entropy distribution $P(\langle H \rangle)$ for 10 works produced before and during hot streak begins and compare to the null model, finding again that papers produced before a hot streak have higher entropy than expected, while papers during a hot streak have significantly low entropy than expected (Fig. 35).

Supplementary Note 3.17. Different style measurements

In this section, we test if the results are robust if we use different style measurements for artists and directors. We directly use style labels for artworks and genres labels for films to measure entropy distribution. We use fine-tuned VGGNet to predict the style of each image. The model output is a 29-dimensional vector with probability to each style, and the image is assigned the style with the highest probability. We then calculate the entropy distribution $P(\langle H \rangle)$ for works produced within 5 years before and during hot streak, finding our conclusion remains the same (Fig. 36). We also use genre labels in IMDB to approximate the style of each film. We focus on the genres for 5 works produced before and after a hot streak begins. If a film has multiple genres, we include all of them in the genre list. We again observe the transition from exploration to exploitation if we measure film style with genres (Fig. 37), suggesting that our analysis is robust under different style definitions.

Supplementary Note 4. SCIENTIFIC TEAMS

Motivated by the recent literature on scientific teams [120, 122], we focus on scientific careers and investigate whether there are detectable changes in collaboration patterns around the exploration-exploitation transition.

Supplementary Note 4.1. Regression analysis on team size

In the main text, we compare the team size during exploration and exploitation for all scientists in our dataset. To ensure that our results are not affected any temporal trend or

population differences, here we perform a OLS regression to study the correlation between team size and the beginning of a hot streak. We first measure the change of team size when scientists started to explore before a hot streak begins compared to their earlier career of the same length, after controlling for year, career stage and research fields:

$$\begin{aligned}\log team\ size = a_0 + a_1 \times Stage_{HS} + a_2 \times year + a_3 \times career\ stage \\ + a_4 \times career\ age + a_5 \times field\end{aligned} \tag{2}$$

where $\log team\ size$ is the logrithmic of team size for a paper, as the distribution of $P(team\ size)$ is fat-tailed in general (Fig. 38). $Stage_{HS} = 1$ if the paper is produced before a hot streak (within 10 papers), and 0 if the paper is produced in one's earlier career; *career stage* captures the relative timing of the paper overall all papers produced in a career; *career age* measures the years since the first paper; and field is the subject category in WoS which the paper belongs to. We further compare the regression coefficient a_1 of real careers to that of a null model, where we randomly selected a paper as the beginning of a hot streak. We then repeat the analysis for team size after a hot streak begins, and assign $Stage_{HS} = 1$ if the work is produced during a hot streak (within 10 papers), and 0 otherwise.

Compared to the null model where a_1 is rather flat, we find that a_1 for real careers show increasing trends from before to after the onset of a hot streak (Fig. 39). a_1 for the exploration phase is smaller than the null model would expect, and is systematically smaller than 0, indicating that scientists explore with smaller teams before a hot streak begins. While after the beginning of a hot streak, a_1 becomes systematically larger than null model, indicating that scientists work with larger teams during exploitation. Overall, our results are robust after controlling for temporal and disciplinary differences.

Supplementary Note 4.2. Different disciplines

We further split scientists into six major domains following S6.4, and run the OLS regressions for scientists in each domain separately (Fig. 40). Consistent with prior results, we find that the team size across six domains shows significant increase after a hot streak begins. a_1 for the team size before hot streak is smaller in real careers than that of the null model, and scientists from physical science, environmental science and engineering have more pronounced effects.

Supplementary Note 4.3. Team composition

The large team during hot streak may be not simply expanded from past collaborators. Indeed, a prior study [122] investigated the important role of fresh teams, prompting us to investigate whether scientists work with a new group of collaborators following the onset of hot streak. To test this, we calculate the dynamics for the number of common authors A_{shared} for 5 papers before and after a given position t, divided by the total number of unique authors A for the 10 consecutive papers (Fig. 41A). Here we focus on the individuals with similar hot streak duration ($L = 10 \pm 2$) and align their careers by the timing of the hot streak. A_{shared}/A at the beginning of a hot streak is significantly smaller than the null model, suggesting that collaborators after $t_{\uparrow}$ are less likely to overlap with collaborators before. We further validate this result by measuring the rate of new co-authors for papers. Specifically, we calculate the number of new co-authors A_{new} within a sliding window of 5 papers, divided by the number of unique authors A during the same time. We find that hot streak begins with the highest rate of new collaborators as A_{new}/A peaks at $t_{\uparrow}$, and is significantly higher than the null model (Fig. 41B). Together, Fig. 41 suggests that instead of expanding their collaborators when a hot streak begins, scientists are more likely to work with a new group of collaborators during their hot streak.

Supplementary Note 4.4. Self citations

Is the work produced during a hot streak highly cited due to the larger team size itself? One possibility is that there are more coauthors to showcase the work. This has been discussed in the team science literature, which has argued for a need to adjust for self-citations to account for the increased visibility from coauthors [123]. Following the literature, we repeated our analysis by excluding self-citations. Specifically, for each citation of a paper, we compare the coauthors' last name and first initial. If they share at least one author with the same name, we consider it as a self-citation and subtract it from C_{10}, which offers a conservative estimation on the effect of self-citations. We find that although adjusted C_{10} (without self-citation) is smaller than the raw C_{10} (lower than the diagonal line in Fig. 42A), the two values are highly correlated. We further quantify the distribution of adjusted $\log C_{10}$ for papers published during hot streaks and the rest for all scientists in the dataset, and find

that the hot streak papers have systematically higher impact (Fig. 42B), indicating that the highly cited papers are not only simply due to self-citations.

Supplementary Note 4.5. Controlling for effects of collaborations

Individuals may have varied commitments to their main topics of research and collaborative ones, suggesting that controlling for their lead-author publications may further highlight the uncovered effects. To test this, we identify, for each scientist, the authorship order in their papers. We approximate the first and last author as lead authorship. We then focus on lead-author publications during a hot streak, and measure again their entropy distribution $P(H)$. Excluding papers of a lesser role significantly reduces the topic entropy during the hot streak (Fig. 43A, KS-test p-value $= 6.1 \times 10^{-9}$). Focusing on lead-author publications alone also yields a larger difference in the entropy distribution P(H) before and during a hot streak (Fig. 43B, KS-test p-value $= 1.0 \times 10^{-55}$).

Supplementary Note 5. CHARACTERISTICS OF EXPLOITED TOPICS DURING HOT STREAK

In this section, we probe the connections between phases of exploration and exploitation. We begin by establishing how we define the exploration, exploitation and normal (typical) phases. We then examine properties of the topics that are explored before a hot streak begins, ranging from their recency to citation impact to popularity (Fig. 44), asking which ones tend to be chosen for subsequent exploitation.

Supplementary Note 5.1. Exploration, exploitation and normal phase

There are in principle three phases in a career: exploration, exploitation, and the normal phase, where the topic/style diversity is not significantly different from one's typical level. We mainly focused on the first two phases in the main text. Here we explicitly incorporate the normal phase into our framework, and test all nine combinations of the three phases and correlate them with the onset of career hot streaks. We construct a null model for each career by randomly designating one work as the start of his/her hot streak, and repeat the procedure

for 1000 realizations. This null model allows to us to identify phases of exploration, normal, and exploitation within each career by comparing the entropy in a period to the distribution $P(H)$ predicted by the null model (Fig. 45). We define exploration, exploitation, and normal phase as a period with H significantly larger, smaller, or similar to one's typical level. We measure the probability to observe the onset of hot streak for the nine different combinations between exploration, normal phase and exploitation, and compare it to a baseline when the hot streak randomly appears in a career Fig. 46). We find that the percentage change for the exploration-exploitation transition is significantly larger than zero and again the highest among all types of combinations.

Supplementary Note 5.2. Recency

Prior research shows that topic evolution along a scientific career is characterized by recency [66], implying that scientists should exploit during hot streak the latest topic they explored. We test this hypothesis by calculating the probability of the exploited topics to be the most recent before a hot streak begins (Fig. 44C, left). Among exploited topics that are studied before, around 32.7% of them are the most recent ones, lower than the null model would expect (33.6%, Chi-square test, p-value=0.0019). Thus, as individuals may learn from exploration to deliberately find a direction worth going deep, the transition from exploration to exploitation when a hot streak begins is not simply due to perceiving a new direction by chance and reaping its benefits.

Supplementary Note 5.3. Popularity in a career

Prior study also shows that scientists have their core research topic that are repeatedly investigated [66], suggesting the exploited topic may be popular within a career. To test this, we measure the popularity of a topic by the number of paper published before the onset of hot streak, and calculate the probability for the topic exploited to be the most popular. We find that the probability to select the most popular topic is lower than expected (Chi-square test, p-value=6.12×10^{-40}), suggesting that scientists are less likely to continue their past focal topic during hot streak.

Supplementary Note 5.4. Impact

Is the exploited topic the highest cited among all topics explored before? We compare the impact for topics before a hot streak by measuring the average paper citation till the onset of hot streak, and categorize explored topics into low, middle and high impact. We calculate the probability for the exploited topic to fall into each category, and find that the topics studied during exploitation are less likely to be the high-impact topic (Fig. 44D).

Supplementary Note 5.5. Popularity in embedding space

Exploration may increase the likelihood for individuals to stumble upon a hot topic and reap its benefits during hot streak, prompting us to test if the exploited topic is popular at that time. We project the 64-dimensional embeddings learned from co-citing network among all papers in the dataset onto two dimensions using PCA. We calculate the density on these dimensions by measuring the volume of papers at the time in the embedding space. The popularity of a topic is defined as the average density for papers belonging to the topic when they were published. We find that the topics studied during exploitation are less likely to reside in either high- or low- density regions but rather somewhere in the middle (Fig. 44E). We also calculate the momentum of each topic during the exploration phase, which traces the rate of increase in popularity within five years before the onset of hot streak, finding again that the topic exploited is not among the fastest growing (Fig. 44E, inset)

Supplementary Note 5.6. Predicting topic exploited

We utilize the topic properties discussed above together with the team size to predict which topic a scientist will choose to exploit during their hot streak. Specifically, we formulate a binary classification problem: For each scientist, given each topic she explored before hot streak, we predict whether it will be exploited during the hot streak based on its recency, impact, popularity and team size. Here we focus on careers whose exploited topics were among those studied before (around 80% among all scientists in the dataset). We calculate the topic recency, impact, popularity in a career and in the embedding space following th same procedure above. The team size of each topic is calculated as the average log team size for papers belonging to the topic that were published before hot streak.

We randomly sample 80% of topic records as training set and the rest 20% as the test set, and use the random forest model with 500 trees to train the classifier. The model accuracy is 0.89 and AUC is 0.83 (Fig. 44F). We also test the effect of imbalanced positive (exploited) and negative (not exploited) sample size, and down-sample the negative cases to the same amount of positive ones, finding the AUC remain above 0.8. We further compare the model accuracy to two baselines: 1) the accuracy for the topic from the last paper before hot streak is 0.64; 2) the accuracy for a randomly selected topic before hot streak is 0.43, finding our prediction model significantly outperforms the two baselines.

Supplementary Note 6. TESTING ALTERNATIVE HYPOTHESIS

To explore alternative explanations for career hot streaks, we test several hypotheses in this section, each capturing plausible factors or processes for career progression and success.

Supplementary Note 6.1. Multiple publications

. Innovators may stumble upon a groundbreaking idea, which manifests itself in the forms of multiple artworks, films, or publications. Hence from an evolutionary perspective, hot streak may correspond to the duration for the temporary competitive advantage to dissipate. We test this hypothesis by measuring the relative order of three highest impact papers during hot streak. Multiple publications hypothesis predicts that the highest impact paper should be more likely to occur before the second highest. By contrast, we find that there is an equal probability for the highest-impact work to appear before or after the second highest (Fig. 47A).

Supplementary Note 6.2. New research direction

Some research topics are more impactful than others, and hot streaks may be simply driven by switching to a new research direction, which affects an individual's overall achievements. We test this hypothesis by measuring the probability of observing a new topic when hot streak begins, and compare it with that of the randomized careers (Fig. 47B). However,

we find no detectable difference between data and the null model (Chi-square test, p-value =0.23).

Supplementary Note 6.3. Collaboration with high-impact scientists

Teams are increasingly responsible for producing high-impact work, suggesting hot streak may reflect fruitful, repeated collaborations with other high-impact individuals. To test this, we quantify the reputation of one's coauthors using h-index, and measure the h-index distribution for the most prestigious co-author of each paper published during hot streak. We find that the h-index for the most prestigious coauthor is largely indistinguishable from the null model (Fig. 47C).

Supplementary Note 6.4. Changing institutions

Scientists moving to a new intellectual environment may be exposed to new sets of ideas or opportunities, which may increase their likelihood to produce high-impact work . To investigate the relationship between changing institutions and hot streak, we trace the physical mobility of scientists through their affiliations recorded in publication records [124], and calculate the probability of changing institutions during their hot streak. We find that hot streaks are less likely to be associated with affiliation change than expected (Chi-square test, p-value $= 1.8 \times 10^{-41}$, Fig. 47D).

Supplementary Note 6.5. Research support

A new grant may help accelerate a scientist's research progress and offer opportunities to produce high-impact papers. To test whether hot streak can be explained by new grants, we linked the careers of scientists in our dataset with a funding database that captures 3.7 million funded projects across more than 250 funding agencies worldwide (https://www.dimensions.ai/) by the same last name, first initial and affiliation. We measure the number of new grants around the onset of a hot streak, and find no detectable differences compared with the null model (Fig. S39E, Chi-square test, p-value = 0.36). We further calculate the amount of funding one received around the beginning of a hot streak,

finding again a lack of difference between data and the null model (Fig. 47E, KS test, p-value = 0.67).

Supplementary Note 6.6. Future hot topic

Although scientists are less likely to work on the most popular topic at the time, scientists may happen to work on a topic that becomes hot in the future. To test this hypothesis, we quantify the impact of each topic in the WoS data and compare the distribution of topic impact during hot streak to that of before, and find that the impact improvement for topics appears negligible relative to the overall impact change in real careers (Fig. 47F).

Supplementary Note 6.7. Exploiting previously successful work

. Next, we analyze whether the doubling down on prior success might be a potential explanation for the onset of hot streaks (i.e. if something is successful, do it again). The Matthew effect suggests that an individual's initial success may bring resources and reputation which may help the individual to succeed again in the future. From this perspective, creating subsequent work that is similar to one's successful prior work may be advantageous. One key prediction of the Matthew effect is first mover advantage [125, 126]. It thus suggests that we should expect that on average individual performance would decrease over time as one repeats the same recipe for success. Indeed, research shows that while sequel films have good box-office performance in general, it is rare for sequels to outperform the predecessors in terms of either gross box office or the probability to earn award recognitions [127, 128]. Similarly, in science, the first study that opens up a new line of inquiry tends to be highly cited. And follow-up studies that continue the investigations may also attract attention, but are less likely to be cited at the same level as the canonical paper.

We perform two different measurements to test this prediction: (1) We compare the impact distribution of works produced in the first and second half of the hot streak, as measured by the logarithmic of auction price, the IMDB rating and the logarithmic paper citations in 10 years C_{10} (Fig. 48A-C). (2) We measure the relative position of the highest-impact work among the top six highest-impact works in a career (Fig. 48D-F). Interestingly—and

contrary to what the hypothesis would predict—we find no systematic difference between the impact in the first and second half of the hot streak. Moreover, we observe an equal probability for the highest-impact work to appear before and after other hits in a career. In sum, our results show that the impact during a hot streak stays remarkably stable, and does not exhibit decreasing trends in impact.

Supplementary Note 6.8. An autoregressive process

An autoregressive model assumes short-range correlations in the impact sequence and combines with a random term to predict one's future impact. Specifically, we can express an autoregressive model $AR(\rho)$ for the impact sequence X_t of each individual career as $X_t = c + \Sigma_{i=1}^{\rho} \beta_i X_{t-1} + \epsilon$, where ρ is the number of preceding time steps, β_i is the correlation at lag i, c is a constant, and ϵ is white noise.

At the first glance, the autoregressive model appears a rather plausible candidate for describing the hot streak dynamics, given that high-impact works are temporarily clustered in careers. To test its validity, we fit $AR(1)$ to our data and test whether the model can reproduce the clustering of hits in real careers. We measure the correlations between the relative timing of an individual's two biggest hits N^* and N^{**} predicted by the best fitted parameters of each career (Fig. 49A-C). In contrast to patterns in real careers (Fig. 49A-F), we find that there is little correlation between the timing of N^* and N^{**} under the $AR(1)$ model (Fig. 49D-F). We also calculate the distribution of streak length $P(L)$, as predicted by $AR(1)$, defined as the number of consecutive works whose impacts exceed the median impact of all works within a career, compared with a null model where the impact sequence is randomly shuffled (Fig. 49J-O)). We find that the model fails to capture the fact that high-impact works tend to be clustered in sequence in real careers. We further relax autoregressive model to allow for larger lags and test the predictions by $AR(5)$, finding again the model fails to reproduce the clustering of high-impact works in real careers (Fig. 49G-I, P-R).

Overall, these results indicate that while the hot streak dynamics imply a temporal correlation, simply having the temporal correlation by itself is insufficient to reproduce the dynamics observed in real careers. The main reason is that the correlations vary over time,

according to the hot streak dynamics which accounts for the period of sustained high performance. Indeed, overall, the level of correlation across a whole career is rather mild. When we measure directly the autocorrelation function for real careers across the three domains, we find that the lag 1 autocorrelation is 0.04 for artists and movie directors, and 0.05 for scientists. The correlation becomes even smaller for larger lags. These results indicate that the assumptions underlying the hot streak model remain the key to reproducing the patterns we observe in real careers; without these assumptions, a generic autoregressive model by itself cannot account for the observed patterns.

[1] L. Liu, *et al.*, *Nature* **559**, 396 (2018).

[2] H. Mao, M. Cheung, J. She, *Proceedings of the 25th ACM international conference on Multimedia* (ACM, 2017), pp. 1183–1191.

[3] H. Mao, J. She, M. Cheung, *ACM Transactions on Multimedia Computing, Communications, and Applications (TOMM)* **15**, 60 (2019).

[4] D. W. Galenson, *Old masters and young geniuses: The two life cycles of artistic creativity* (Princeton University Press, 2011).

[5] D. W. Galenson, B. A. Weinberg, *Journal of Political Economy* **108**, 761 (2000).

[6] A. Elgammal, B. Liu, D. Kim, M. Elhoseiny, M. Mazzone, *Thirty-Second AAAI Conference on Artificial Intelligence* (2018).

[7] K. Simonyan, A. Zisserman, *arXiv preprint arXiv:1409.1556* (2014).

[8] L. A. Gatys, A. S. Ecker, M. Bethge, *Proceedings of the IEEE conference on computer vision and pattern recognition* (2016), pp. 2414–2423.

[9] M. Wasserman, X. H. T. Zeng, L. A. N. Amaral, *Proceedings of the National Academy of Sciences* **112**, 1281 (2015).

[10] Q. Hoang, *arXiv preprint arXiv:1801.04813* (2018).

[11] S. Kar, S. Maharjan, A. P. López-Monroy, T. Solorio, *arXiv preprint arXiv:1802.07858* (2018).

[12] K.-W. Ho, Movies' genres classification by synopsis (2011).

[13] W. T. Wallace, A. Seigerman, M. B. Holbrook, *Journal of cultural economics* pp. 1–27 (1993).

[14] M. H. Kim, *Applied Economics Letters* **20**, 1071 (2013).

[15] B. Perozzi, R. Al-Rfou, S. Skiena, *Proceedings of the 20th ACM SIGKDD international conference on Knowledge discovery and data mining* (2014), pp. 701–710.

[16] R. Sinatra, D. Wang, P. Deville, C. Song, A.-L. Barabási, *Science* **354**, aaf5239 (2016).

[17] F. Radicchi, C. Castellano, *Scientometrics* **97**, 627 (2013).

[18] A. Zeng, *et al.*, *Nature communications* **10**, 1 (2019).

[19] F. Radicchi, S. Fortunato, C. Castellano, *Proceedings of the National Academy of Sciences* **105**, 17268 (2008).

[20] M. J. Stringer, M. Sales-Pardo, L. A. N. Amaral, *Plos one* **3**, e1683 (2008).

[21] M. Perc, *Journal of the Royal Society Interface* **17**, 20190686 (2020).

[22] R. P. Taylor, A. P. Micolich, D. Jonas, *Nature* **399**, 422 (1999).

[23] J. Alvarez-Ramirez, C. Ibarra-Valdez, E. Rodriguez, *Chaos, Solitons & Fractals* **83**, 97 (2016).

[24] P. Pedram, G. Jafari, *International Journal of Modern Physics C* **19**, 855 (2008).

[25] M. Koch, J. Denzler, C. Redies, *PLoS one* **5** (2010).

[26] L. Shamir, *Leonardo* **45**, 149 (2012).

[27] C. Montagner, J. M. Linhares, M. Vilarigues, S. M. Nascimento, *JOSA A* **33**, A170 (2016).

[28] B. Lee, D. Kim, S. Sun, H. Jeong, J. Park, *arXiv preprint arXiv:1701.07164* (2017).

[29] D. Kim, S.-W. Son, H. Jeong, *Scientific reports* **4**, 7370 (2014).

[30] H. Y. Sigaki, M. Perc, H. V. Ribeiro, *Proceedings of the National Academy of Sciences* **115**, E8585 (2018).

[31] L. Chen, J. Yang, *Proceedings of the 27th ACM International Conference on Multimedia* (2019), pp. 2459–2467.

[32] Y. Bar, N. Levy, L. Wolf, *European conference on computer vision* (Springer, 2014), pp. 71–84.

[33] W.-T. Chu, Y.-L. Wu, *Proceedings of the 24th ACM international conference on Multimedia* (2016), pp. 402–406.

[34] A. Lecoutre, B. Negrevergne, F. Yger, *Asian conference on machine learning* (2017), pp. 327–342.

[35] D. Kim, B. Liu, A. Elgammal, M. Mazzone, *2018 IEEE 12th International Conference on Semantic Computing (ICSC)* (IEEE, 2018), pp. 156–163.

[36] G. Strezoski, M. Worring, *ACM Transactions on Multimedia Computing, Communications, and Applications (TOMM)* **14**, 1 (2018).

[37] K. He, X. Zhang, S. Ren, J. Sun, *Proceedings of the IEEE conference on computer vision and pattern recognition* (2016), pp. 770–778.

[38] A. Krizhevsky, I. Sutskever, G. E. Hinton, *Advances in neural information processing systems* (2012), pp. 1097–1105.

[39] S. Karayev, *et al.*, *arXiv preprint arXiv:1311.3715* (2013).

[40] Y. Jing, *et al.*, *IEEE transactions on visualization and computer graphics* (2019).

[41] J. Johnson, A. Alahi, L. Fei-Fei, *European conference on computer vision* (Springer, 2016),

pp. 694–711.

[42] X. Huang, S. Belongie, *Proceedings of the IEEE International Conference on Computer Vision* (2017), pp. 1501–1510.

[43] P. Isola, J.-Y. Zhu, T. Zhou, A. A. Efros, *Proceedings of the IEEE conference on computer vision and pattern recognition* (2017), pp. 1125–1134.

[44] A. Sanakoyeu, D. Kotovenko, S. Lang, B. Ommer, *Proceedings of the European Conference on Computer Vision (ECCV)* (2018), pp. 698–714.

[45] J.-Y. Zhu, T. Park, P. Isola, A. A. Efros, *Proceedings of the IEEE international conference on computer vision* (2017), pp. 2223–2232.

[46] A. Elgammal, B. Liu, M. Elhoseiny, M. Mazzone, *arXiv preprint arXiv:1706.07068* (2017).

[47] R. S. Sutton, A. G. Barto, *Reinforcement learning: An introduction* (MIT press, 2018).

[48] J.-Y. Audibert, R. Munos, C. Szepesvári, *Theoretical Computer Science* **410**, 1876 (2009).

[49] T. Desautels, A. Krause, J. W. Burdick, *Journal of Machine Learning Research* **15**, 3873 (2014).

[50] K. Mehlhorn, *et al.*, *Decision* **2**, 191 (2015).

[51] J. D. Cohen, S. M. McClure, A. J. Yu, *Philosophical Transactions of the Royal Society B: Biological Sciences* **362**, 933 (2007).

[52] T. T. Hills, *et al.*, *Trends in cognitive sciences* **19**, 46 (2015).

[53] M. D. Humphries, M. Khamassi, K. Gurney, *Frontiers in neuroscience* **6**, 9 (2012).

[54] A. Aleta, S. Meloni, N. Perra, Y. Moreno, *EPJ Data Science* **8**, 1 (2019).

[55] J. G. Foster, A. Rzhetsky, J. A. Evans, *American Sociological Review* **80**, 875 (2015).

[56] O. Berger-Tal, J. Nathan, E. Meron, D. Saltz, *PloS one* **9** (2014).

[57] R. Henderson, I. Cockburn, *Strategic management journal* **15**, 63 (1994).

[58] L. Rosenkopf, A. Nerkar, *Strategic management journal* **22**, 287 (2001).

[59] J. G. March, *Organization science* **2**, 71 (1991).

[60] D. Lavie, U. Stettner, M. L. Tushman, *The Academy of Management Annals* **4**, 109 (2010).

[61] H. E. Posen, D. A. Levinthal, *Management Science* **58**, 587 (2012).

[62] T. S. Kuhn, J. Epstein, The essential tension (1979).

[63] P. Bourdieu, *Information (International Social Science Council)* **14**, 19 (1975).

[64] M. Polanyi, J. Ziman, S. Fuller, *Minerva* **38**, 1 (2000).

[65] A. Rzhetsky, J. G. Foster, I. T. Foster, J. A. Evans, *Proceedings of the National Academy of*

Sciences **112**, 14569 (2015).

[66] T. Jia, D. Wang, B. K. Szymanski, *Nature Human Behaviour* **1**, 1 (2017).

[67] E. L. Charnov, *et al.* (1976).

[68] R. Hau, T. J. Pleskac, J. Kiefer, R. Hertwig, *Journal of Behavioral Decision Making* **21**, 493 (2008).

[69] T. Rakow, B. R. Newell, *Journal of Behavioral Decision Making* **23**, 1 (2010).

[70] R. L. Kendal, *et al.*, *Trends in cognitive sciences* **22**, 651 (2018).

[71] L. Rendell, *et al.*, *Trends in cognitive sciences* **15**, 68 (2011).

[72] W. Toyokawa, H.-r. Kim, T. Kameda, *PloS one* **9**, e95789 (2014).

[73] P. M. Krafft, R. X. Hawkins, A. Pentland, N. D. Goodman, J. B. Tenenbaum, *CogSci* (2015).

[74] R. L. Goldstone, M. E. Roberts, T. M. Gureckis, *Current Directions in Psychological Science* **17**, 10 (2008).

[75] S. Billinger, N. Stieglitz, T. R. Schumacher, *Organization Science* **25**, 93 (2014).

[76] L. Fleming, S. Mingo, D. Chen, *Administrative science quarterly* **52**, 443 (2007).

[77] D. Lazer, A. Friedman, *Administrative science quarterly* **52**, 667 (2007).

[78] T. S. Bateman, A. M. Hess, *Proceedings of the National Academy of Sciences* **112**, 3653 (2015).

[79] T. T. Hills, T. Pachur, *Journal of Experimental Psychology: Learning, Memory, and Cognition* **38**, 218 (2012).

[80] A. M. Parker, W. B. De Bruin, B. Fischhoff, *Judgment and Decision making* **2**, 342 (2007).

[81] J. Singh, A. Agrawal, *Management Science* **57**, 129 (2011).

[82] B. Groysberg, L.-E. Lee, *Organization science* **20**, 740 (2009).

[83] S. Lee, P. Meyer-Doyle, *Organization Science* **28**, 19 (2017).

[84] R. S. Burt, J. Merluzzi, *Academy of Management Discoveries* **2**, 368 (2016).

[85] M. L. Tushman, C. A. O'Reilly III, *California management review* **38**, 8 (1996).

[86] M. Holmqvist, *Organization science* **15**, 70 (2004).

[87] J. B. Sørensen, T. E. Stuart, *Administrative science quarterly* **45**, 81 (2000).

[88] J. Luger, S. Raisch, M. Schimmer, *Organization Science* **29**, 449 (2018).

[89] M. Piao, E. J. Zajac, *Strategic Management Journal* **37**, 1431 (2016).

[90] J. J. Jansen, F. A. Van den Bosch, H. W. Volberda, *Schmalenbach Business Review* **57**, 351 (2005).

[91] A. K. Gupta, K. G. Smith, C. E. Shalley, *Academy of management journal* **49**, 693 (2006).

[92] S. Auh, B. Menguc, *Journal of business research* **58**, 1652 (2005).

[93] F. A. Csaszar, J. Eggers, *Management Science* **59**, 2257 (2013).

[94] N. Siggelkow, J. W. Rivkin, *Academy of Management Journal* **49**, 779 (2006).

[95] T. Knudsen, K. Srikanth, *Administrative Science Quarterly* **59**, 409 (2014).

[96] D. Lavie, L. Rosenkopf, *Academy of management journal* **49**, 797 (2006).

[97] F. T. Rothaermel, D. L. Deeds, *Strategic management journal* **25**, 201 (2004).

[98] T. Swift, *Strategic Management Journal* **37**, 1688 (2016).

[99] M. Zollo, S. G. Winter, *Organization science* **13**, 339 (2002).

[100] I. Iacopini, S. Milojević, V. Latora, *Physical review letters* **120**, 048301 (2018).

[101] V. Loreto, V. D. Servedio, S. H. Strogatz, F. Tria, *Creativity and universality in language* (Springer, 2016), pp. 59–83.

[102] F. Tria, V. Loreto, V. D. P. Servedio, S. H. Strogatz, *Scientific reports* **4**, 5890 (2014).

[103] B. Uzzi, S. Mukherjee, M. Stringer, B. Jones, *Science* **342**, 468 (2013).

[104] H. Youn, D. Strumsky, L. M. Bettencourt, J. Lobo, *Journal of The Royal Society Interface* **12**, 20150272 (2015).

[105] J. Kang, S.-J. Kim, *Strategic Management Journal* **41**, 1083 (2020).

[106] S. Shibayama, *Research Policy* **48**, 676 (2019).

[107] R. De Langhe, *Historical Social Research/Historische Sozialforschung* **43**, 28 (2018).

[108] J. Chin, E. Anderson, C.-L. Chin, W.-T. Fu, *Proceedings of the Human Factors and Ergonomics Society annual meeting* (SAGE Publications Sage CA: Los Angeles, CA, 2015), vol. 59, pp. 85–89.

[109] R. De Langhe, P. Rubbens, *Kuhn's Structure of Scientific Revolutions-50 Years On* (Springer, 2015), pp. 105–114.

[110] B. R. Spisak, A. E. Grabo, R. D. Arvey, M. van Vugt, *The Leadership Quarterly* **25**, 805 (2014).

[111] F.-J. Molina-Castillo, D. Jimenez-Jimenez, J.-L. Munuera-Aleman, *Industrial Marketing Management* **40**, 1172 (2011).

[112] C. Fang, J. Lee, M. A. Schilling, *Organization Science* **21**, 625 (2010).

[113] M. Weisberg, R. Muldoon, *Philosophy of science* **76**, 225 (2009).

[114] P. Mc Namara, C. Baden-Fuller, *Research Policy* **36**, 548 (2007).

[115] S. Eliassen, C. Jørgensen, M. Mangel, J. Giske, *Oikos* **116**, 513 (2007).

[116] J. S. Sidhu, H. R. Commandeur, H. W. Volberda, *Organization Science* **18**, 20 (2007).

[117] Z.-L. He, P.-K. Wong, *Organization science* **15**, 481 (2004).

[118] R. A. Burgelman, *Administrative science quarterly* **47**, 325 (2002).

[119] D. A. Levinthal, J. G. March, *Strategic management journal* **14**, 95 (1993).

[120] L. Wu, D. Wang, J. A. Evans, *Nature* **566**, 378 (2019).

[121] M. Rosvall, C. T. Bergstrom, *Proceedings of the National Academy of Sciences* **105**, 1118 (2008).

[122] A. Zeng, Y. Fan, Z. Di, Y. Wang, S. Havlin, *Nature Human Behaviour* pp. 1–9 (2021).

[123] J. M. Valderas, *Science* **317**, 1496 (2007).

[124] P. Deville, *et al.*, *Scientific reports* **4**, 4770 (2014).

[125] R. K. Merton, *Science* **159**, 56 (1968).

[126] A.-L. Barabási, R. Albert, *science* **286**, 509 (1999).

[127] D. K. Simonton, *Psychology & marketing* **26**, 400 (2009).

[128] G. Cattani, S. Ferriani, *Organization science* **19**, 824 (2008).

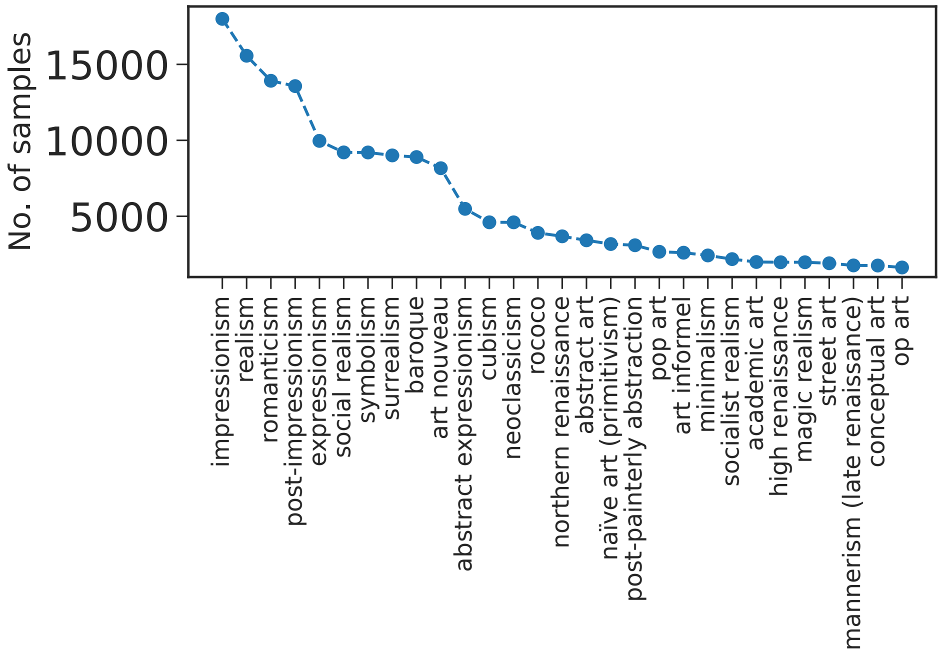

Supplymentary Figure 1: The number of samples for top 20 labels in Art500k

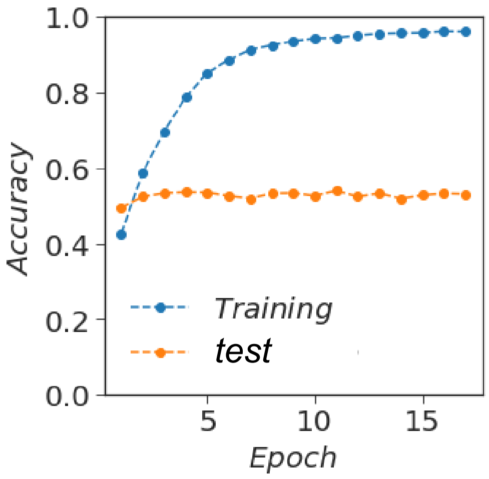

Supplymentary Figure 2: The training and test accuracy of fine-tuned VGG16 to predict art style labels.

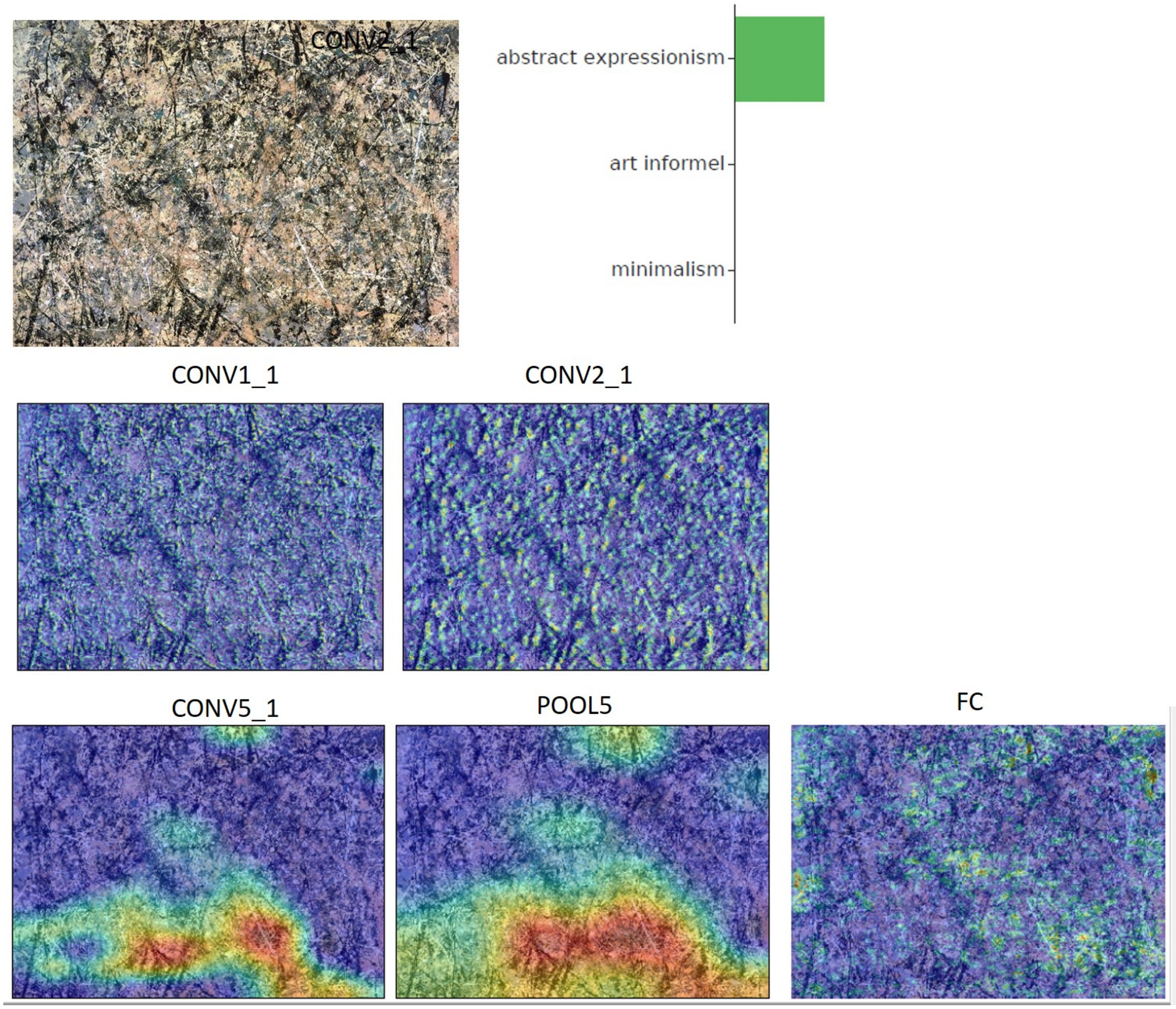

Supplymentary Figure 3: A case study on model interpretation for Number 1. The top row shows the raw image and the model prediction results, with probability in top 3 styles. The middle and low panel show a random feature map from $CONV1\_1$, $CONV2\_1$, $CONV5\_1$, $POOL5$ and the saliency map with the most likely art style (FC). Image reproduced under Creative Commons Attribution-Share Alike 4.0 International license.

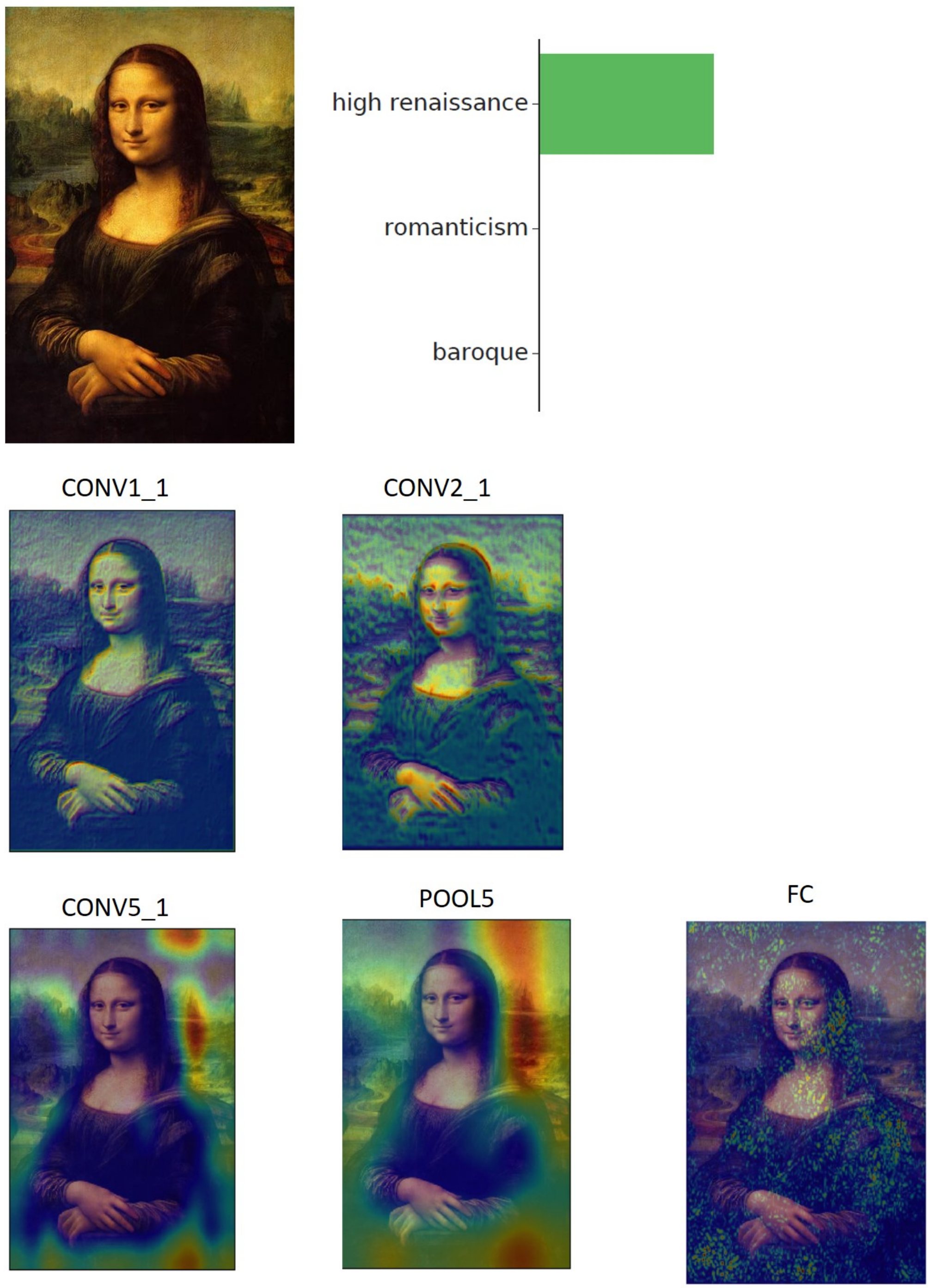

Supplymentary Figure 4: A case study on model interpretation for Mona Lisa. The top row shows the raw image and the model prediction results, with probability in top 3 styles. The middle and low panel show a random feature map from $CONV1\_1$, $CONV2\_1$, $CONV5\_1$, $POOL5$ and the saliency map with the most likely art style (FC).

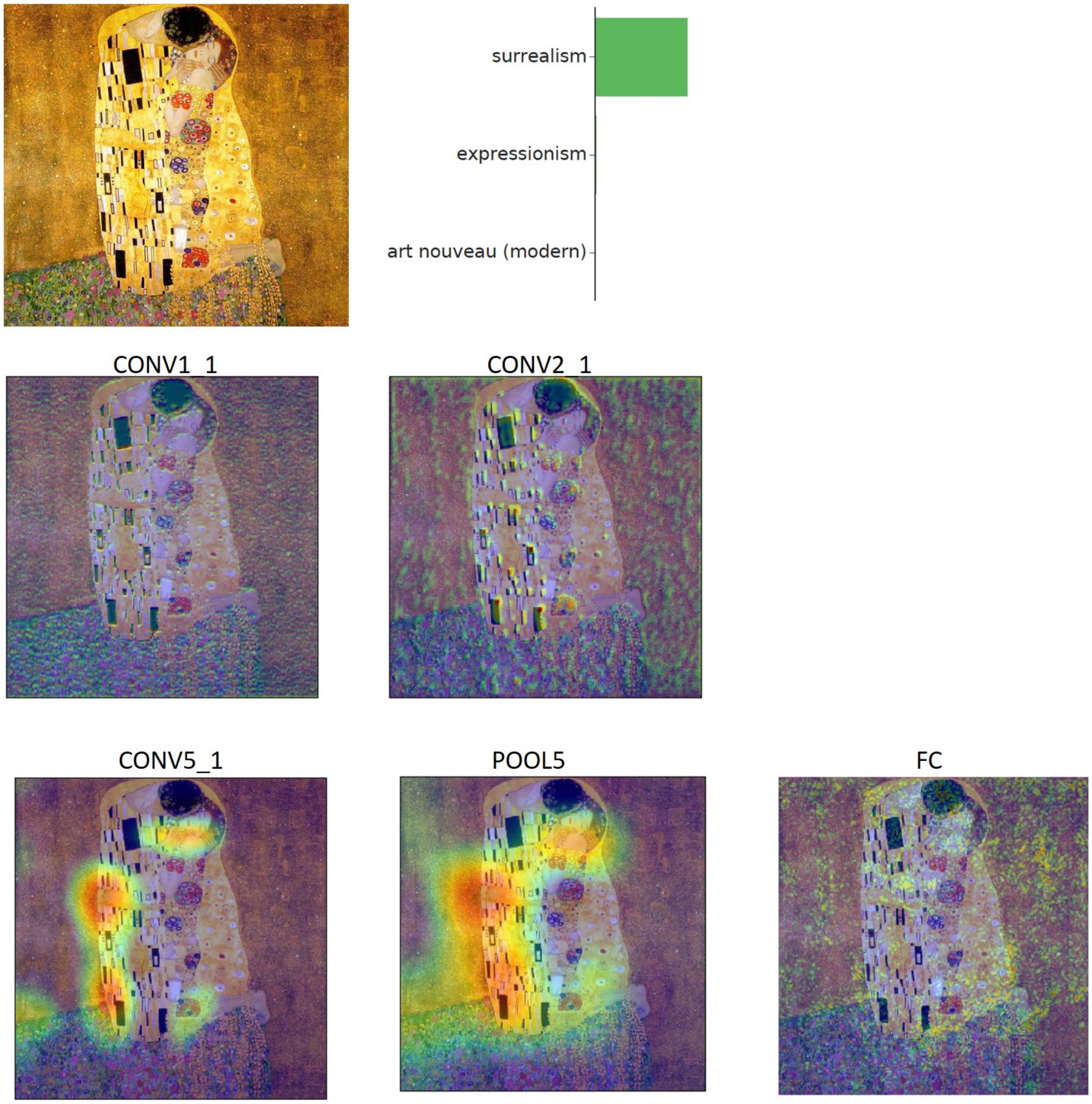

Supplymentary Figure 5: A case study on model interpretation for Kiss. The top row shows the raw image and the model prediction results, with probability in top 3 styles. The middle and low panel show a random feature map from $CONV1\_1$, $CONV2\_1$, $CONV5\_1$, $POOL5$ and the saliency map with the most likely art style (FC).

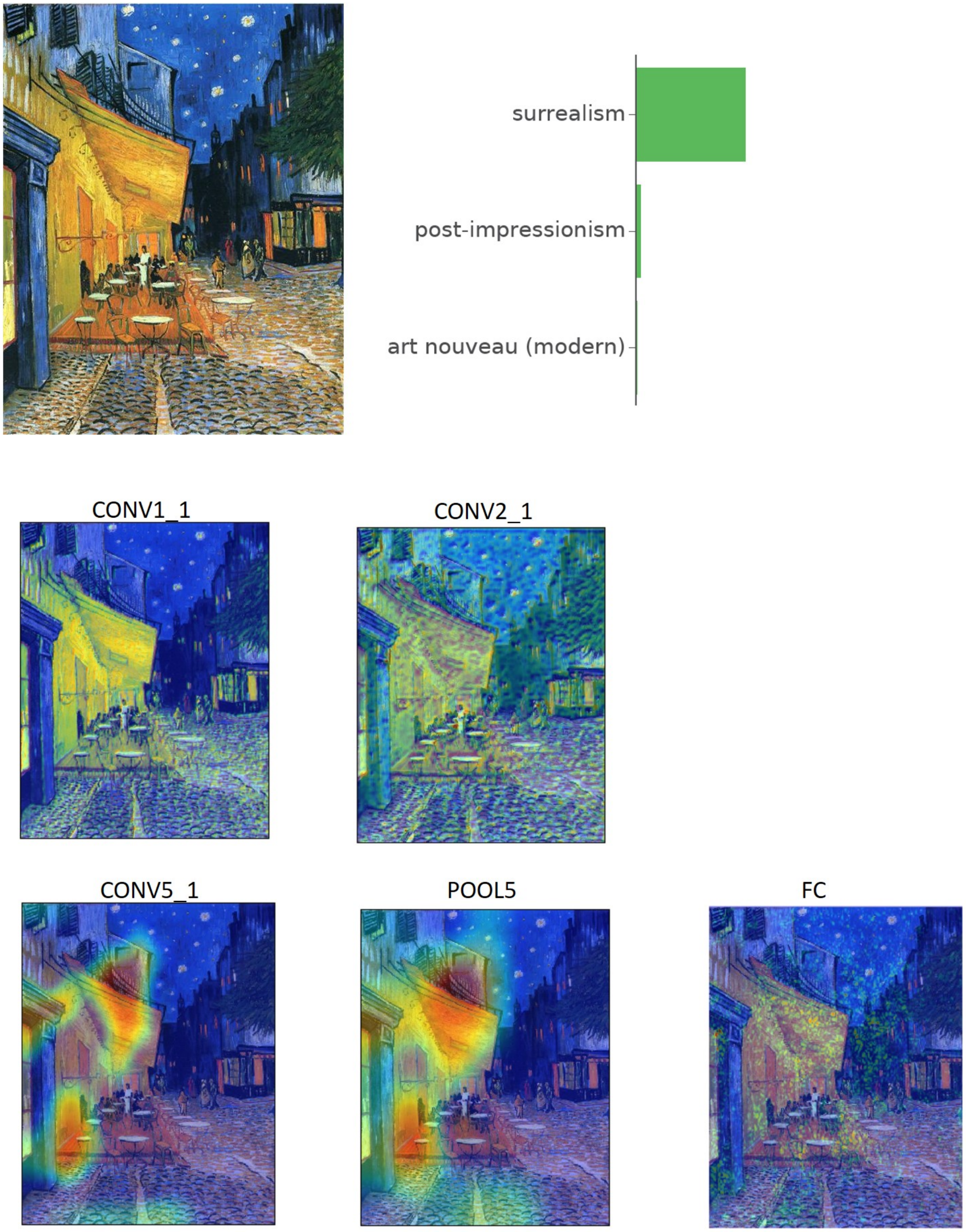

Supplymentary Figure 6: A case study on model interpretation for Cafe terrace. The top row shows the raw image and the model prediction results, with probability in top 3 styles. The middle and low panel show a random feature map from $CONV1\_1$, $CONV2\_1$, $CONV5\_1$, $POOL5$ and the saliency map with the most likely art style (FC).

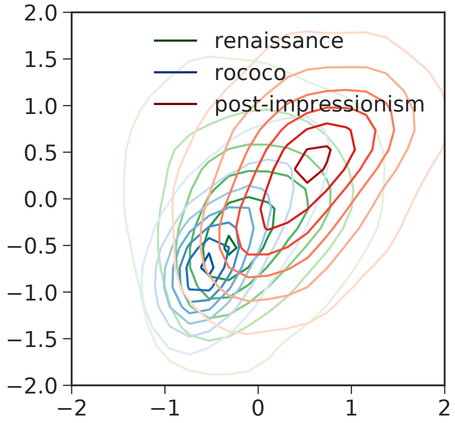

Supplymentary Figure 7: The kernel density for images from renaissance, impressionism and pop art projected onto a 2D embedding space with principle component analysis

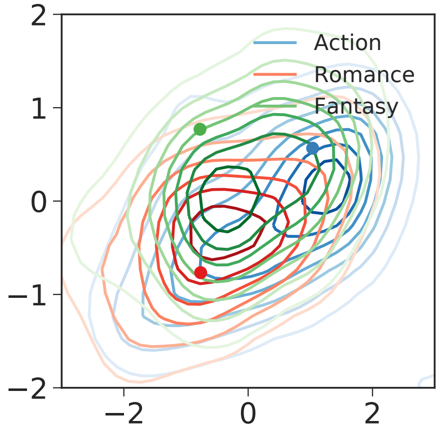

Supplymentary Figure 8: The kernel density for action, romance and fantasy films projected onto a 2D embedding space with principle component analysis

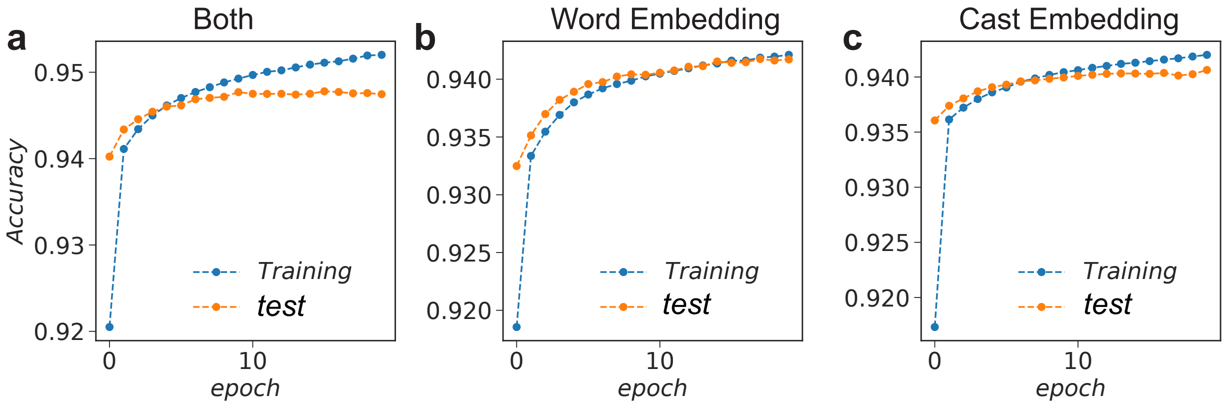

Supplymentary Figure 9: The training and test accuracy for the neural network to predict film genres.

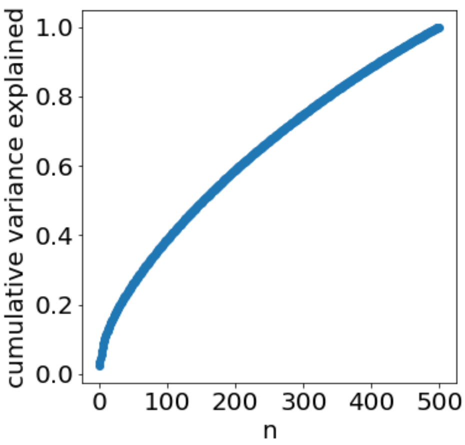

Supplymentary Figure 10: The cumulative variance explained for the top 100 eigenvalues.

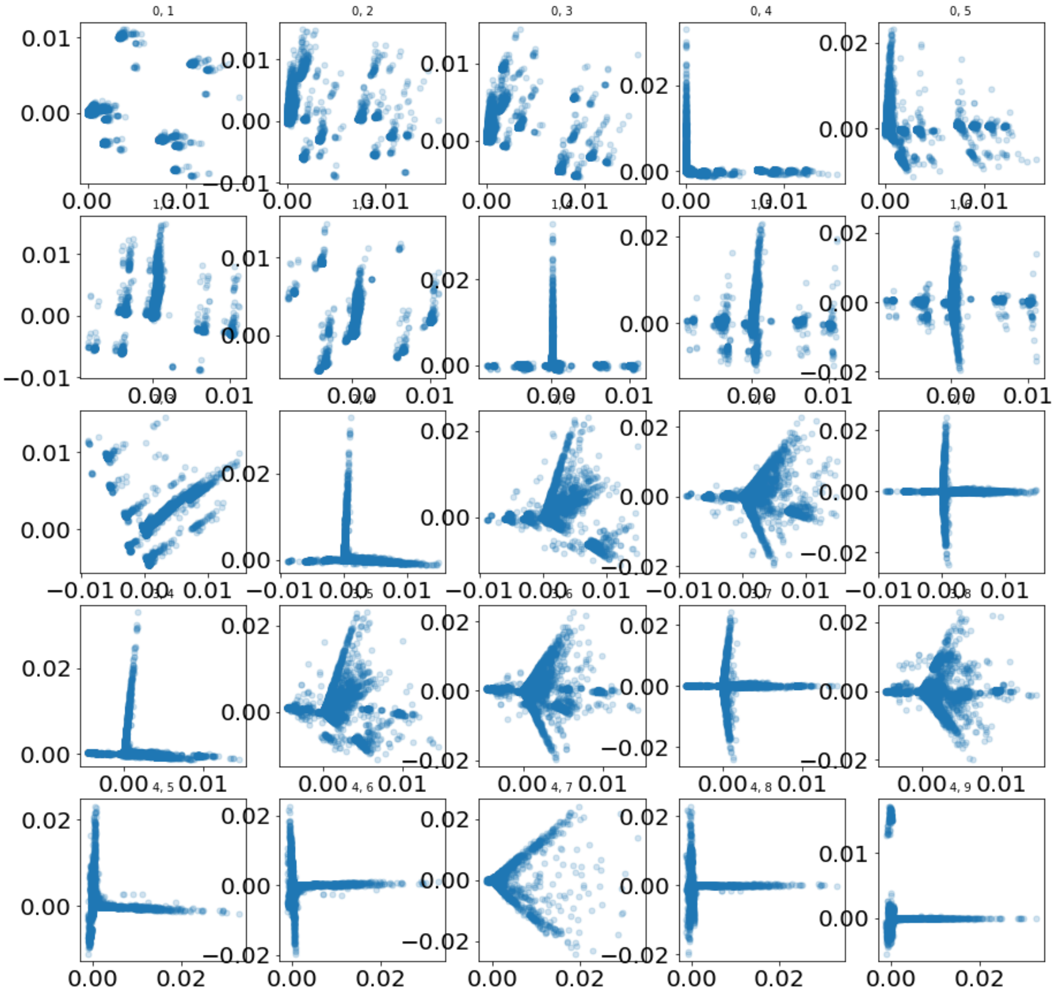

Supplymentary Figure 11: The scatter plot for two columns from the u matrix, only considered top 100k samples. The title in each subplot represents the dimension we measured.

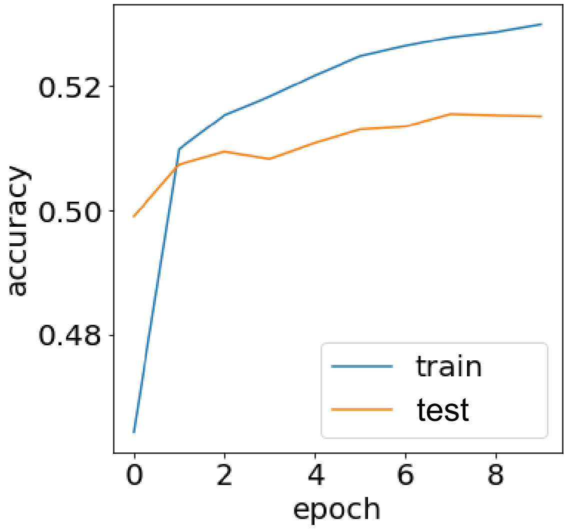

Supplymentary Figure 12: The training and test accuracy for the neural network in predicting paper subject.

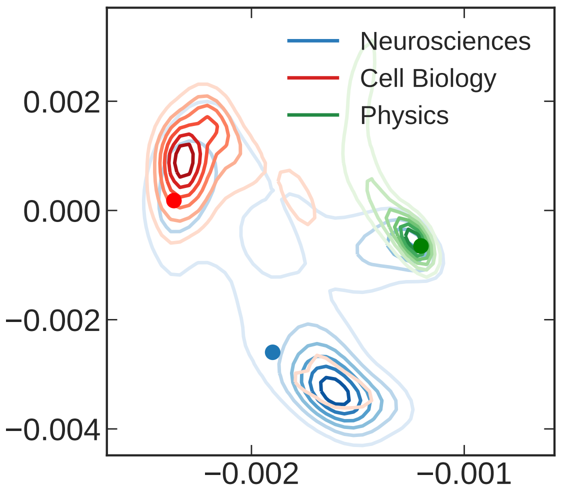

Supplymentary Figure 13: The kernel density for neurosciences, cell biology and physics papers projected onto a 2D embedding space with principle component analysis.

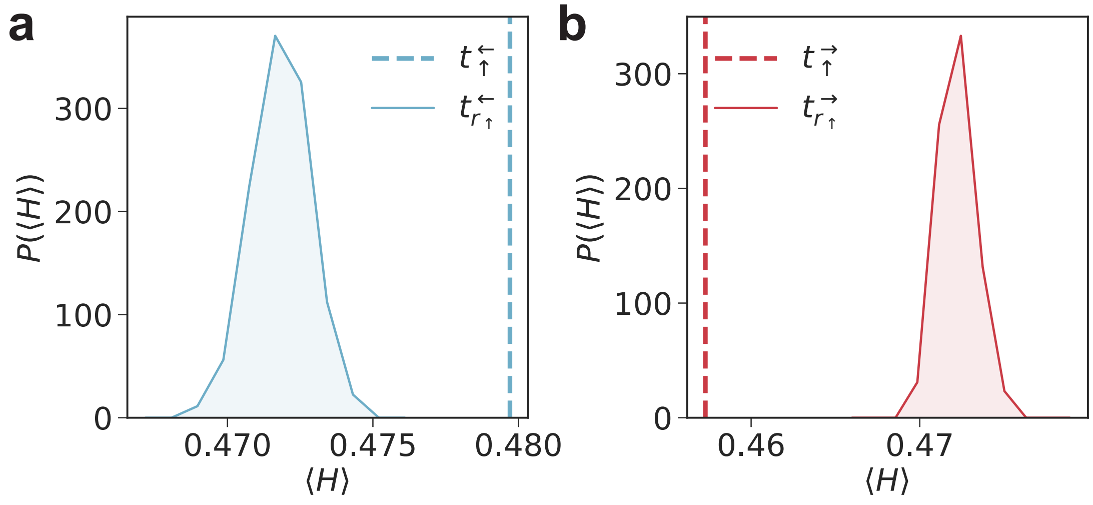

Supplymentary Figure 14: The distribution of rescaled entropy $P(\langle H \rangle)$ before and during hot streak for 1000 realizations of the randomized scientific careers using topics measured from the node embedding space. $\langle H \rangle$ measured from real careers (vertical line) is significantly larger than expected before hot streak and smaller than expected during hot streak.

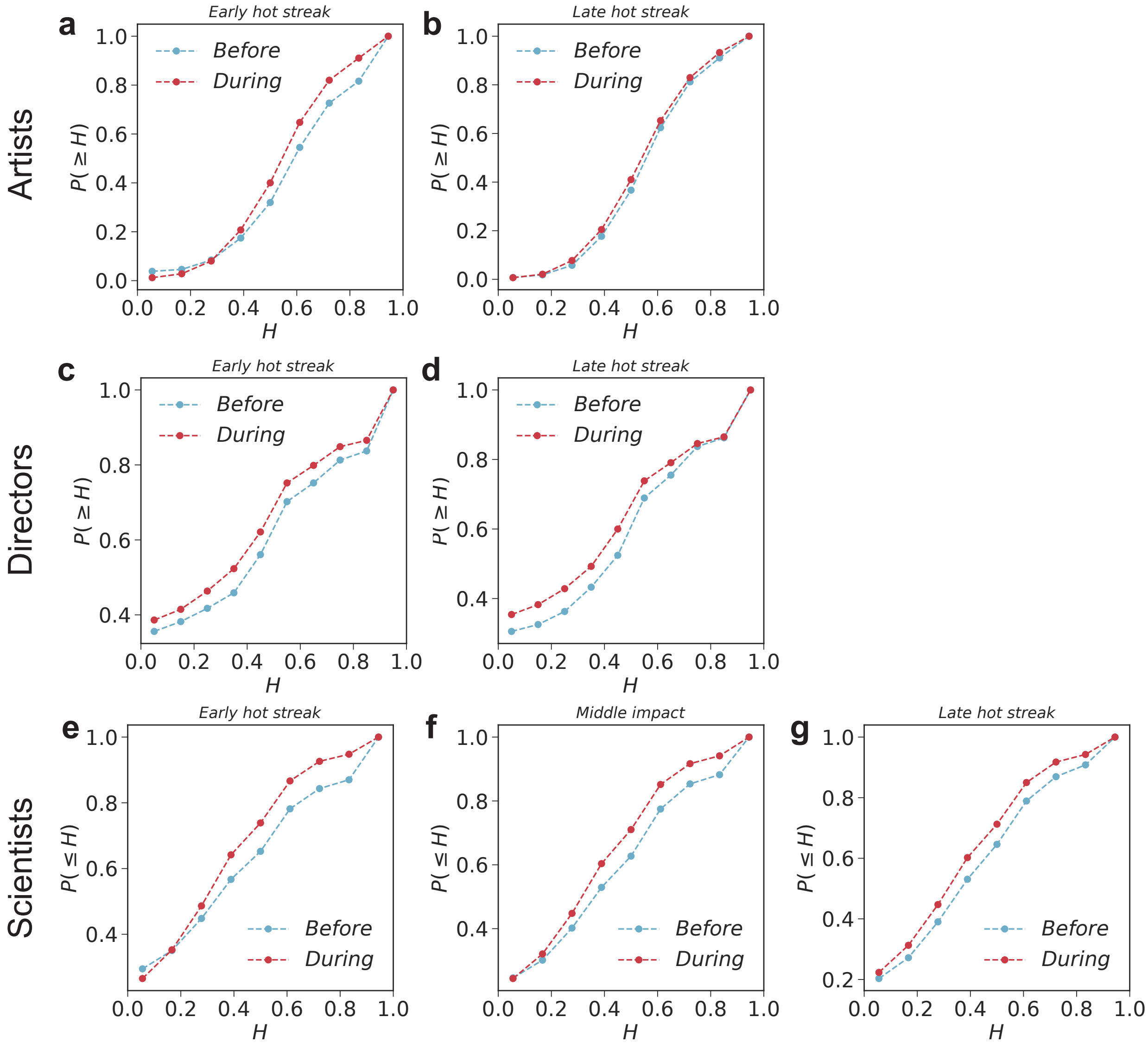

Supplymentary Figure 15: The distribution of topic entropy $P(H)$ for works produced before and during hot streak for individuals with different timing of hot streak. We split artists and directors into early ($N_{\uparrow}/N \leq 1/2$) and late ($N_{\uparrow}/N \leq 1/2$) hot streak. Given that the sample size for scientists is larger than artists and directors, we split scientists into early ($N_{\uparrow}/N \leq 1/3$), middle ($1/3 < N_{\uparrow}/N \leq 2/3$) and late ($N_{\uparrow}/N > 2/3$) hot streak. H before hot streak is consistently larger than H during hot streak.

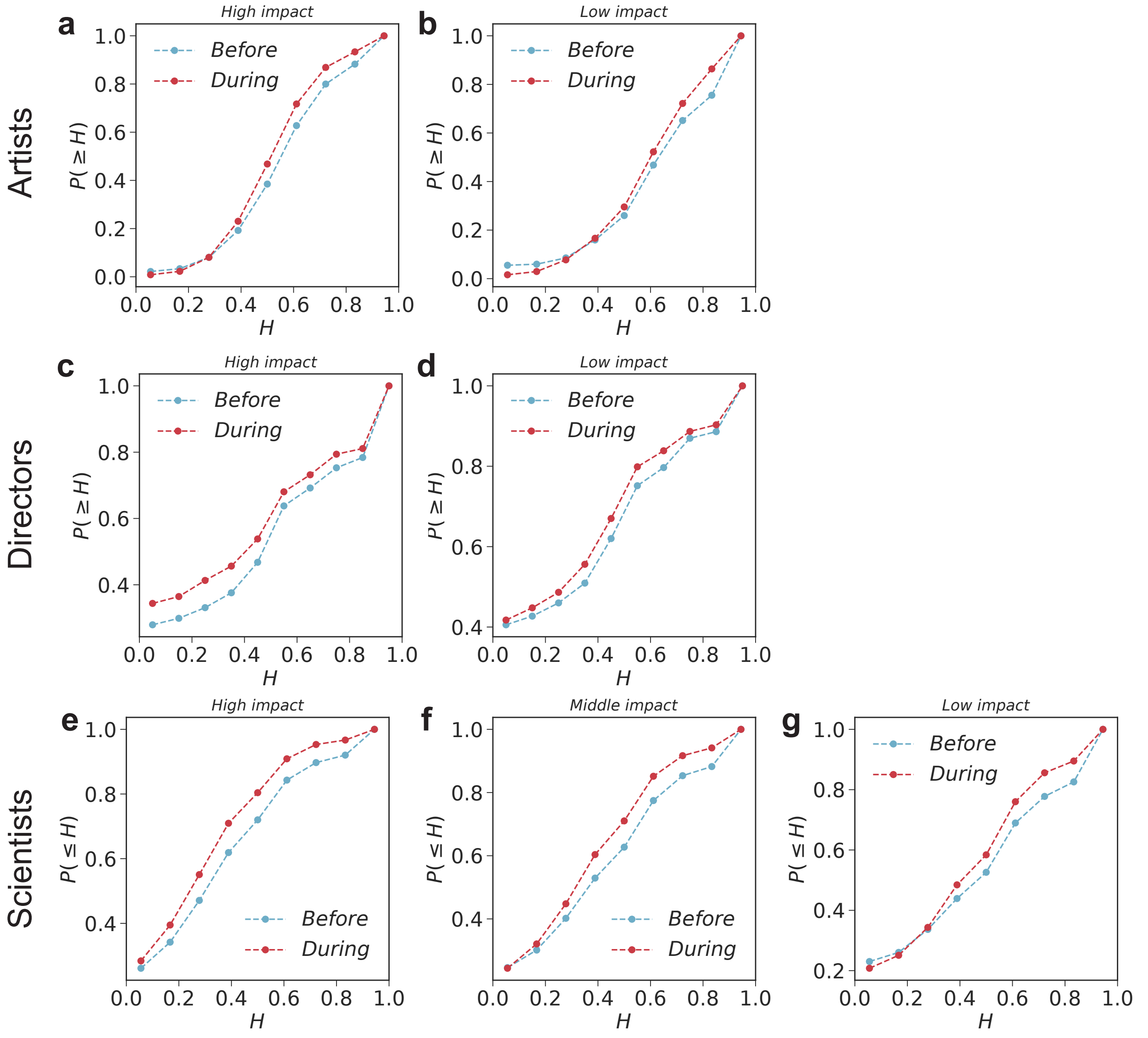

Supplymentary Figure 16: The distribution of topic entropy $P(H)$ for works produced before and during hot streak for individuals with different levels of impact. We split artists and directors into high (top 1/2) and low (bottom 1/2) level of impact. Given that the sample size for scientists is larger than artists and directors, we split scientists into high (top 1/3), low (bottom 1/3), and middle (the rest 1/3) level of impact. H before hot streak is consistently larger than H during hot streak.

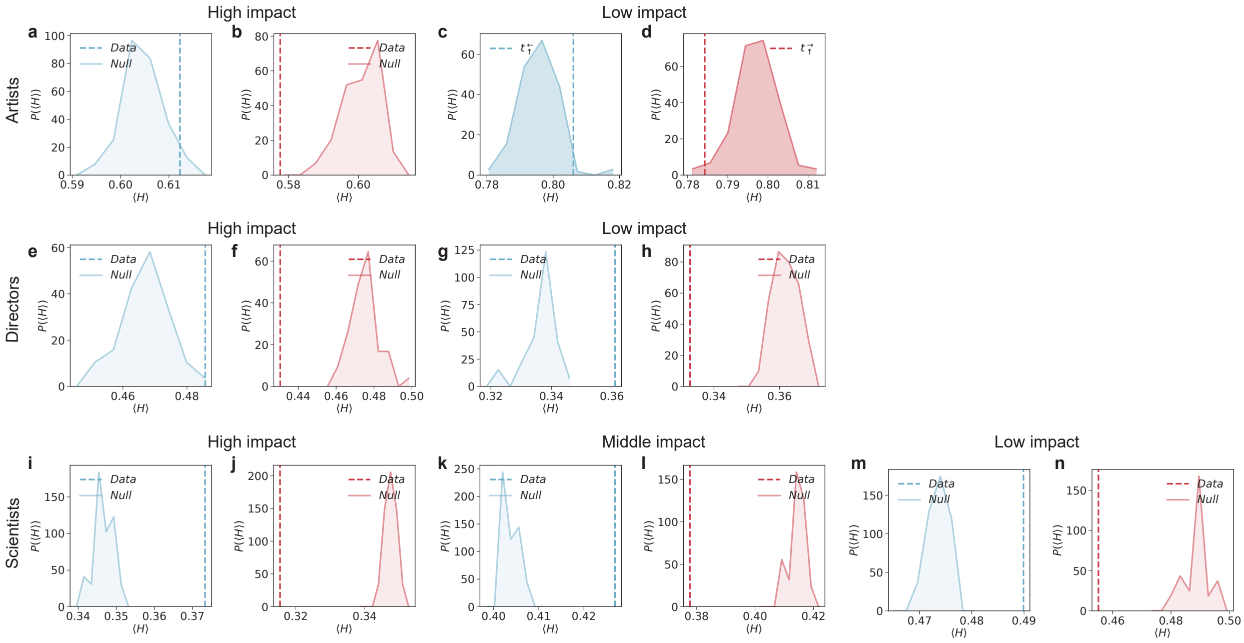

Supplymentary Figure 17: The distribution of rescaled entropy $P(\langle H \rangle)$ before and during hot streak for 1000 realizations of the randomized careers before and during hot streak for individuals with different levels of impact. We split artists and directors into high (top 1/2) and low (bottom 1/2) level of impact. Given that the sample size for scientists is larger than artists and directors, we split scientists into high (top 1/3), low (bottom 1/3), and middle (the rest 1/3) level of impact. $\langle H \rangle$ measured from real careers (vertical line) is larger than expected before hot streak and smaller than expected during hot streak.

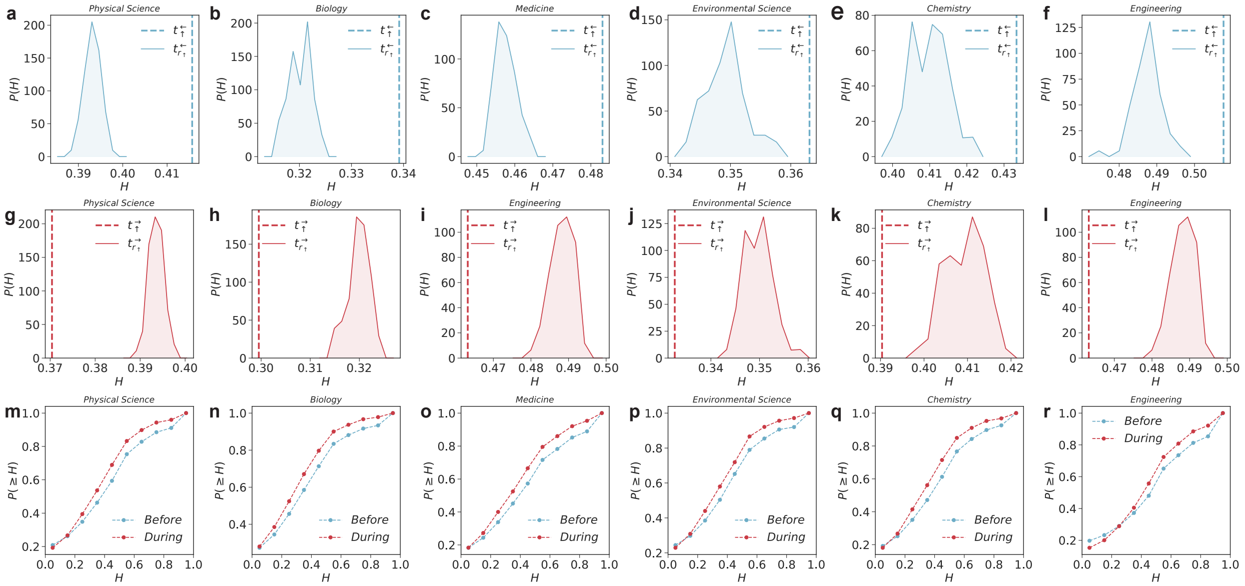

Supplymentary Figure 18: (a-l) The distribution of rescaled entropy $P(\langle H \rangle)$ before and during hot streak for 1000 realizations of the randomized scientific careers before and during hot streak for scientists from six disciplines. $\langle H \rangle$ measured from real careers (vertical line) is larger than expected before hot streak and smaller than expected during hot streak. (m-r) The distribution of topic entropy $P(H)$ for works produced before and during hot streak for scientists from six disciplines. H before hot streak is consistently larger than H during hot streak.

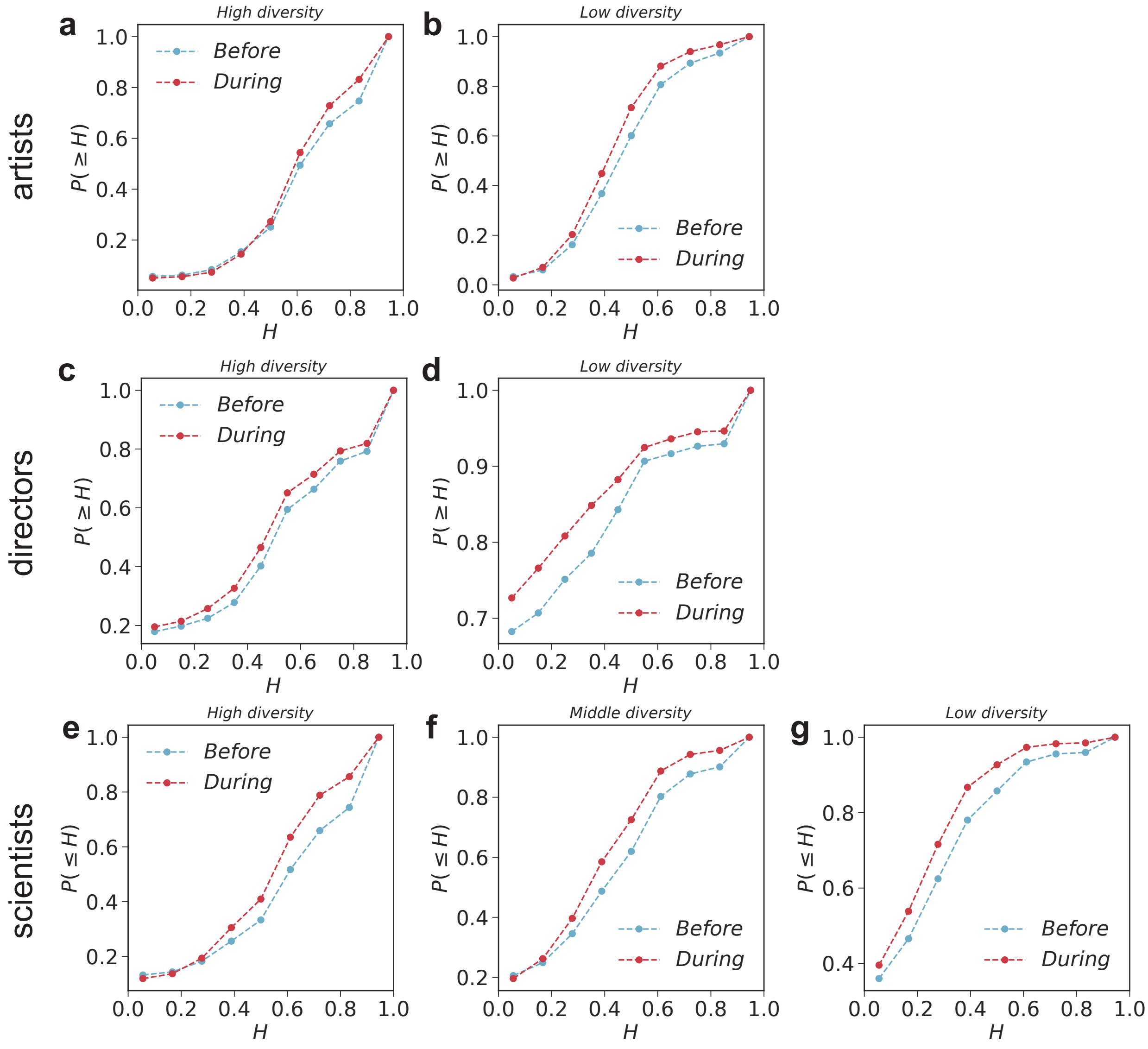

Supplymentary Figure 19: The distribution of topic entropy $P(H)$ for works produced before and during hot streak for individuals with high (top 1/2) and low (bottom 1/2) baseline exploration rates n_i/N. Given that the sample size for scientists is larger than artists and directors, we split scientists into high (top 1/3), low (bottom 1/3), and middle (the rest 1/3) baseline exploration rates. H before hot streak is consistently larger than H during hot streak.

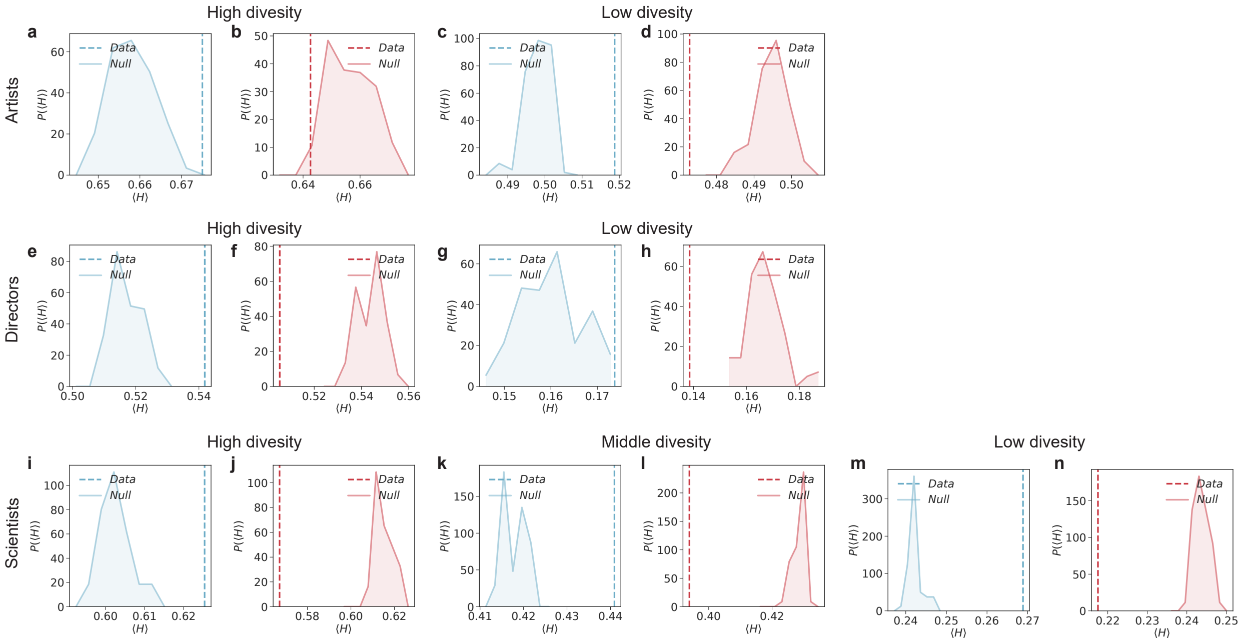

Supplymentary Figure 20: The distribution of rescaled entropy $P(\langle H \rangle)$ before and during hot streak for 1000 realizations of the randomized careers before and during hot streak for individuals with high (top 1/2) and low (bottom 1/2) baseline exploration rates n_i/N. Given that the sample size for scientists is larger than artists and directors, we split scientists into high (top 1/3), low (bottom 1/3), and middle (the rest 1/3) baseline exploration rates. $\langle H \rangle$ measured from real careers (vertical line) is significantly larger than expected before hot streak and smaller than expected during hot streak.

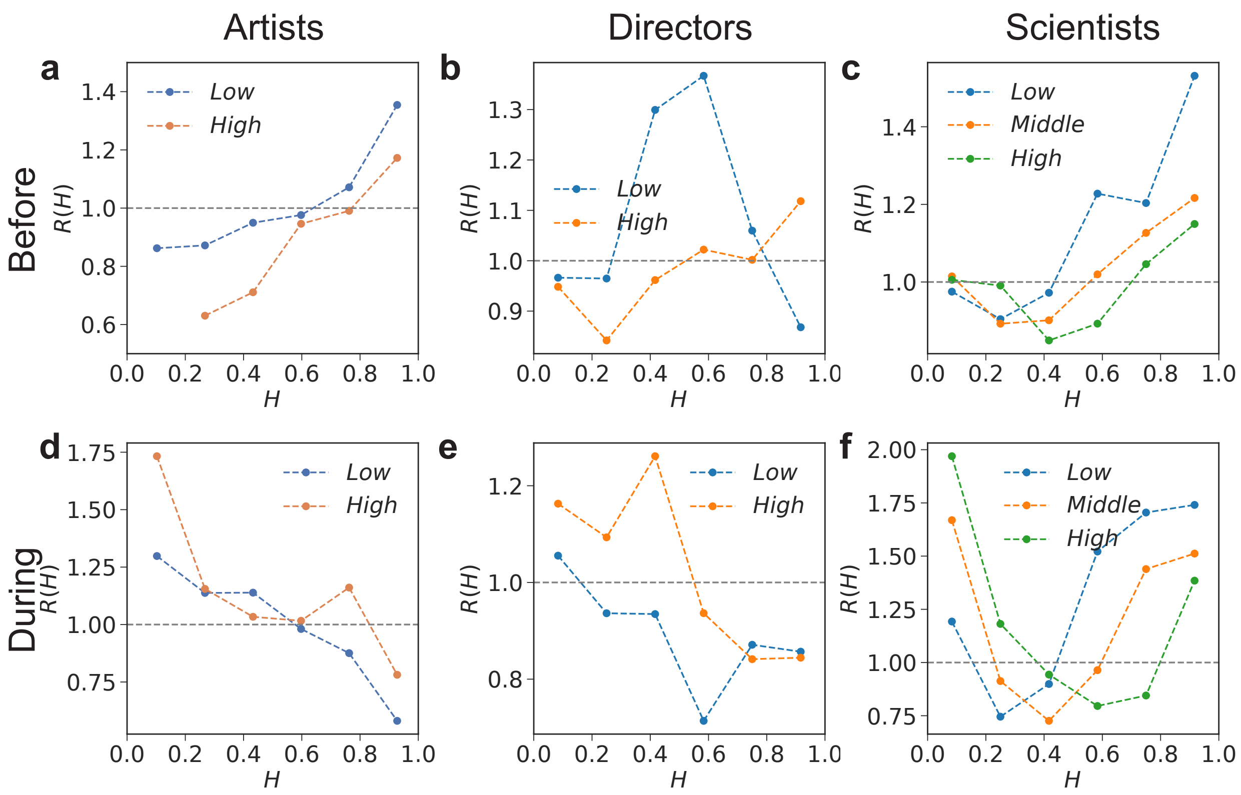

Supplymentary Figure 21: We compare the relative entropy distribution between data $P(H)$ and the null model $P_r(H)$, denoted as $R(H) = P(H)/P_r(H)$ for works produced (a-c) before and (d-f) during hot streak. Individuals with low exploration rates tend to explore more than expected before hot streak, and individuals with high exploration rates tend to exploit more than expected during hot streak.

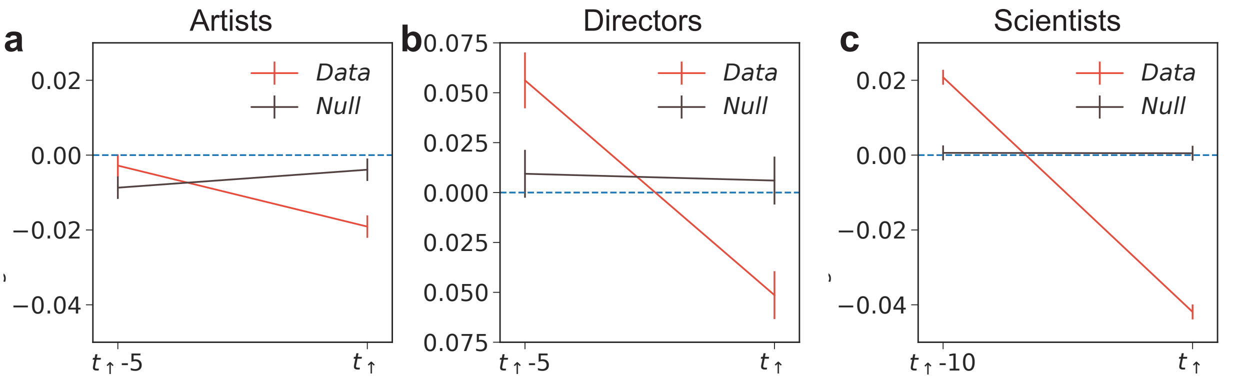

Supplymentary Figure 22: Regression coefficient of entropy change on the timing of hot streak from linear regressions for data and the null model.

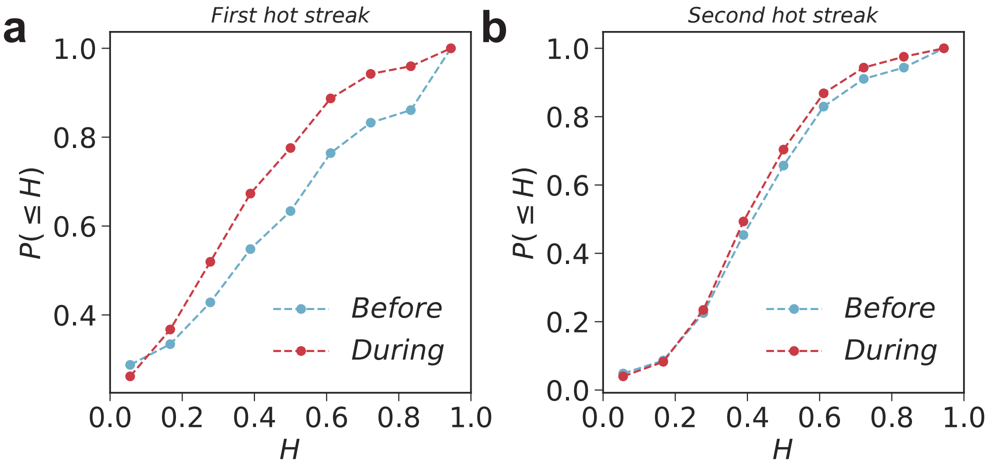

Supplementary Figure 23: The distribution of topic entropy $P(H)$ for works produced before and during the (a) first and (b) second hot streak for scientists.

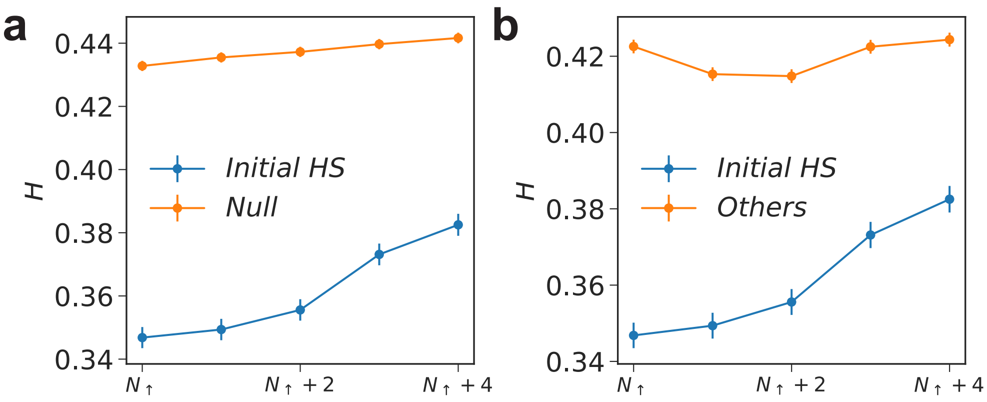

Supplementary Figure 24: The dynamics of average entropy in a sliding window of 5 papers for scientists with hot streak initiating at the beginning of their careers, compared to the entropy at the beginning of a career for (a) the null model, and (b) other scientists whose hot streak come later.

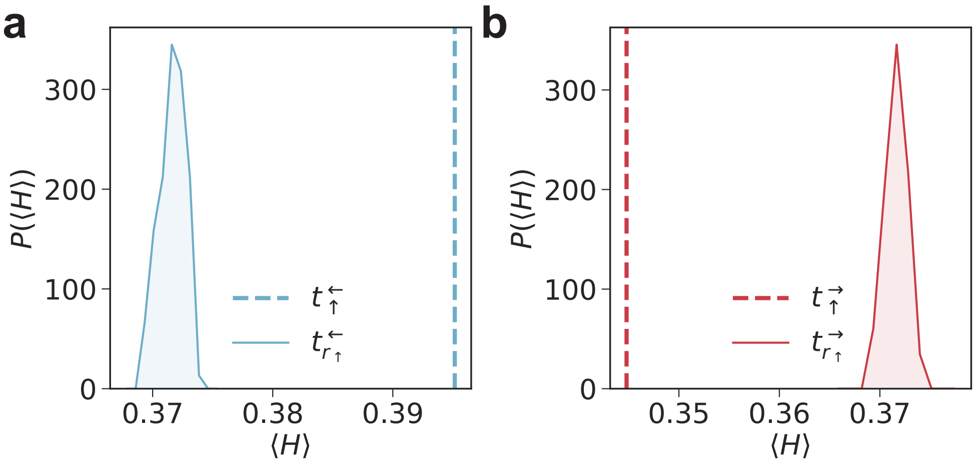

Supplymentary Figure 25: The distribution of rescaled entropy $P(\langle H \rangle)$ before and during hot streak for 1000 realizations of the randomized scientific careers before and during hot streak using topics measured by Infomap. $\langle H \rangle$ measured from real careers (vertical line) is significantly larger than expected before hot streak and smaller than expected during hot streak.

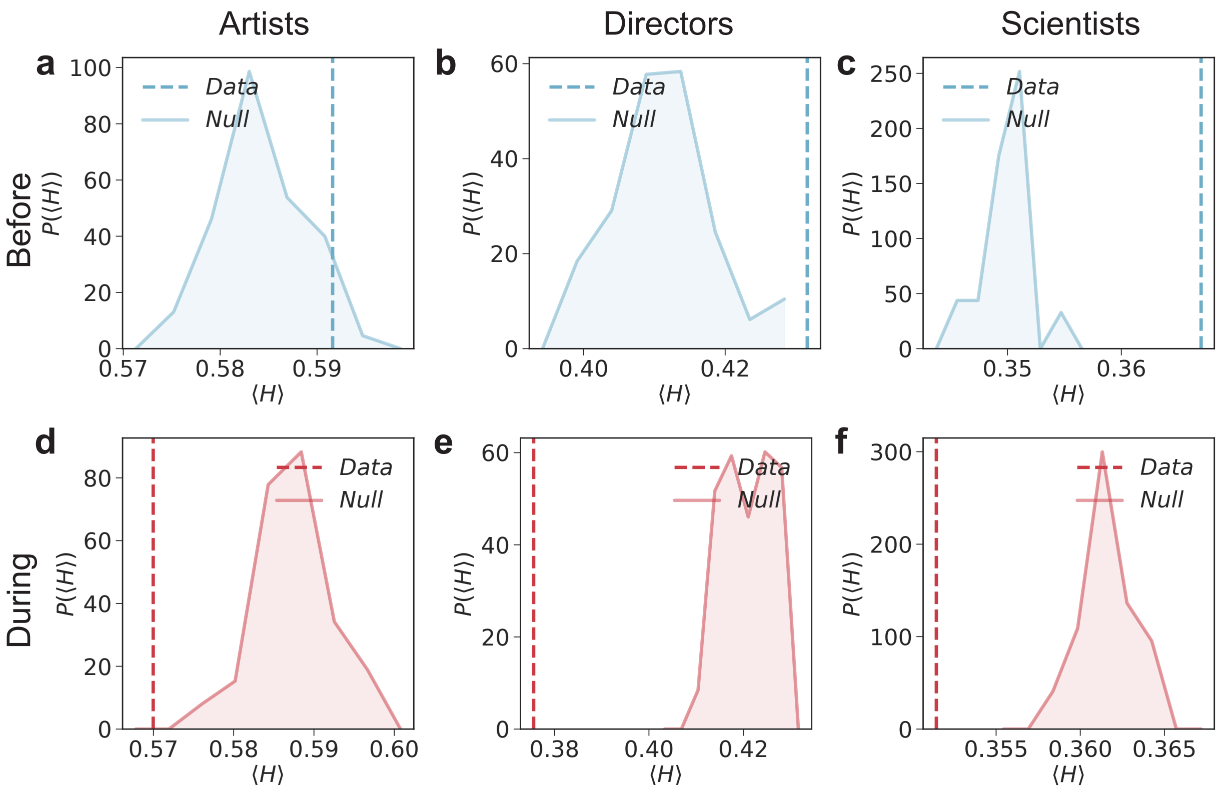

Supplymentary Figure 26: The distribution of rescaled entropy $P(\langle H \rangle)$ before and during hot streak for 1000 realizations of the randomized careers measured by fixed five-year time window before and after the onset of hot streak.

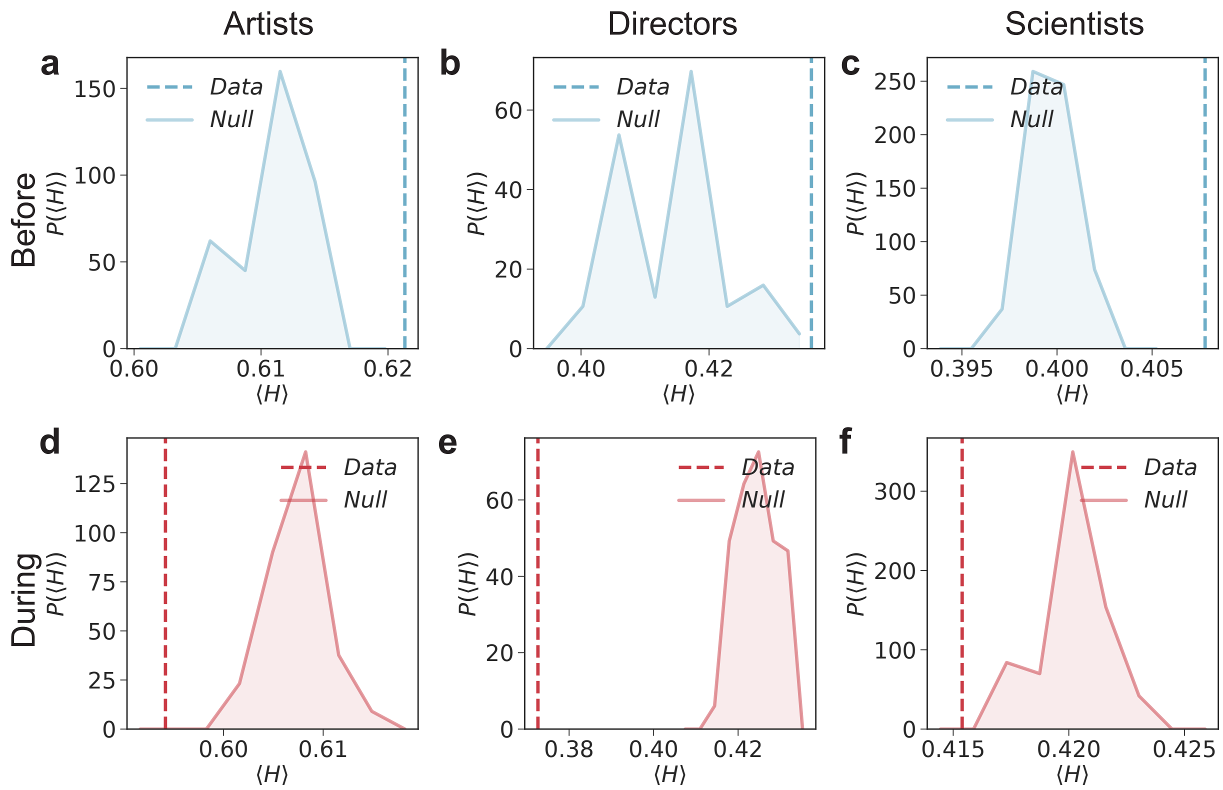

Supplymentary Figure 27: The distribution of rescaled entropy $P(\langle H \rangle)$ before and during hot streak for 1000 realizations of the randomized careers measured by fixed sample size before and after the onset of hot streak (8 for artists, 5 for directors and scientists).

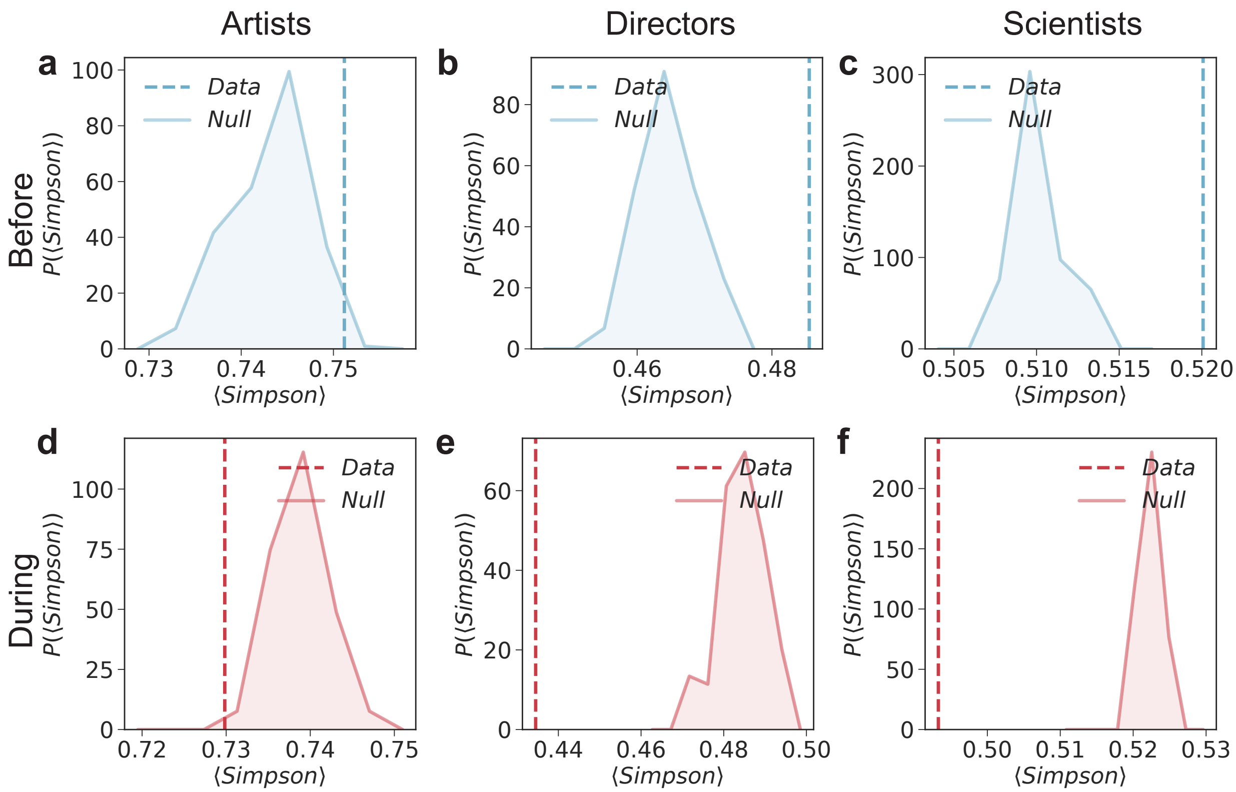

Supplymentary Figure 28: The distribution of Simpsons diversity $P(\langle Simpsons \rangle)$ before and during hot streak for 1000 realizations of the randomized careers for the three domains. $\langle Simpsons \rangle$ measured from real careers (vertical line) is significantly larger than expected before hot streak (a-c) and smaller than expected during hot streak (d-f).

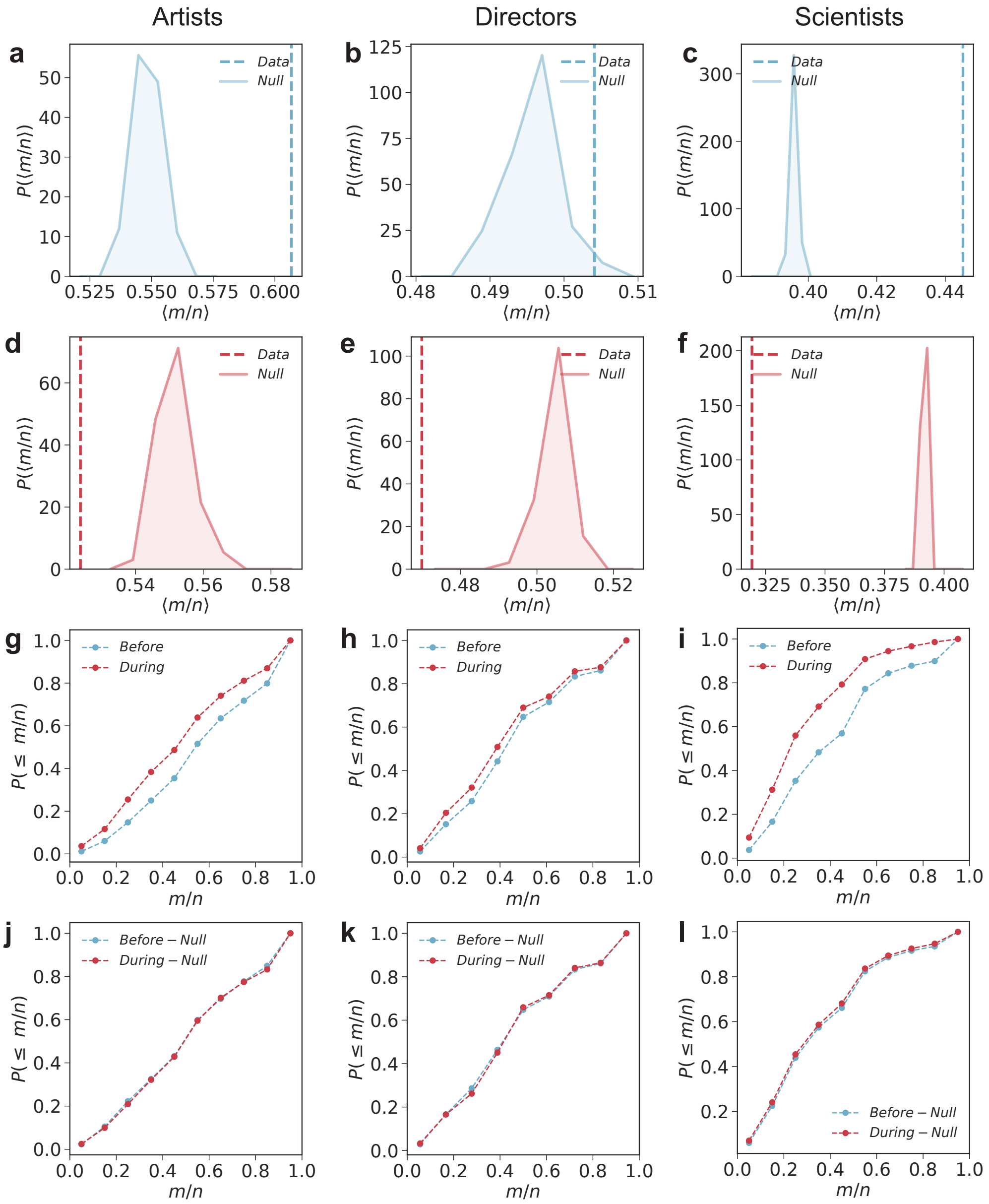

Supplymentary Figure 29: (a-f) The distribution of number of topics normalized by productivity $P(\langle m/n \rangle)$ before and during hot streak for 1000 realizations of the randomized careers for the three domains. $\langle m/n \rangle$ measured from real careers (vertical line) is significantly larger than expected before hot streak (a-c) and smaller than expected during hot streak (d-f). (g-l) Cumulative distribution $P(\leq m/n)$ for (g-i) data and (j-l) the randomized careers.

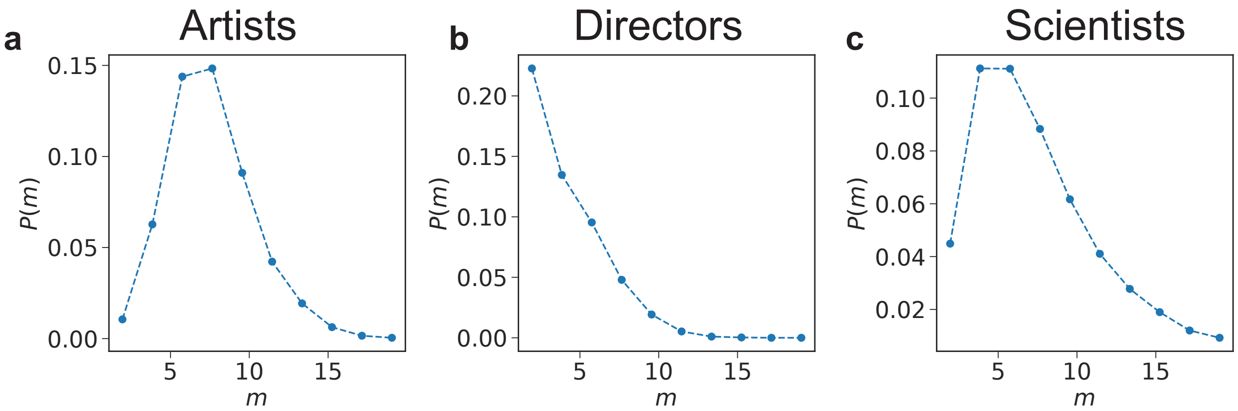

Supplymentary Figure 30: The distribution for the number of styles/topics in a career.

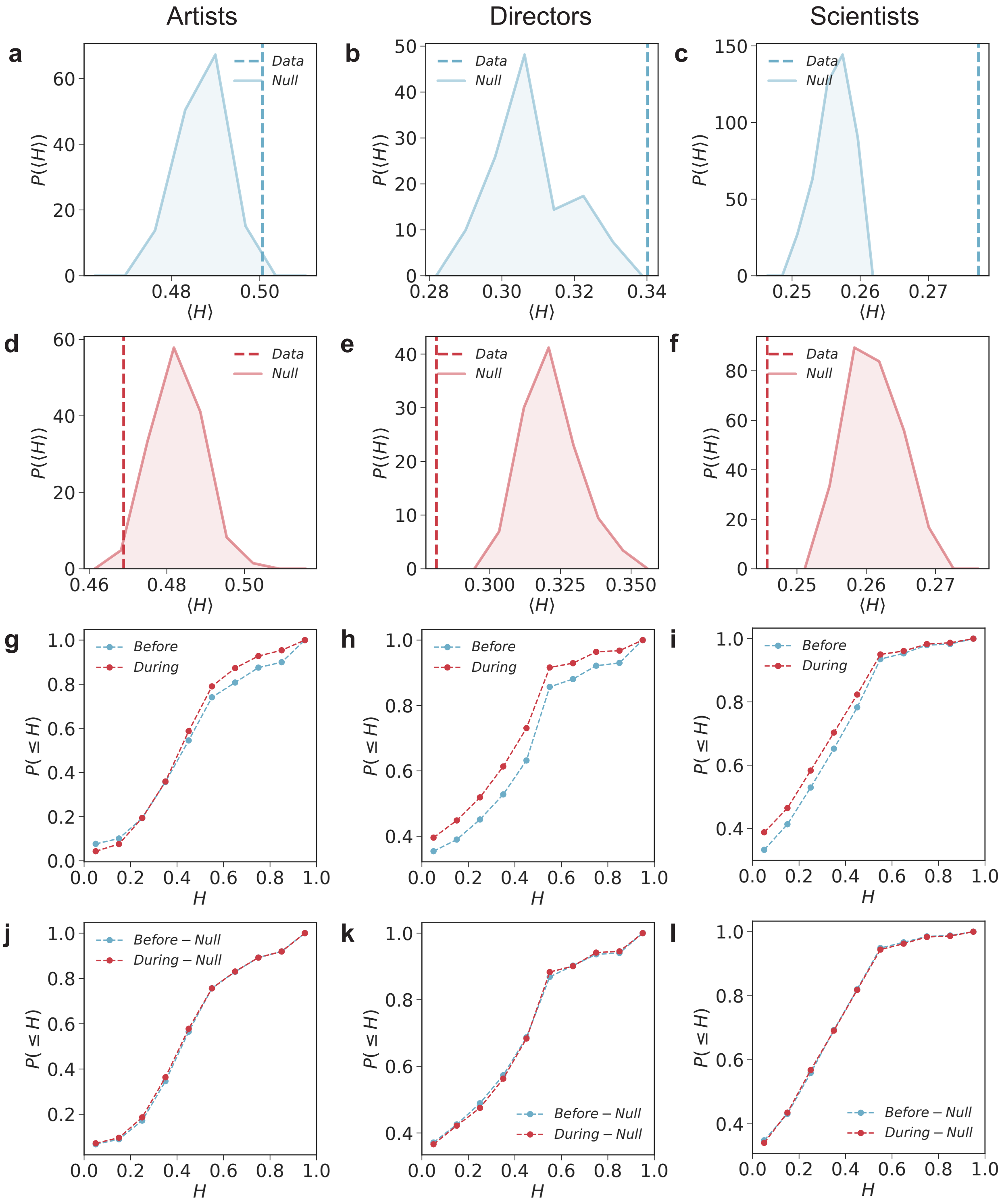

Supplymentary Figure 31: The distribution for the number of styles/topics in a career.

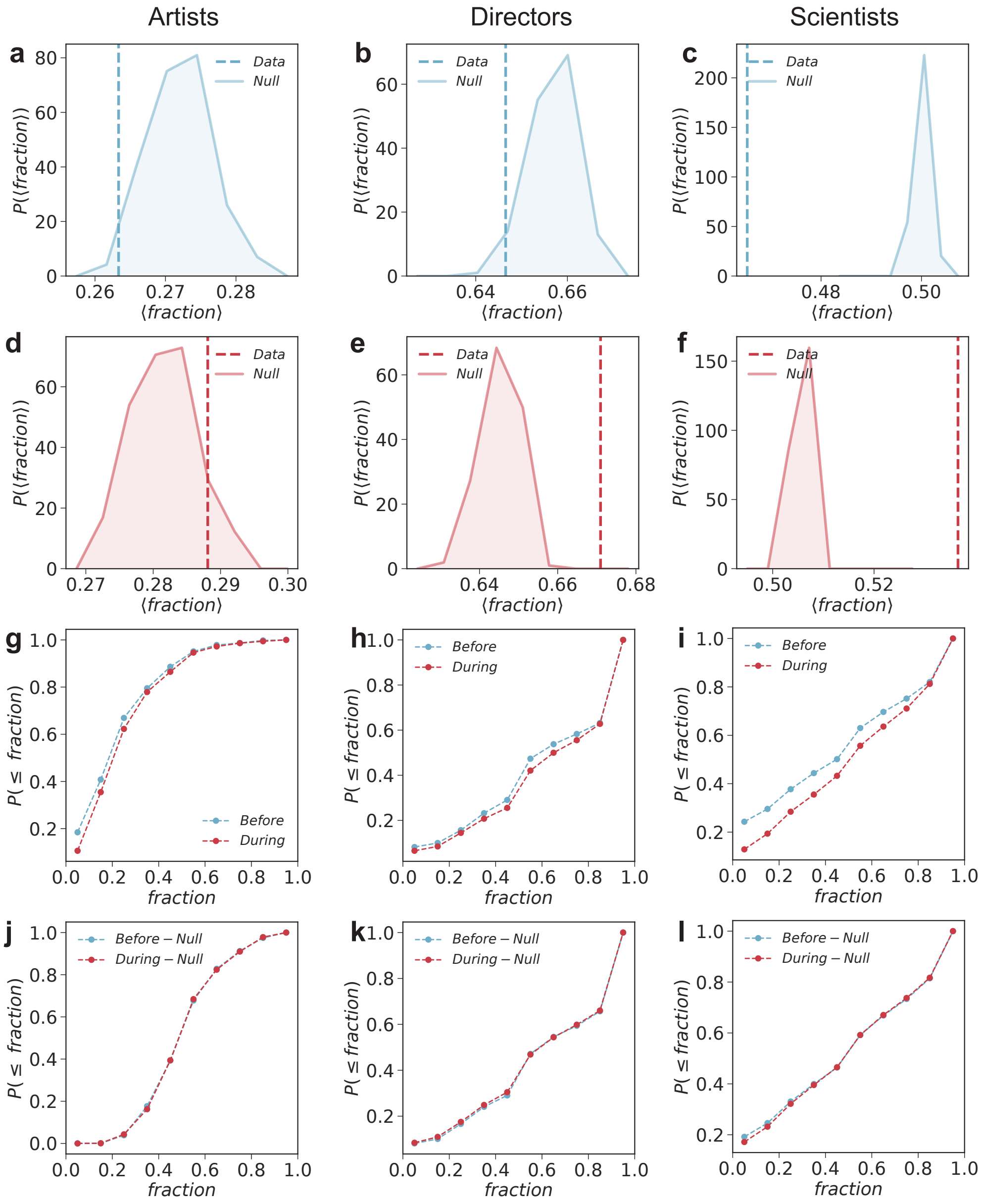

Supplymentary Figure 32: (a-f) The distribution of average fraction in the most popular topic $P(\langle fraction \rangle)$ before and during hot streak for 1000 realizations of the randomized careers for the three domains. $\langle fraction \rangle$ measured from real careers (vertical line) is significantly larger than expected before hot streak (a-c) and smaller than expected during hot streak (d-f). (g-l) Cumulative distribution $P(\leq fraction)$ for (g-i) data and (j-l) the randomized careers.

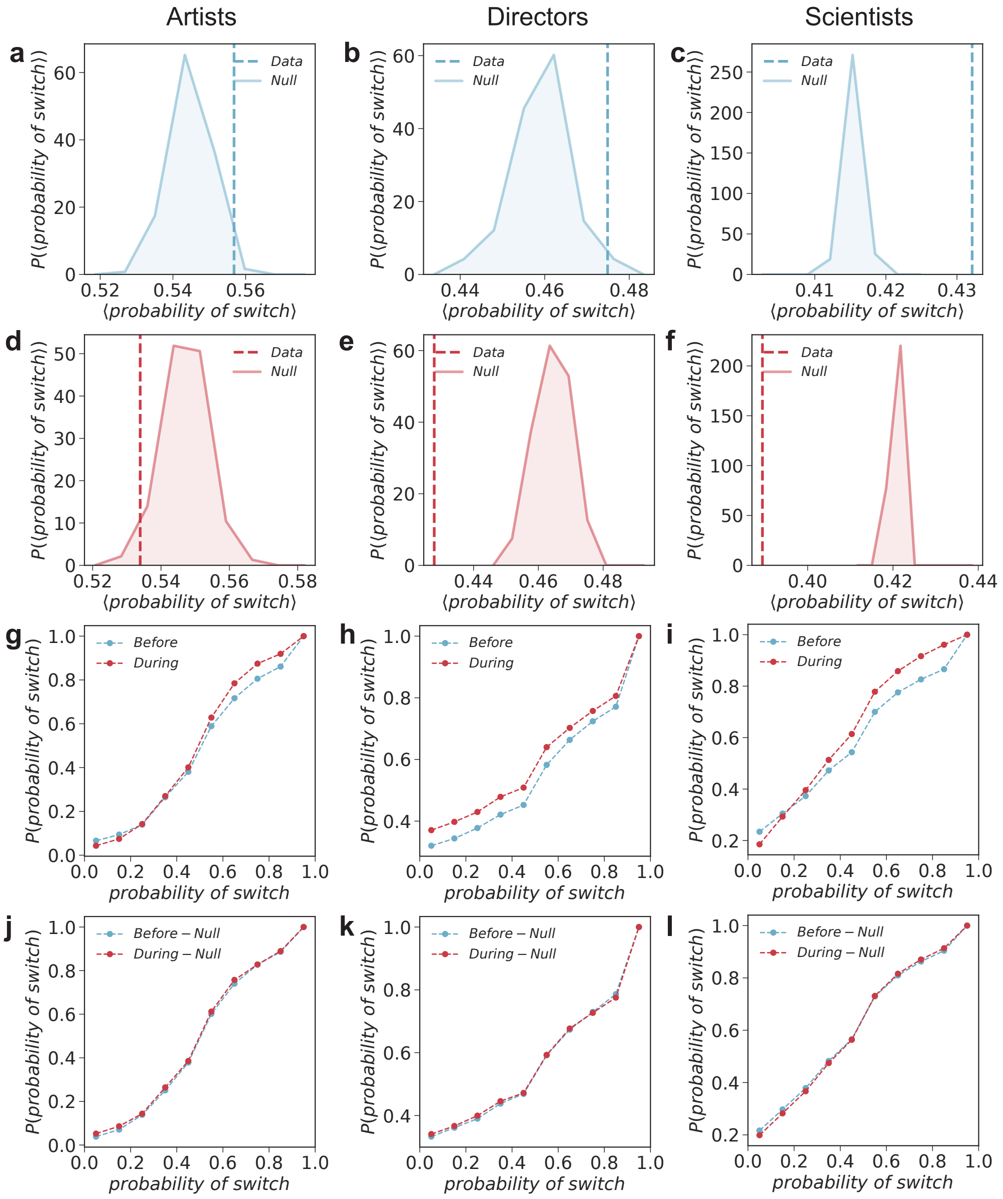

Supplymentary Figure 33: (a-f) The distribution of switching probability before and during hot streak for 1000 realizations of the randomized careers for the three domains. $\langle Probabilityofswitch \rangle$ measured from real careers (vertical line) is significantly larger than expected before hot streak (a-c) and smaller than expected during hot streak (d-f). (g-l) Cumulative distribution $P(\leq probabilityofswitch)$ for (g-i) data and (j-l) the randomized careers.

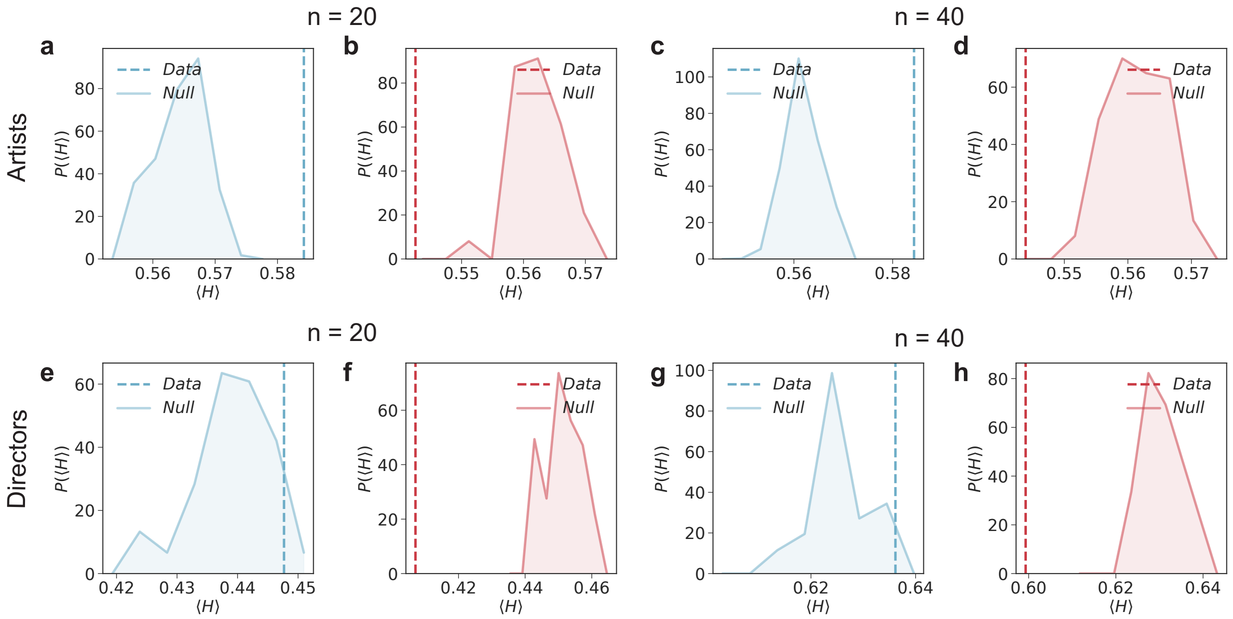

Supplymentary Figure 34: The distribution of rescaled entropy $P(\langle H \rangle)$ before and during hot streak for 1000 realizations of the randomized careers using different numbers of centroids n for (a-d) artists and (e-h) directors. $\langle H \rangle$ measured from real careers (vertical line) is significantly larger than expected before hot streak and smaller than expected during hot streak.

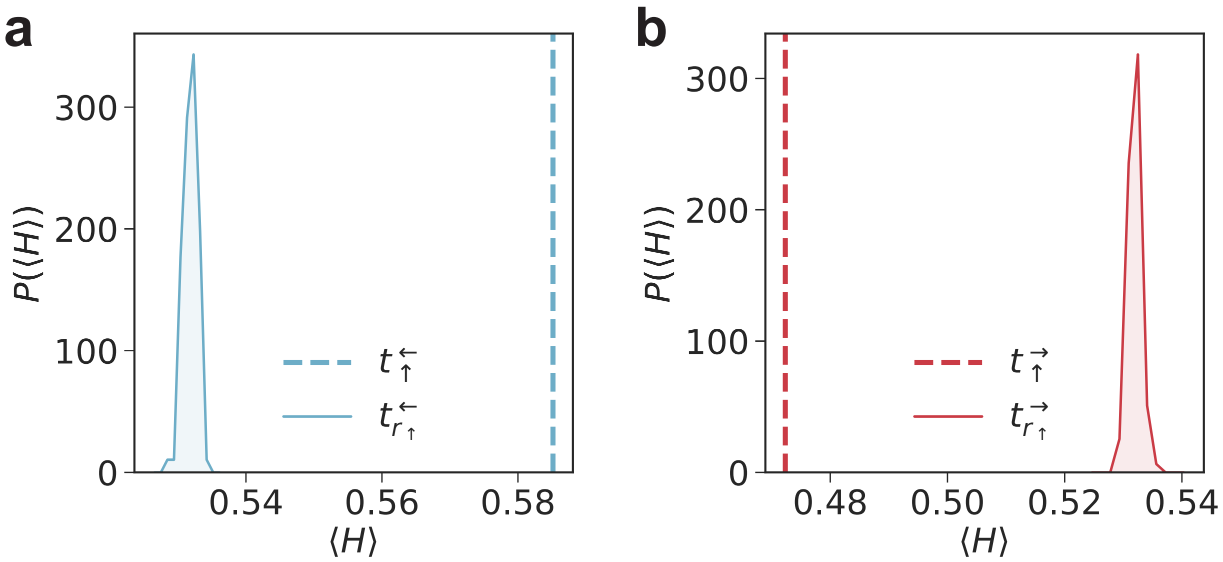

Supplymentary Figure 35: The distribution of rescaled entropy $P(\langle H\rangle)$ (a) before and (b) during hot streak for 1000 realizations of the randomized scientific careers including papers without references. $\langle H\rangle$ measured from real careers (vertical line) is significantly larger than expected before hot streak and smaller than expected during hot streak.

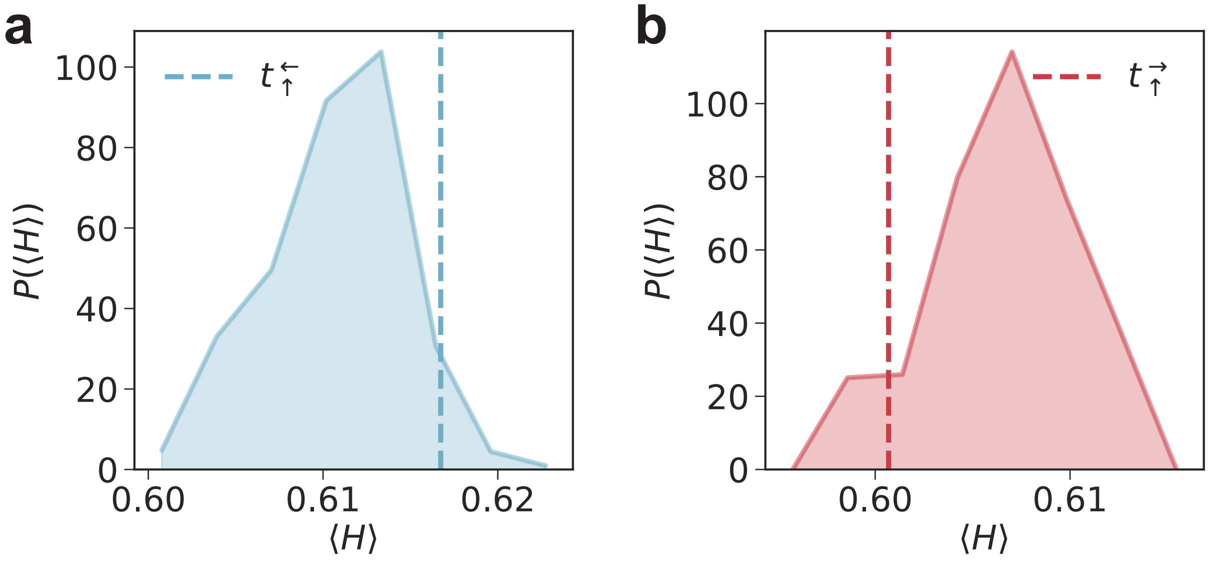

Supplymentary Figure 36: The distribution of rescaled entropy $P(\langle H\rangle)$ (a) before and (b) during hot streak for 1000 realizations of the randomized artistic careers using art style labels predicted by the VGG16. $\langle H\rangle$ measured from real careers (vertical line) is significantly larger than expected before hot streak and smaller than expected during hot streak.

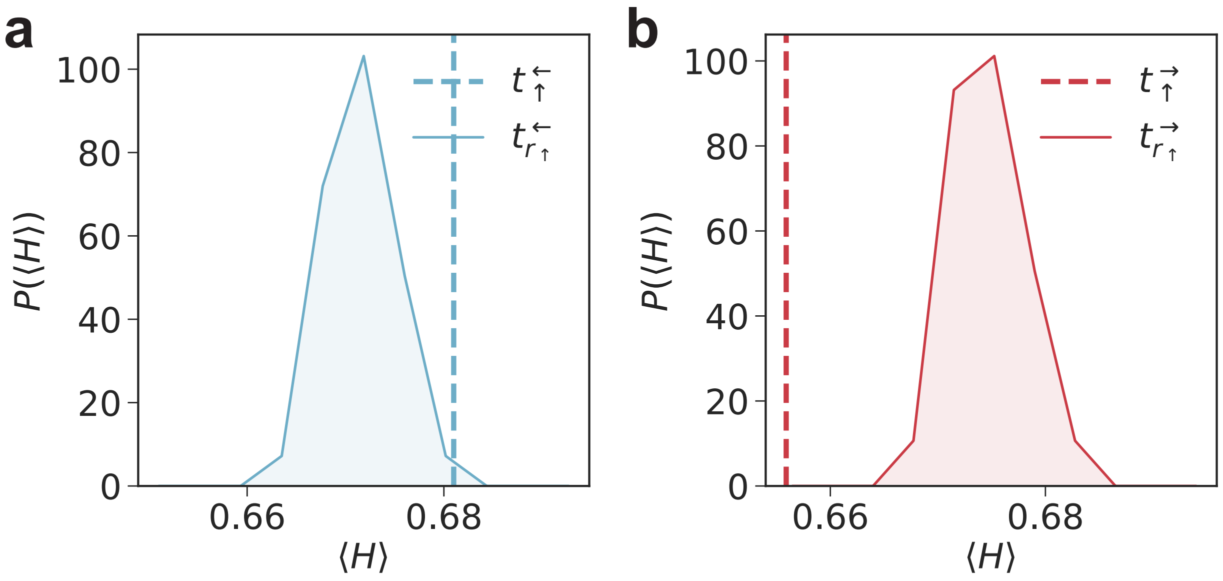

Supplymentary Figure 37: The distribution of rescaled entropy $P(\langle H \rangle)$ (a) before and (b) during hot streak for 1000 realizations of the randomized directors' careers using film genre labels. $\langle H \rangle$ measured from real careers (vertical line) is significantly larger than expected before hot streak and smaller than expected during hot streak.

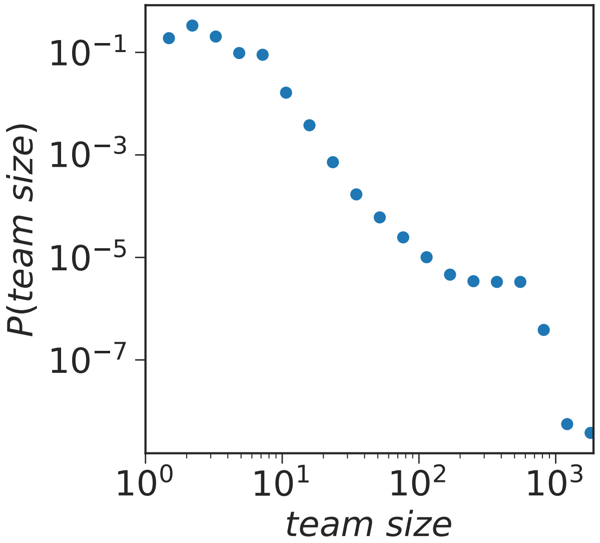

Supplymentary Figure 38: Log-log plot of the team size distribution

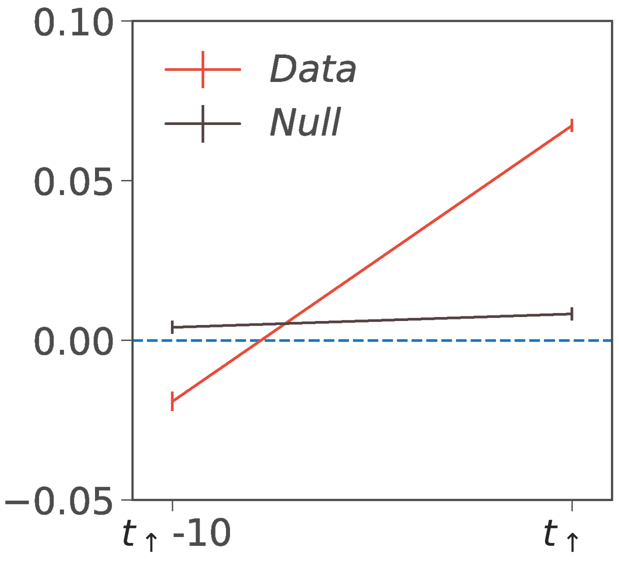

Supplymentary Figure 39: Regression coefficient of team size change on the timing of hot streak from linear regressions for data and the null model.

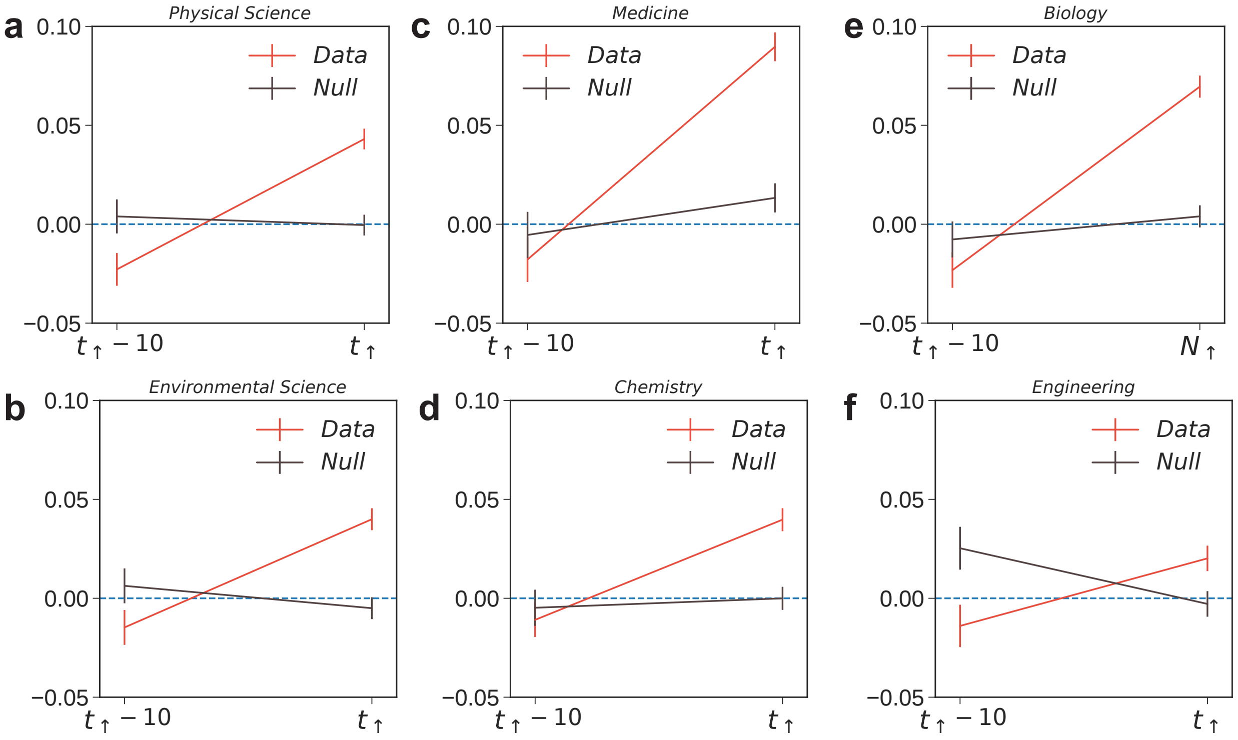

Supplymentary Figure 40: Regression coefficient of team size change on the timing of hot streak from linear regressions for scientists from six different disciplines.

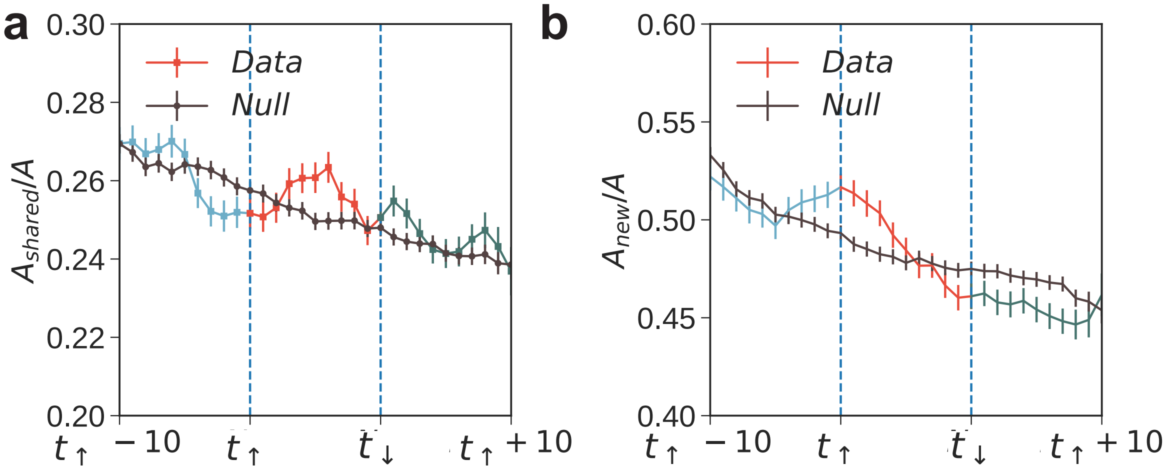

Supplymentary Figure 41: (a) The turnover rate of coauthors and (b) the rate of new collaborators around hot streak.

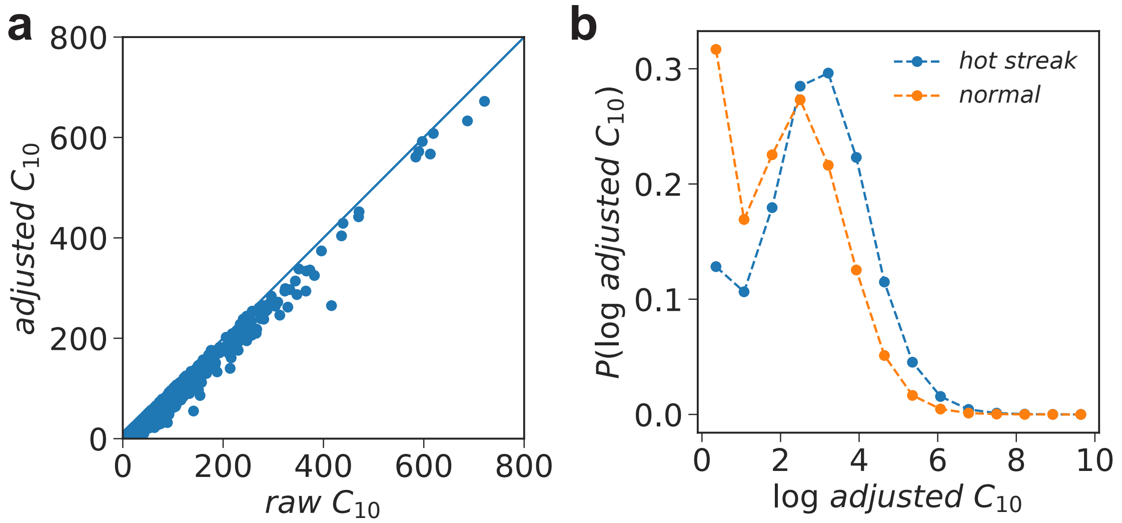

Supplymentary Figure 42: (a) Scatter plot between raw and self-citation adjusted C_{10} for 10000 random papers in the dataset. (b) The distribution of logarithmic self-citation adjusted C_{10} for papers published during hot streaks and normal phases.

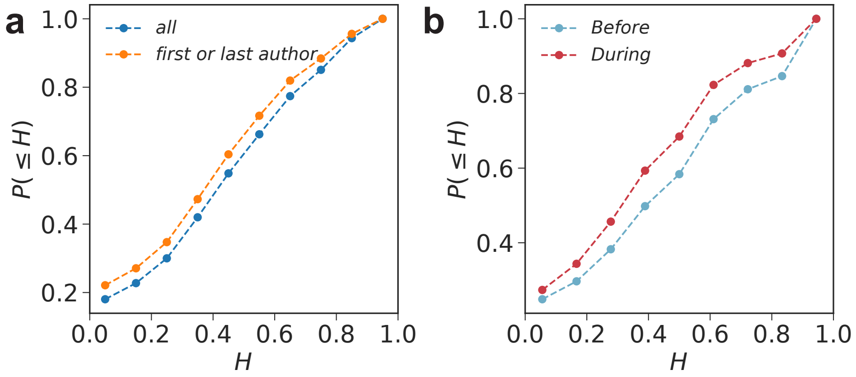

Supplymentary Figure 43: (a) Cumulative distribution of entropy H for all papers during hot streaks and the first and last-authorship papers. (b) Cumulative entropy distribution before and during a hot streak for lead-author papers.

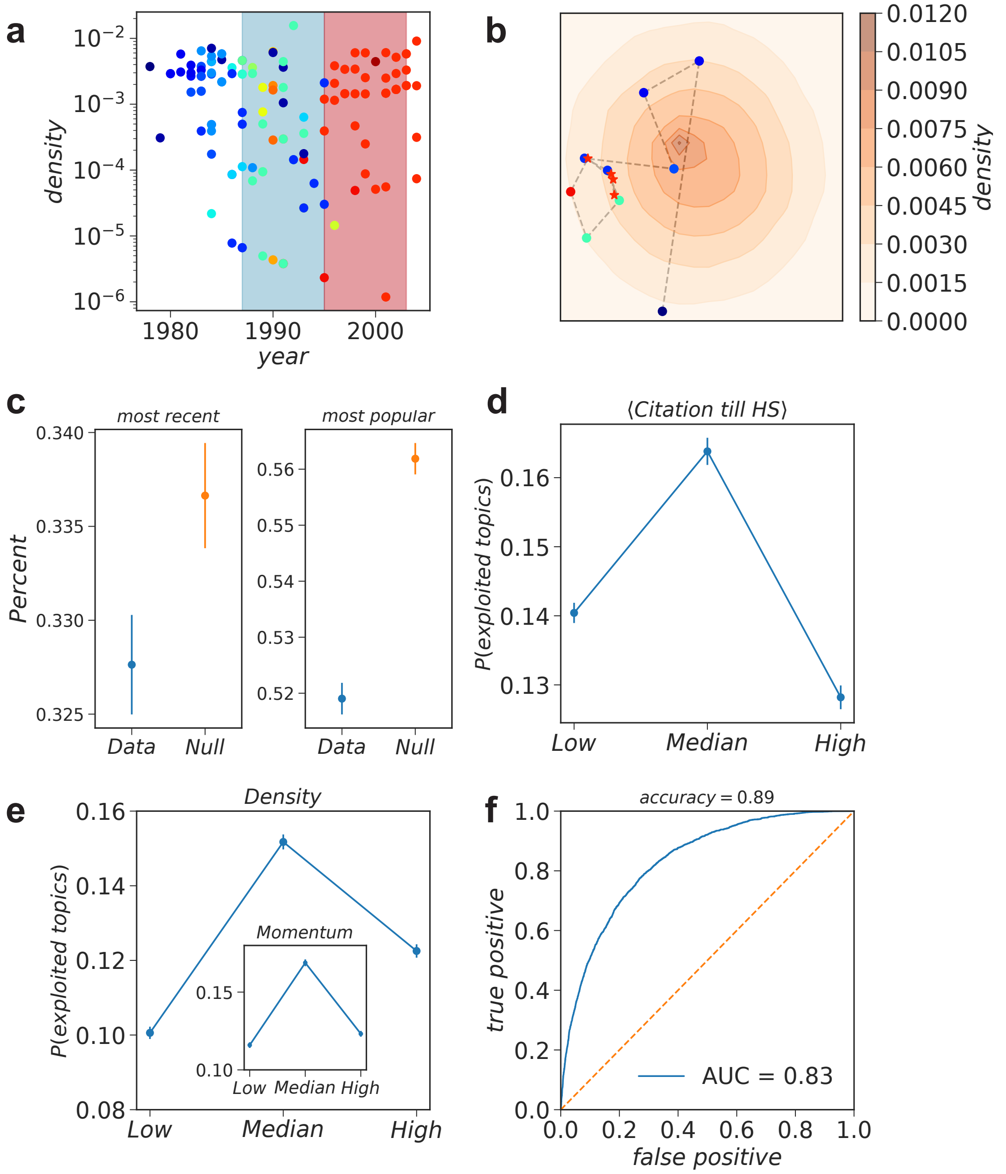

Supplymentary Figure 44: (a) The dynamics of paper density within a career for a scientist in our dataset. (b) The topic trajectory in the embedding space by the same scientist in (a). Each data point denotes the average location for papers published in a two-year window. Stars denote her hot streak and dots denote the normal period. Color captures the most frequent topic in each window. (c) The probability for the topic exploited to be the (left) most recently initiated and (right) most frequently studied before a hot streak begins for real and randomized careers. (d) The probability for the topic exploited to be low-, median- and high-impact among prior topics. (e) The probability for the topic exploited to fall into low-, median- and high-density region or (Inset) momentum among prior explored topics. (f) The ROC curve for the prediction task.

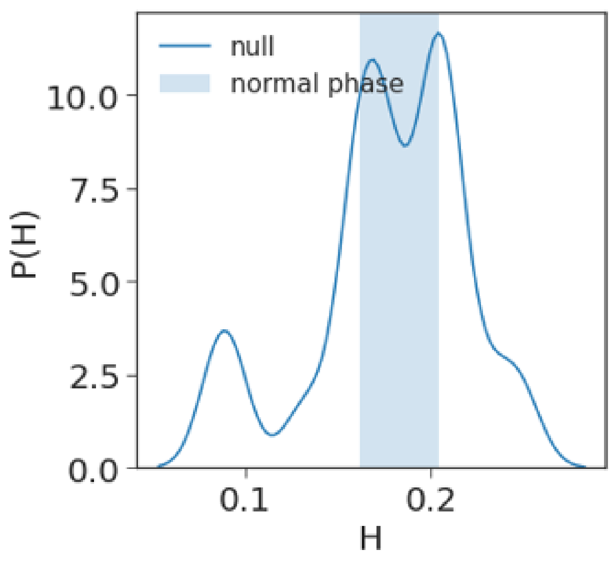

Supplymentary Figure 45: Illustration on how to define normal, exploration and exploitation phase. For any period of the same length in real careers, we compare its entropy to the null model distribution, and assign it as exploitation if the entropy is below the 25th quantile of the null model, normal phase if the entropy is between the 25th and 75th quantile, and exploration if the entropy is above the 75th quantile.

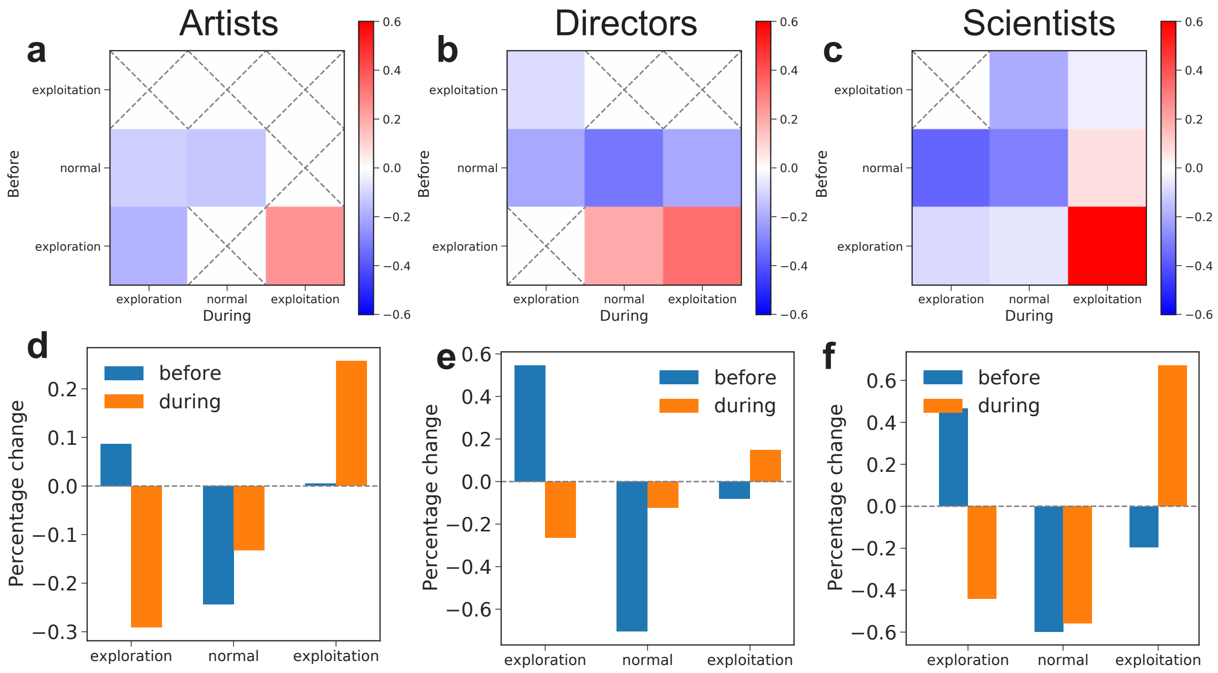

Supplymentary Figure 46: (a-c) Comparing the probability to observe the onset of hot streak for different combinations of exploration, normal and exploitation phases to the baseline. Color denotes the percentage change, where blue is below the baseline and red is above the baseline. Dashed box indicates the percentage change is not significantly different zero (chi-square test, $p-value \geq 0.05$) (d-f) The total percentage change of exploration, normal and exploitation phases before and during the hot streaks. The value of the bar plot is the sum of the matrix in each row (before) and each column (during).

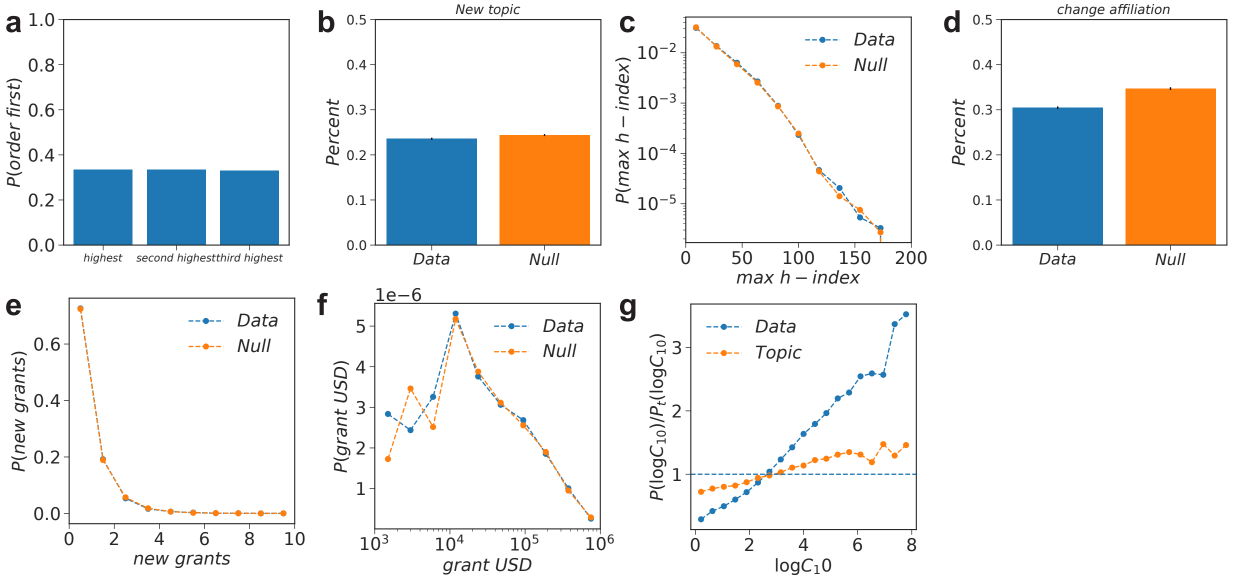

Supplymentary Figure 47: (a) We measure the relative position for the top three highest impact papers during hot streak, and calculate their probability to order first among the three. We find equal probability for the three highest impact papers to appear first. (b) The probability for the hot streak to begin with a new topic for data and the null model. (c) The distribution of the highest h-index among collaborators for publications during hot streak in real and randomized careers is virtually indistinguishable. (d) The probability for an individual to work in a new institution during hot streak for data and the null model. (e) The distribution for the number of new grants at the year when a hot streak begins for data and the null model is virtually indistinguishable. (f) The distribution for new funding amount at the year when a hot streak begins for data and the null model is virtually indistinguishable. (g) The ratio between the distribution of topic impact during hot streak and that of the topics before. Compared to the ratio between the distribution of paper impact during hot streak and that of papers before, the improvement of topic impact appears negligible.

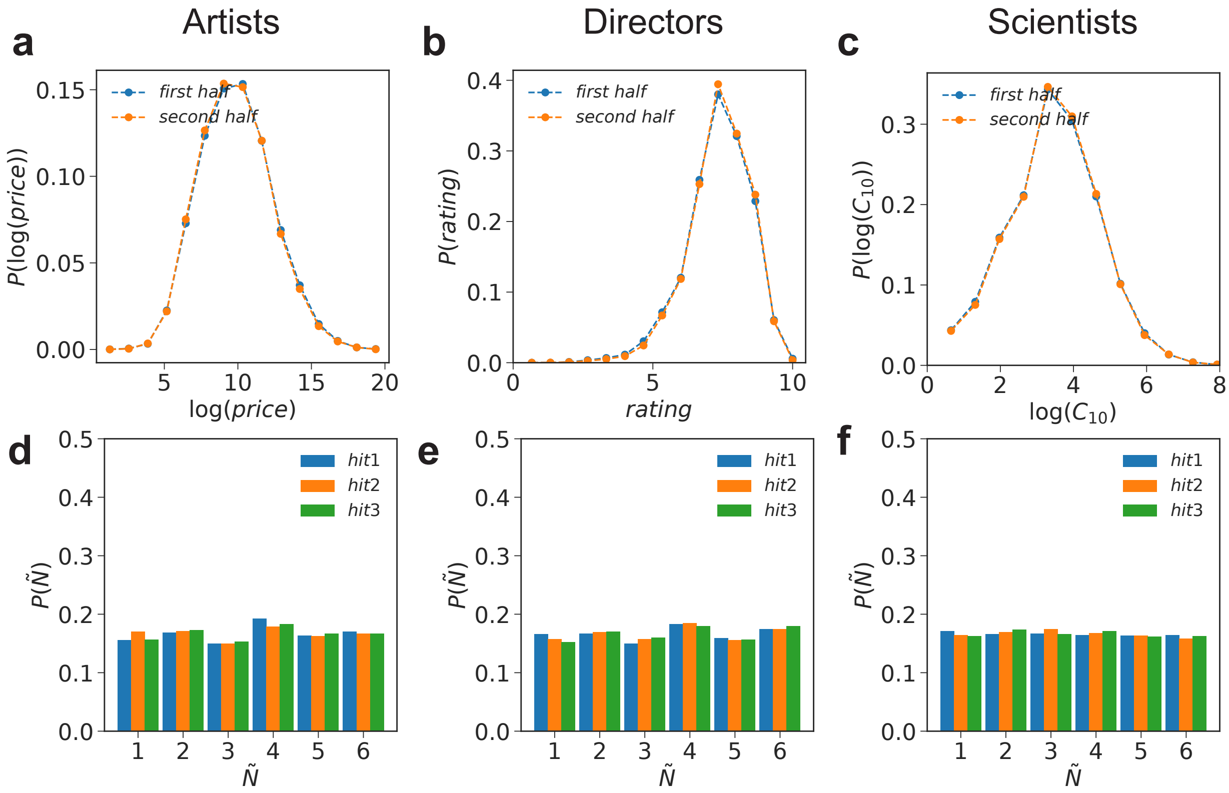

Supplymentary Figure 48: (a-c) The impact distribution for the first and the second half of a hot streak across three domains. (d-f) The distribution of the relative position $P(\tilde{N})$ of the three highest-impact works among the six highest-impact works within a career for artists, where $\tilde{N}$ denotes the relative order among the top six hits.

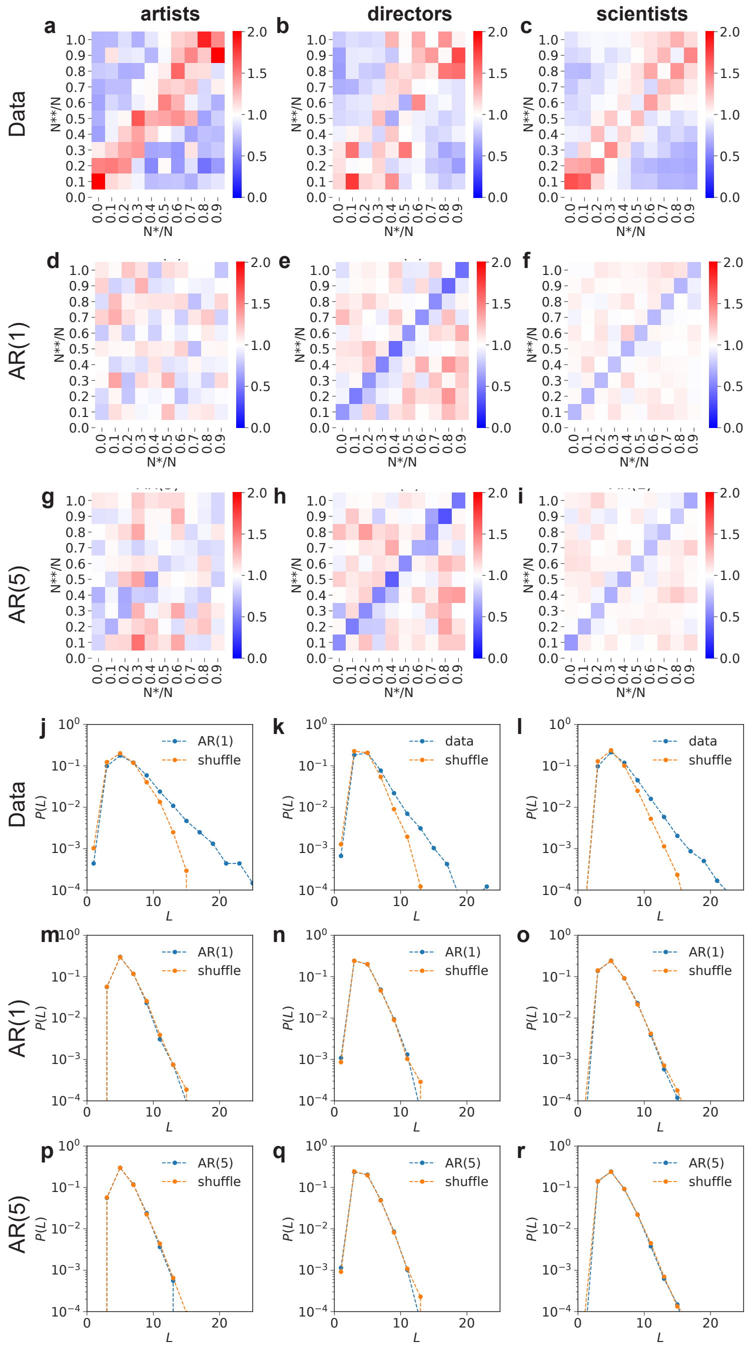

Supplymentary Figure 49: Comparison between autoregressive model and real careers. (a-i) The relative timing between the two biggest hits N^* and N^{**} for (a-c) data, (d-f) $AR(1)$, and (h-i) $AR(5)$. We measured the correlation between the N^* and N^{**}, and compared it with a null hypothesis in which N^* and $N^(**)$ each occurred at random. The matrix denotes the value for the normalized joint probability, $\Phi(N^*, N^{**}) = P(N^*, N^{**})/(P(N^*)P(N^{**}))$. (j-r) The distribution of the length of streaks $P(L)$ of real and shuffled impact sequences for (j-l) data, (m-o) $AR(1)$, and (p-r) $AR(5)$.
