# Supplementary material for: Understanding the onset of hot streaks across artistic, cultural, and scientific careers
Source: Nat Commun. 2021 Sep 13;12:5392. doi: 10.1038/s41467-021-25477-8 (PMC8438033; doi:10.1038/s41467-021-25477-8)
Supplement: Supplementary file 2 — Reporting Summary [file 41467_2021_25477_MOESM2_ESM.pdf]

nature portfolio

Corresponding author(s): Dashun Wang

Last updated by author(s): 2021/07/26

Reporting Summary

Nature Portfolio wishes to improve the reproducibility of the work that we publish. This form provides structure for consistency and transparency in reporting. For further information on Nature Portfolio policies, see our Editorial Policies and the Editorial Policy Checklist.

Statistics

For all statistical analyses, confirm that the following items are present in the figure legend, table legend, main text, or Methods section.

n/a	Confirmed	
☐	☒	The exact sample size (*n*) for each experimental group/condition, given as a discrete number and unit of measurement
☐	☒	A statement on whether measurements were taken from distinct samples or whether the same sample was measured repeatedly
☐	☒	The statistical test(s) used AND whether they are one- or two-sided<br>*Only common tests should be described solely by name; describe more complex techniques in the Methods section.*
☒	☐	A description of all covariates tested
☒	☐	A description of any assumptions or corrections, such as tests of normality and adjustment for multiple comparisons
☐	☒	A full description of the statistical parameters including central tendency (e.g. means) or other basic estimates (e.g. regression coefficient) AND variation (e.g. standard deviation) or associated estimates of uncertainty (e.g. confidence intervals)
☐	☒	For null hypothesis testing, the test statistic (e.g. *F*, *t*, *r*) with confidence intervals, effect sizes, degrees of freedom and *P* value noted<br>*Give P values as exact values whenever suitable.*
☒	☐	For Bayesian analysis, information on the choice of priors and Markov chain Monte Carlo settings
☒	☐	For hierarchical and complex designs, identification of the appropriate level for tests and full reporting of outcomes
☒	☐	Estimates of effect sizes (e.g. Cohen's *d*, Pearson's *r*), indicating how they were calculated

Our web collection on statistics for biologists contains articles on many of the points above.

Software and code

Policy information about availability of computer code

Data collection	The artists dataset is collected from art500K (https://deepart.ust.hk/ART500K/art500k.html), artnet (http://www.artnet.com/), artprice (https://www.artprice.com/) and findartinfo (http://www.findartinfo.com/english.html). The directors dataset is available at IMDB (https://www.imdb.com/). The scientists dataset is collected from Web of Science, which was obtained through a purchase agreement with Thomson Reuters, and Google Scholar profiles.
Data analysis	Data analyses were conducted using Python 3.6

For manuscripts utilizing custom algorithms or software that are central to the research but not yet described in published literature, software must be made available to editors and reviewers. We strongly encourage code deposition in a community repository (e.g. GitHub). See the Nature Portfolio guidelines for submitting code & software for further information.

Data

Policy information about availability of data

All manuscripts must include a data availability statement. This statement should provide the following information, where applicable:

- Accession codes, unique identifiers, or web links for publicly available datasets
- A description of any restrictions on data availability
- For clinical datasets or third party data, please ensure that the statement adheres to our policy

Data necessary to replicate results of this study will be made freely available.

Field-specific reporting

Please select the one below that is the best fit for your research. If you are not sure, read the appropriate sections before making your selection.

- [ ] Life sciences
- [x] Behavioural & social sciences
- [ ] Ecological, evolutionary & environmental sciences

For a reference copy of the document with all sections, see nature.com/documents/nr-reporting-summary-flat.pdf

Behavioural & social sciences study design

All studies must disclose on these points even when the disclosure is negative.

Study description	This is a quantitative study of individual career trajectories and creative products based on pre-existing datasets
Research sample	We collected three large-scale datasets from three domains: (1) 3,480 artists' profiles from artprice and findartinfo and 800K images of visual arts from art500k and artnet from renaissance to modern art; (2) 4,377 directors' profiles and 79K movies with plots and cast information from the IMDB database; (3) 20,040 scientists' profiles and over 1 million publication records from Web of Science and Google Scholar
Sampling strategy	No statistical methods were used to predetermine sample size.
Data collection	This study is based on pre-existing datasets.
Timing	Profiles of artists and directors were collected in spring 2017. Image datasets were collected in summer 2019. Web of Science and Google Scholar were collected in summer 2015.
Data exclusions	The analysis has no data exclusions. Selection criteria within a dataset are described in the supplementary information.
Non-participation	There are no participants in this study.
Randomization	This is a data driven study, not a randomized experiment.

Reporting for specific materials, systems and methods

We require information from authors about some types of materials, experimental systems and methods used in many studies. Here, indicate whether each material, system or method listed is relevant to your study. If you are not sure if a list item applies to your research, read the appropriate section before selecting a response.

Materials & experimental systems

n/a	Involved in the study	
x		Antibodies
x		Eukaryotic cell lines
x		Palaeontology and archaeology
x		Animals and other organisms
x		Human research participants
x		Clinical data
x		Dual use research of concern

Methods

n/a	Involved in the study	
x		ChIP-seq
x		Flow cytometry
x		MRI-based neuroimaging
